# Blue Light-Mediated Multicomponent Synthesis of 5‑Amino-4-chalcogenyl-1*H*-pyrazoles

**DOI:** 10.1021/acs.joc.6c00605

**Published:** 2026-07-17

**Authors:** Pâmella Cordeiro, João Pedro S. S. C. Thomaz, Matheus G. Theodorio, Thiago J. Peglow, Thiago Barcellos, Antonio L. Braga, Vanessa Nascimento

**Affiliations:** † LabSelen, Department of Chemistry, Universidade Federal de Santa Catarina, Florianópolis, Santa Catarina 88040-900, Brazil; ‡ SupraSelen Laboratory, Department of Organic Chemistry, 28110 Universidade Federal Fluminense, Campus do Valonguinho, Niterói, Rio de Janeiro 24020-141, Brazil; § Biotechnology Laboratory for Natural and Synthetic Products, 58802 University of Caxias do Sul, Caxias do Sul, Rio Grande do Sul 95070-560, Brazil

## Abstract

Visible-light-mediated multicomponent reactions offer a simple and efficient approach for constructing functionalized heterocycles. Herein, we report a photocatalyst-free, iodine-mediated multicomponent protocol for the synthesis of 5-amino-4-chalcogenyl-1*H*-pyrazoles from 3-oxo-3-phenylpropanenitriles, arylhydrazines, and diorganyl dichalcogenides under blue-LED irradiation. The transformation proceeds without transition metals, external oxidants, or additives. Notably, the same optimized reaction conditions enable access to both selenium- and sulfur-containing derivatives without reoptimization, demonstrating the robustness and generality of the methodology. The protocol exhibits a broad substrate scope, enabling the preparation of more than 30 structurally diverse examples and tolerating electron-donating and -withdrawing substituents on both the dichalcogenide and hydrazine partners. Selenium derivatives were obtained in excellent yields, while disulfide analogues were also formed in high yields (up to 98). The synthetic utility was further demonstrated through gram-scale reactions, crossover experiments, and downstream derivatization via the Clauson-Kaas reaction. Control and radical-trapping experiments support a visible light-induced radical pathway, where photoirradiation is responsible for radical generation, enabling the formation of chalcogen-centered species. Overall, this study provides a straightforward strategy for the synthesis of chalcogen-functionalized pyrazoles under mild, metal-free conditions.

## Graphical abstract

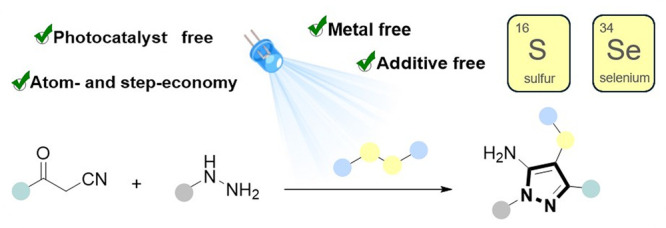

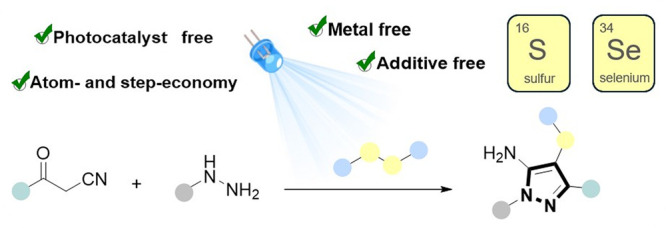

## Introduction

Pyrazole derivatives represent a class of heterocycles of great interest due to their wide range of applications in different areas of chemistry and materials science.^1,2^ Structurally, pyrazoles feature a five-membered aromatic ring containing two vicinal nitrogen atoms with distinct electronic characters, which govern tautomerism, hydrogen-bonding behavior, and metal-coordination properties.^3^ In addition to their well-established pharmacological relevance, associated with anti-inflammatory, anticancer, and antimicrobial activities, these structures have increasingly attracted attention as building blocks in organic synthesis, serving as precursors for more complex heteroaromatic systems (Figure 1).^4–11^ From an applied perspective, pyrazole derivatives are widely exploited not only as ligands in homogeneous catalysis and components of optical and electrochemical sensors but also as key motifs in commercial agrochemicals, including fungicides, herbicides, and insecticides. This versatility arises from the aromatic nature of the heterocyclic core combined with the possibility of site-selective functionalization, which allows for fine modulation of electronic, photophysical, and coordination properties. In particular, aminopyrazoles constitute an important subclass, widely explored as key building blocks in medicinal chemistry and materials science because of their rich functionalization patterns and diverse biological activities.^12,13^

**Figure 1:**
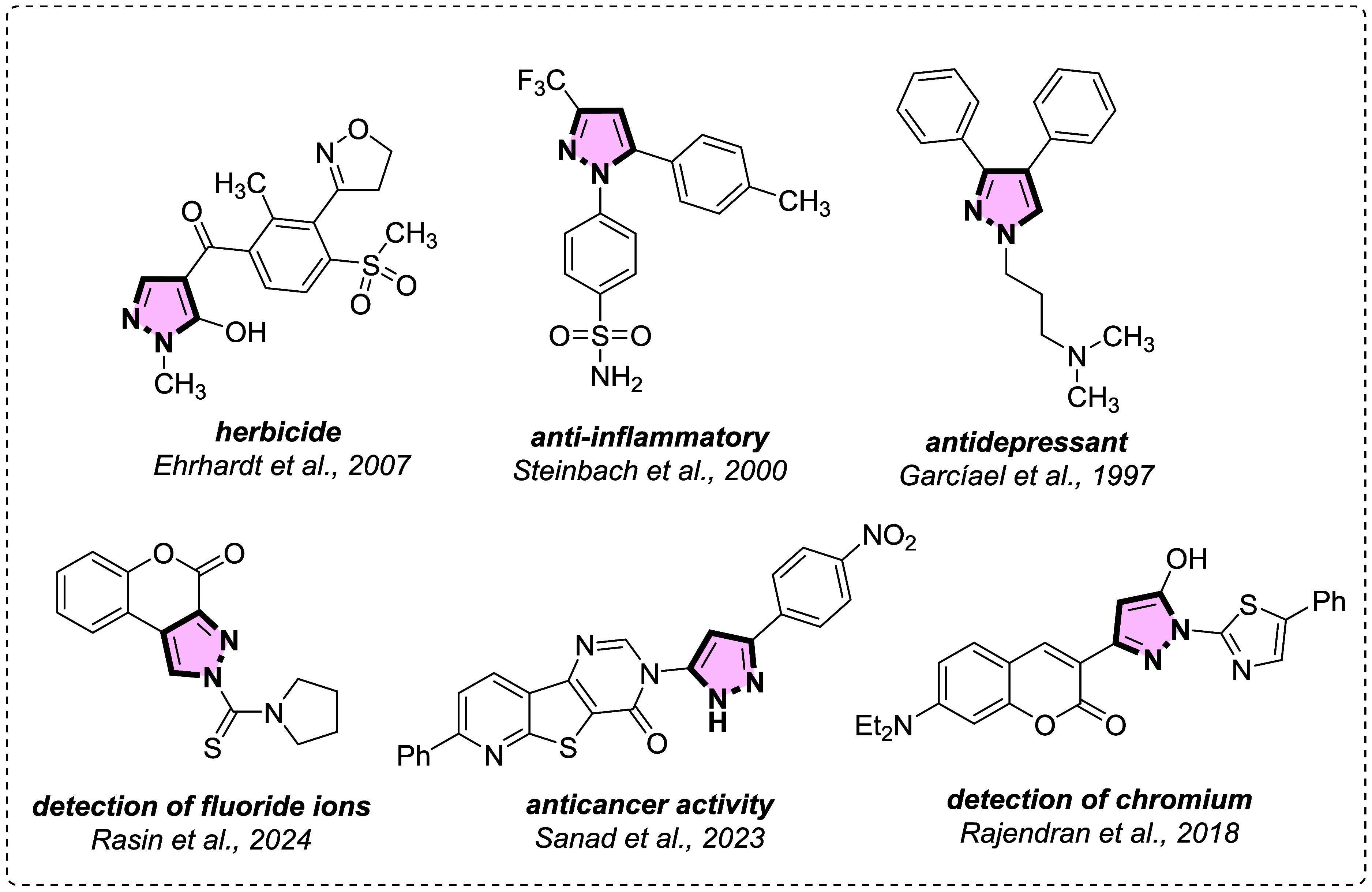
Examples of pyrazole-containing compounds and their applications.

The functionalization of heterocyclic scaffolds with chalcogen elements has become an effective approach to modulate molecular structure and properties.^14–16^ Because of their high polarizability and ability to engage in directional noncovalent interactions, such as chalcogen bonding, sulfur, selenium, and tellurium play a central role in controlling reactivity and molecular recognition.^17–19^ In synthetic chemistry, organochalcogen compounds are well-established as organocatalysts, intermediates, and reagents in selective transformations.^20^ As observed for pyrazole derivatives, the deliberate introduction of chalcogen atoms into heterocyclic architectures affords highly tunable molecular systems, reinforcing their value in the development of advanced functional materials and technological relevance.^21–25^

Given this broad spectrum of applications, increasing attention has been directed toward the chalcogen functionalization of pyrazole frameworks, especially the synthesis of 4-arylselenyl-1*H*-pyrazoles. Reported methods include reactions with selenium electrophiles, transition-metal catalysis, selenium halides, and a visible-light protocol employing Rose Bengal as a photocatalyst (Scheme 1a),^26–28^ which, although efficient, relies on additional chemical inputs. However, existing methodologies are largely limited to selenium or sulfur individually. Sulfur derivatives usually require harsh thermal conditions, while visible-light approaches remain restricted to selenium and still depend on photocatalysts.^29–32^ These limitations contrast with green chemistry principles and the United Nations Sustainable Development Goals (SDGs), notably those related to responsible consumption and production (SDG 12), industrial, innovation and infrastructure (SDG 9), and reduction of environmental impact.^33,34^ Consequently, the development of broader and more sustainable methodologies for the synthesis of chalcogen-functionalized pyrazoles remains highly desirable.

**Scheme 1:**
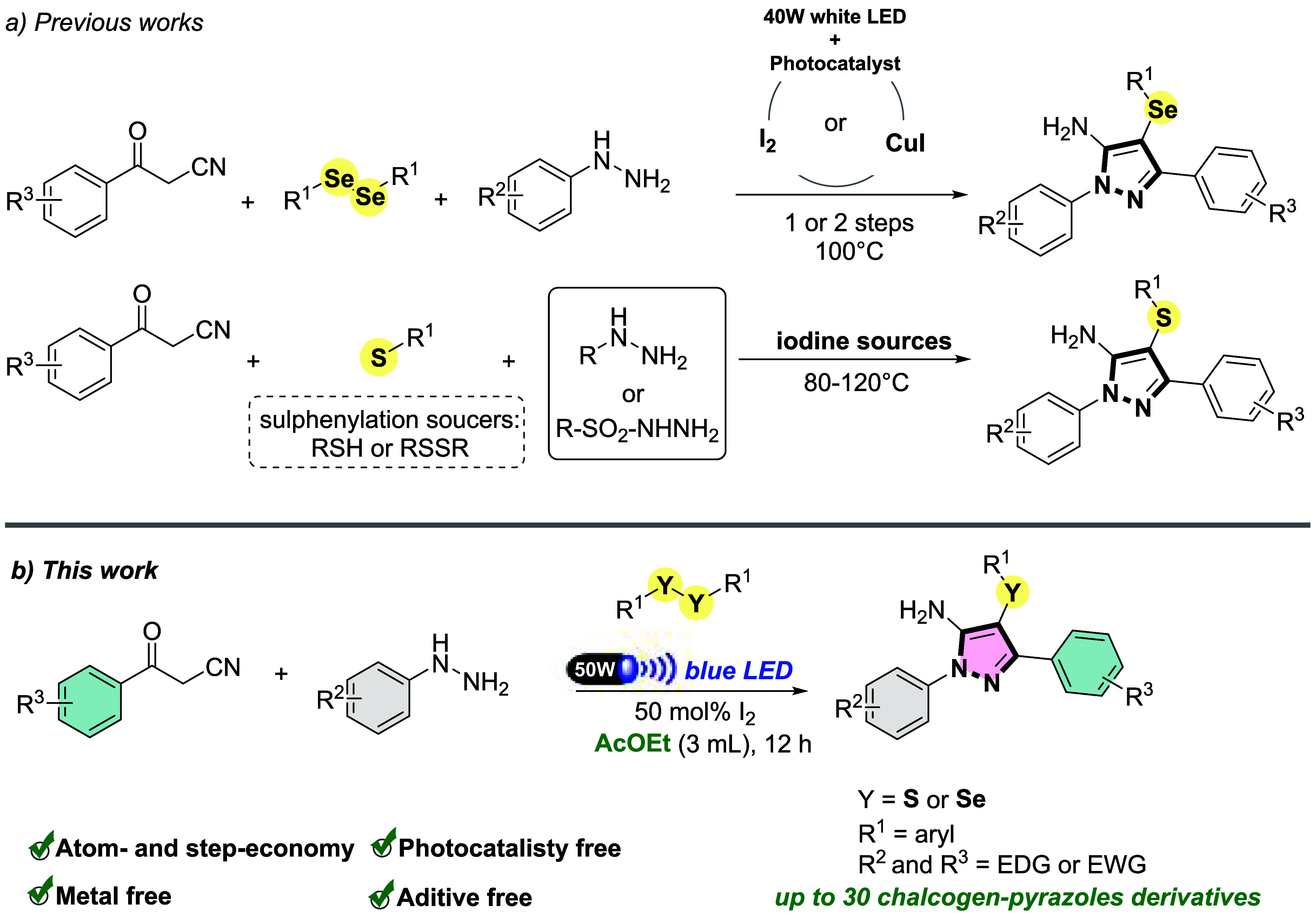
Synthesis of 4-Arylchacogenyl-1*H*-pyrazoles; (a) Previous Works; (b) This Work

In this context, multicomponent reactions emerge as powerful alternatives, offering significant advantages in organic synthesis, such as atom economy, operational simplicity, structural diversity, and reduced waste generation.^35–39^ The combination of multicomponent strategies with visible-light irradiation has also proven effective; however, the most desirable approach avoids the use of photocatalysts, which, although efficient, present drawbacks related to cost, potential toxicity, and challenges in removal or recycling, ultimately diminishing the overall greenness of the process.^40–42^ Building upon these principles, in this work, we report a multicomponent approach using visible light, photocatalyst-free, for the synthesis of chalcogen-containing pyrazoles, namely 4-arylselanyl-1*H*-pyrazoles as well as their sulfur-containing analogues. The transformation proceeds in the presence of catalytic iodine, without the need for additional additives, and employs ethyl acetate as a greener solvent, highlighting its potential as an environmentally friendly and operationally simple method (Scheme 1b).

## Results and Discussion

The initial reaction conditions were selected based on previously reported conventional chalcogenation protocols employing molecular iodine as a radical promoter/oxidative mediator in combination with organic photocatalysts.^33^ Thus, a mixture of **1** (0.5 mmol), **2a** (0.7 mmol), and **3a** (0.5 mmol) was irradiated with a 50 W blue LED in acetonitrile, in the presence of iodine (50 mol %) and Eosin Y. After 12 h, the desired product (**4a**) was obtained in 72% yield (Table 1, entry 1), while Rose Bengal provided a slightly higher yield of 76% (Table 1, entry 2). Encouraged by these results, we next investigated whether the reaction could proceed efficiently in the absence of light by evaluating different additives under thermal conditions (Table 1, entries 3–7). While most additives did not significantly improve the yield, FeCl_3_ proved to be particularly effective, affording the target compound in 84% yield under light-free conditions. Given the promising performance of FeCl_3_ alone, we then examined whether a combined system (photocatalyst + FeCl_3_ under irradiation) could further enhance the reaction efficiency. However, no synergistic effect was observed: the combination of Eosin Y and FeCl_3_ resulted in a decreased yield (57%, Table 1, entry 8), whereas Rose Bengal with FeCl_3_ furnished a 75% yield (Table 1, entry 9). These findings demonstrate that using FeCl_3_ as a single additive under light-free conditions is more efficient than using photocatalytic systems or combined approaches. Importantly, this outcome is advantageous from a green chemistry perspective as it eliminates the need for continuous light irradiation and avoids the use of relatively expensive organic photocatalysts, thereby simplifying the protocol and improving its economic and operational sustainability. Reducing the FeCl_3_ loading improved the yield to 90% (Table 1, entry 10), and omitting it completely further increased the yield to 92% (Table 1, entry 11), simplifying the process.

**Table 1:**
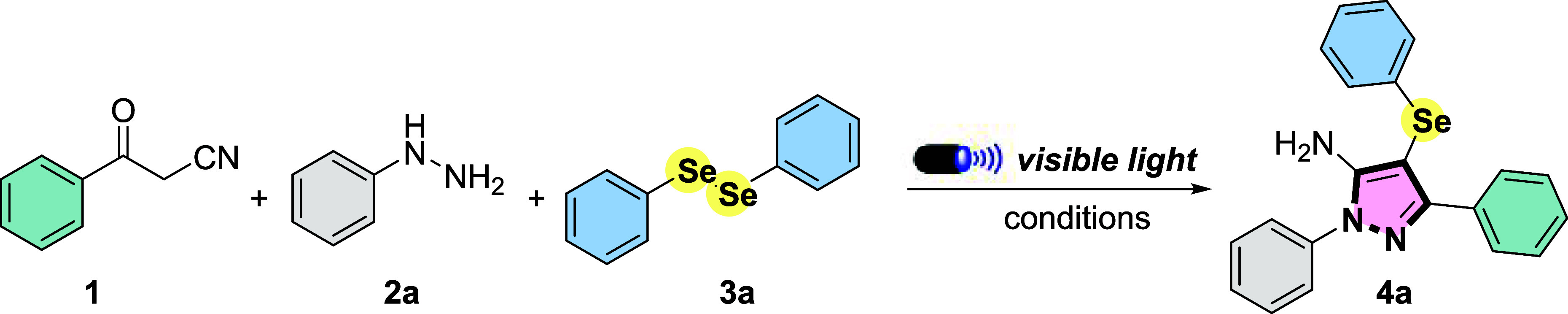
Optimization of Reaction Conditions^a^ ^,^ ^b^

#	Visible light	Iodine source (50 mol %)	Photocatalyst	Aditive	Solvent	Time (h)	Yield (%)
1	Blue LED 50W	I_2_	Eosin Y	-	CH_3_CN	12	72
2	Blue LED 50W	I_2_	Rose Bengal	-	CH_3_CN	12	76
3	Blue LED 50W	I_2_	-	InCl_3_	CH_3_CN	12	19
4	Blue LED 50W	I_2_	-	SnCl4	CH_3_CN	12	N.R.
5	Blue LED 50W	I_2_	-	SnCl_2_.H_2_O	CH_3_CN	12	22
6	Blue LED 50W	I_2_	-	BF_3_OEt_2_	CH_3_CN	12	20
7	Blue LED 50W	I_2_	-	FeCl_3_	CH_3_CN	12	84
8	Blue LED 50W	I_2_	Eosin Y	FeCl_3_	CH_3_CN	12	57
9	Blue LED 50W	I_2_	Rose Bengal	FeCl_3_	CH_3_CN	12	75
10^c^	Blue LED 50W	I_2_	-	FeCl_3_	CH_3_CN	12	90
11	Blue LED 50W	I_2_	-	-	CH_3_CN	12	92
12	Blue LED 50W	I_2_	-	-	CH_3_CN	24	37
13	Blue LED 50W	I_2_	-	-	CH_3_CN	6	43
14	Green LED 50W	I_2_	-	-	CH_3_CN	12	37
15	Blue LED 100W	I_2_	-	-	CH_3_CN	12	58
16	Blue LED 50W	KIO_3_	-	-	CH_3_CN	12	N.R.
17^f^	Blue LED 50W	others	-	-	CH_3_CN	12	Traces
18	Blue LED 50W	NIS	-	-	CH_3_CN	12	44
19^d^	Blue LED 50W	I_2_	-	-	CH_3_CN	12	58
20	Blue LED 50W	-	-	-	CH_3_CN	12	N.R.
21	Blue LED 50W	I_2_	-	-	AcOEt	12	96
22^g^	Blue LED 50W	I_2_	-	-	others	12	Low
23^e^	-	I_2_	-	-	AcOEt	12	45

a In a round-bottomed tube, 3-oxo-3-phenylpropanenitrile 1 (0.5 mmol), hydrazine 2a (0.7 mmol), diphenyl diselenide 3a (0.5 mmol), and solvent (3.0 mL) were added.

b Yields obtained after column chromatography.

c Reaction performed using FeCl_3_ (5 mol %).

d Reaction performed using I_2_ (25 mol %).

e In the absence of light (in the dark).

f Using KIO_4_, CuI, or KI, only trace amounts of the product were observed

g Using hexane (N.R.), toluene (52%), EtOH (84%), or THF (44%), lower yields were observed; N.R. = no reaction.

Next, we examined the effect of the reaction time. Interestingly, increasing the irradiation period to 24 h or reducing it to 6 h resulted in lower yields of 37% and 43%, respectively (Table 1, entries 12 and 13), indicating that 12 h is the optimal reaction time. Subsequently, the influence of the light source was also assessed. Using a green LED instead of a blue LED resulted in only a 37% yield (Table 1, entry 14), while increasing the power of the blue LED to 100 W also proved detrimental, giving a 58% yield (Table 1, entry 15). To investigate the role of iodine in the reaction, alternative iodine sources were tested (Table 1, entries 16–19), but none outperformed molecular iodine, which remained the most effective under the studied conditions. Moreover, reducing the loading of I_2_ from 50 mol % to 25 mol % decreased the yield to 58% (Table 1, entry 19). No reaction occurred in the absence of I_2_, which highlights its crucial role in the process.

Solvents such as hexane, toluene, ethanol, THF, acetonitrile, and ethyl acetate were screened to evaluate the solvent effect (Table 1, entry 22). Hexane completely suppressed the reaction, whereas toluene, ethanol, and THF afforded moderate yields (52%, 84%, and 44%, respectively). Acetonitrile, initially employed, provided a good yield (92%); however, ethyl acetate proved to be the most efficient solvent, delivering the product in a 96% yield (Table 1, entry 21). Under dark conditions, the reaction proceeded with a reduced yield of 45%. Thus, the optimal conditions correspond to those described in Table 1, entry 23, employing I_2_ (50 mol %) in ethyl acetate under blue LED irradiation for 12 h.

The generality of the optimized protocol was then evaluated using a series of substituted diselenides bearing electron-donating (EDG) and electron-withdrawing groups (EWG), as well as alkyl and heteroaromatic substituents (Scheme 2). The mesityl-substituted derivative (**4b**) was obtained in good yield (67%), whereas the *p*-methyl (**4c**) and *p*-methoxy (**4d**) substituent analogues showed no reactivity under the standard conditions. Notably, the naphthyl derivative (**4e**) furnished the desired product in excellent yield (80%), while the thiophene-substituted derivative (**4f**) afforded a moderate yield (45%). To further investigate the substrate scope, an alkyl-substituted diselenide was also evaluated, and the corresponding product was formed (**4g**) in 32% yield. Among the electron-withdrawing substituents, the *p*-chloro derivative (**4h**) performed remarkably well, yielding the product at 83%. The para-fluoro (**4i**) and trifluoromethyl (**4j**) analogues were also compatible, providing good yields of 65% and 56%, respectively. Furthermore, the scalability of the protocol was demonstrated by conducting the reaction on a 3.0 mmol scale under the optimized conditions, affording product **4a** in 91% yield after 12 h.

**Scheme 2:**
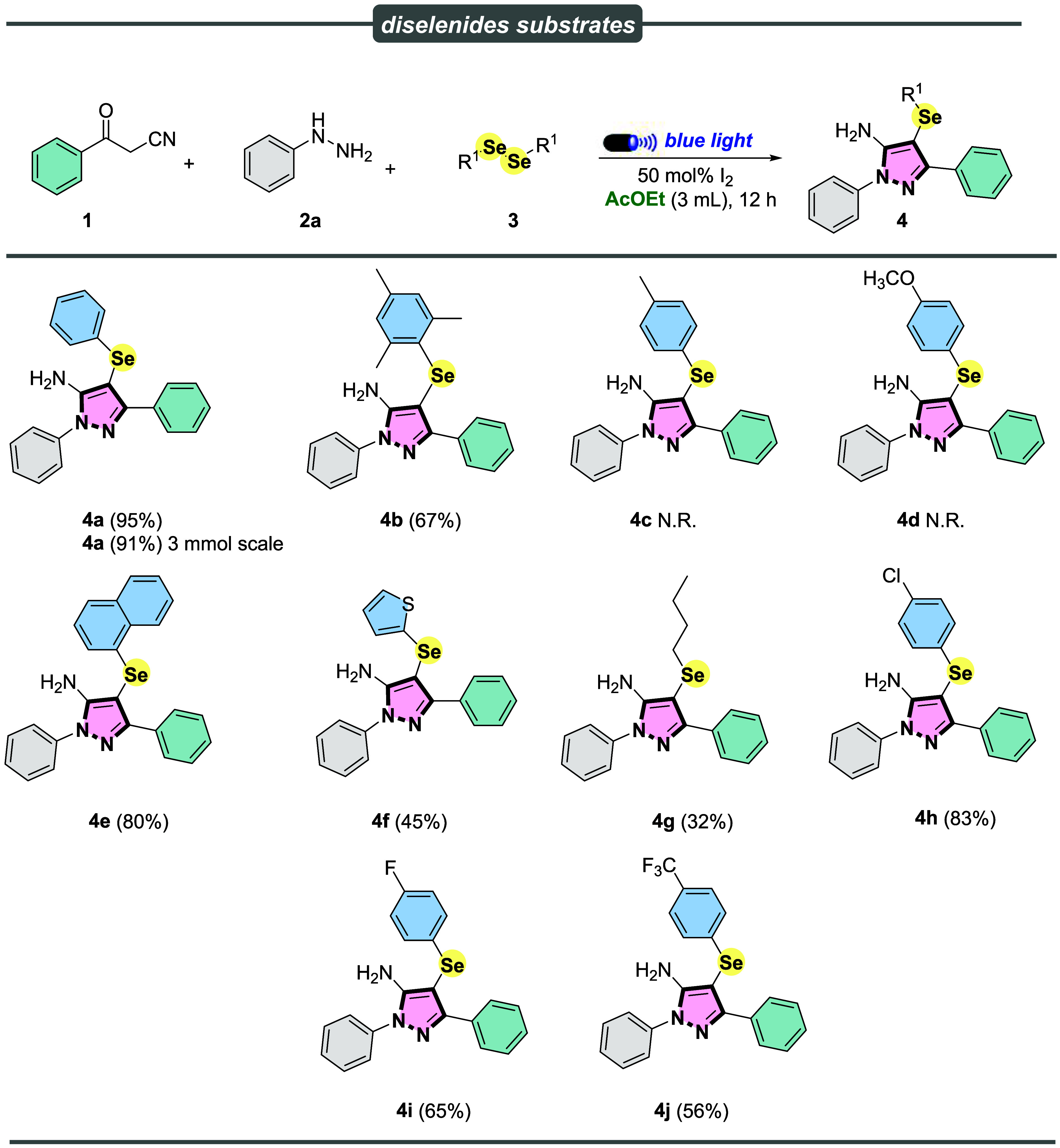
Substrate Scopes for 5-Amino-4-(arylselanyl)-1*H*-pyrazoles Using Different Diselenides^a^,^b^

a In a round-bottomed tube, 3-oxo-3-phenylpropanenitrile **1** (0.5 mmol), hydrazine **2a** (0.7 mmol), diselenide **3** (0.5 mmol), and solvent (3.0 mL) were added.
b Yields were obtained after column chromatography.

Encouraged by the excellent results achieved in the formation of selenium-functionalized products, we further explored the adaptability of the visible light-mediated chalcogen cyclization reaction using diorganyl disulfides and diorganyl ditellurides as substrates under the previously optimized reaction conditions (Scheme 3).

**Scheme 3:**
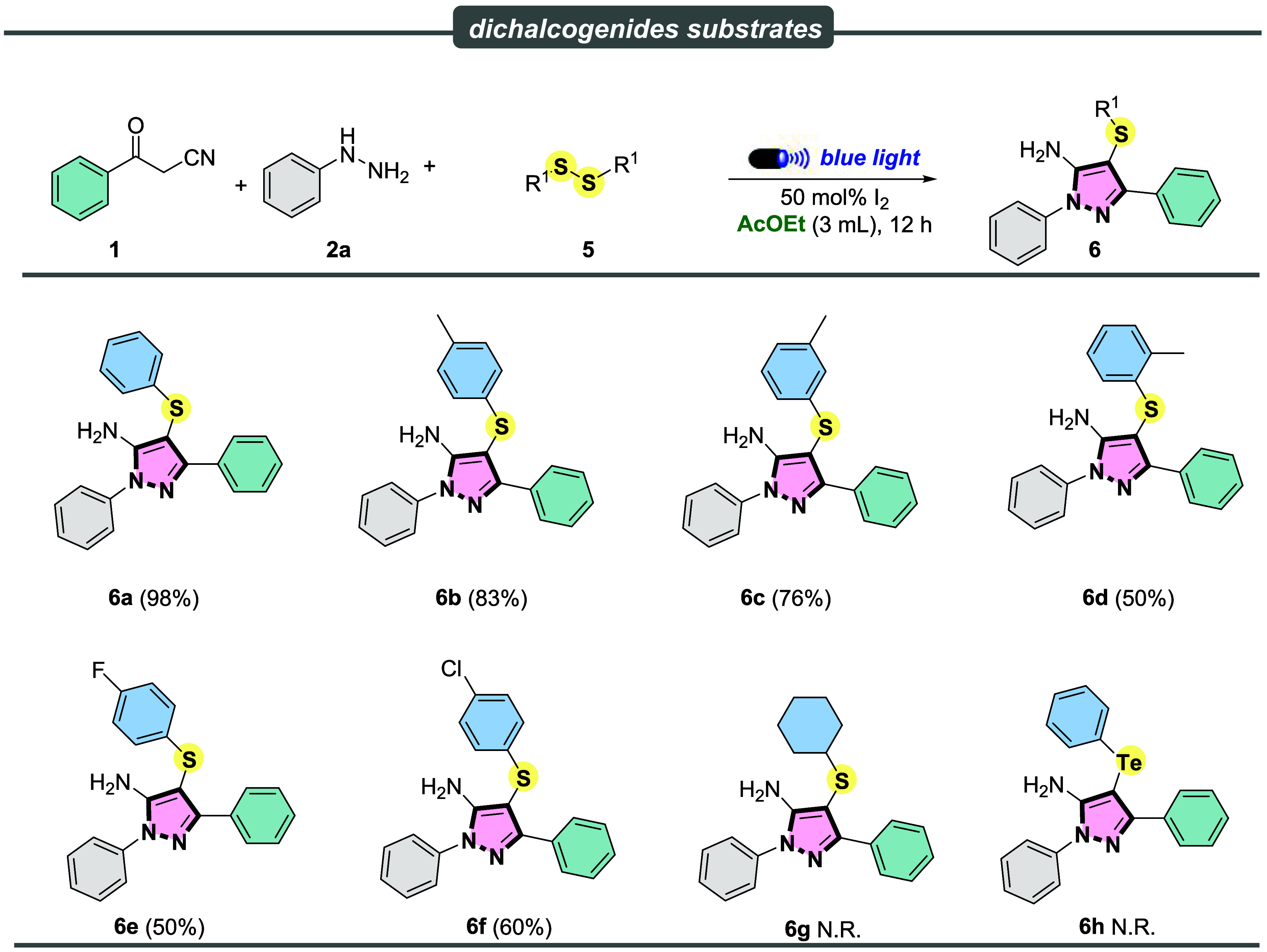
Substrate Scopes for 5-Amino-4-(arylselanyl)-1*H*-pyrazoles Using Different Disulfides^a^,^b^

a In a round-bottomed tube, 3-oxo-3-phenylpropanenitrile **1** (0.5 mmol), hydrazine **2a** (0.7 mmol), dichalcogenides **5** (0.5 mmol), and solvent (3.0 mL) were added.
b Yields obtained after column chromatography.

Notably, although the tellurium analogue (**6h**) was unreactive under the optimized conditions, the same protocol proved to be directly applicable to disulfides without any further modification of the reaction parameters. This operational simplicity highlights the robustness and generality of the method, enabling the selective functionalization of different chalcogen classes under identical conditions. In this series, excellent results were obtained, which is consistent with previous reports describing the higher reactivity of disulfides in related chalcogen transfer processes.^34^ Accordingly, the phenyl derivative afforded an outstanding 98% yield (**6a**). EDGs were generally more favorable, as demonstrated by the *p*-methyl (83%, **6b**), *m*-methyl (76%, **6c**), and *o*-methyl (50%, **6d**) derivatives. In contrast, EWGs resulted in lower efficiencies, with the *p*-fluoro (50%, **6e**) and *p*-chloro (60%, **6f**) analogues. Furthermore, a nonaromatic substrate (cyclohexyl, **6g**) was evaluated; however, no reaction was observed under the developed conditions, suggesting the importance of an aromatic group in the nitrile component.

Building upon the excellent performance of the visible-light-mediated chalcogen cyclization, we were encouraged by the robustness of the methodology. This prompted us to expand the compound library by exploring a broader set of hydrazine derivatives (Scheme 4), bearing both EDG and EWG substituents and applying selenium- and sulfur-containing precursors. In the case of *p*-methyl-substituted hydrazines, moderate reactivity was observed, giving a 30% yield for the selenium (**7a**) derivative and a 45% yield for the sulfur analogue (**8a**). For halogenated systems, the *p*-bromo substrate showed an opposite trend, with selenium performing significantly better (**7b**, 84%) compared to sulfur (**8b**, 35%), while the *m*-bromo derivative gave high conversions for both chalcogens (**7c**, Se, 68%; **8c**, S, 58%). Polyhalogenated hydrazines highlighted more evident substituent-dependent behavior: the 3,4-dichloro substrate delivered a low yield for selenium (**7d**, 29%) but remained productive for sulfur (**8d**, 53%), whereas the 3,5-dichloro analogue gave consistently good results (**7f**-Se, 68%; **8f**-S, 60%). Notably, the 2,5-dichloro and 2,4-dichloro isomers showed a marked decrease in reactivity for selenium (**7g**-Se, 18%; **8g**-S, 51%; **7h**-Se, 20%; **8h**-S, 48%). Increasing steric congestion appeared to be detrimental to the reaction outcome, as illustrated by the 2,6-dichloro derivatives (**7e** and **8e**), which were unreactive under the optimized conditions for both chalcogens. In contrast, strongly deactivating substituents did not uniformly suppress the reactivity. While para-fluoro (**7i** and **8i**) and ortho-nitro derivatives (**7j** and **8j**) failed to afford the desired products, likely due to electronic and positional effects that may hinder the formation or stabilization of reactive intermediates, the *p*-CF_3_ analogue displayed a notable chalcogen-dependent behavior, delivering a moderate 56% yield for selenium (**7k**) but only 21% for sulfur (**8k**). This unexpected divergence suggests that subtle electronic and chalcogen-specific effects may influence the efficiency of the transformation. Additionally, attempts to extend the methodology to alkyl-substituted hydrazines as well as to aliphatic nitrile derivatives were unsuccessful under the optimized conditions (**7l** and **7m**).

**Scheme 4:**
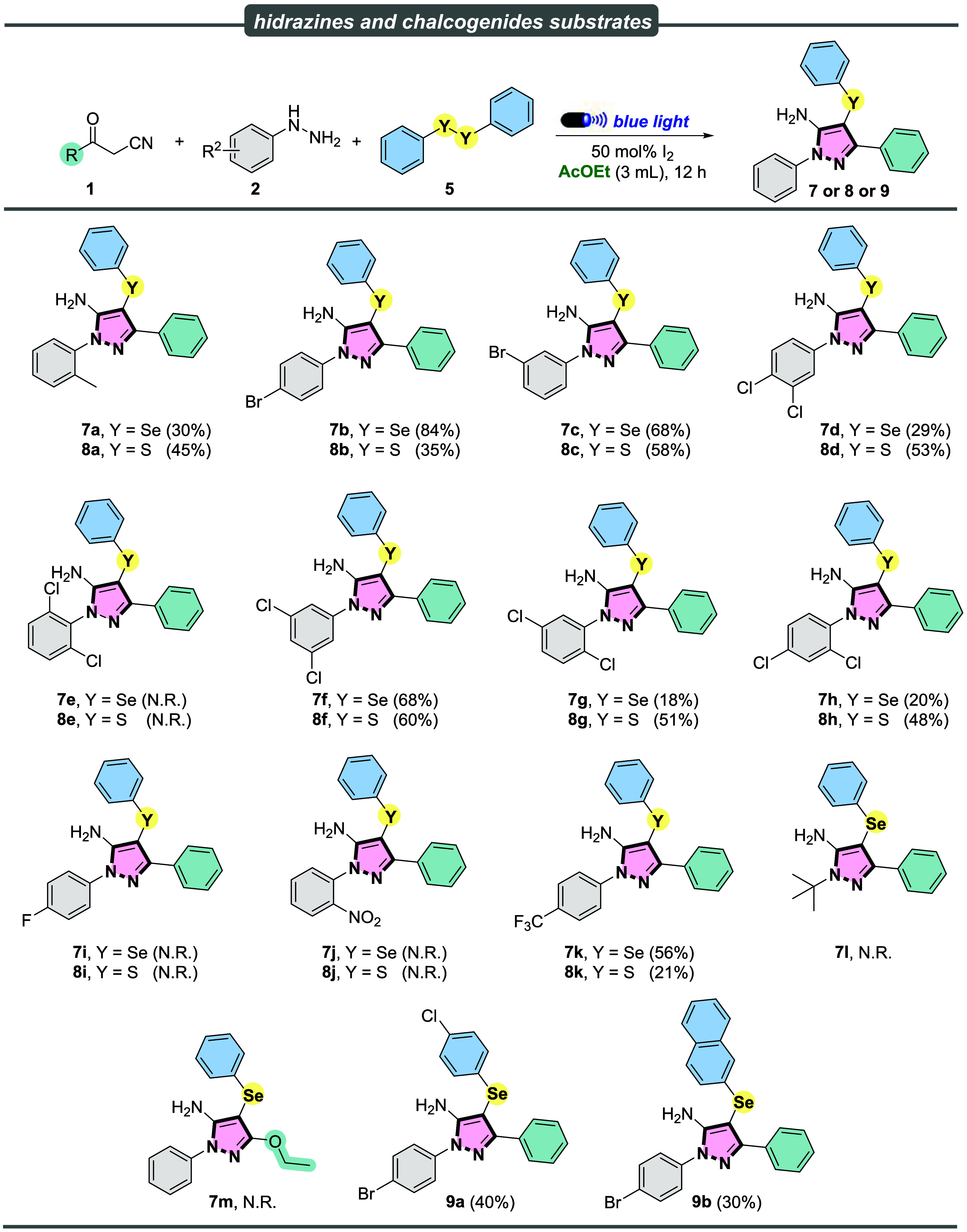
Substrate Scopes for 5-Amino-4-(arylchalcogenyl)-1*H*-pyrazoles^a^,^b^

a In a round-bottomed tube, 3-oxo-3-phenylpropanenitrile **1** (0.5 mmol), hydrazine 2 (0.7 mmol), dichalcogenide **5** (0.5 mmol), and solvent (3.0 mL) were added.
b Yields obtained after column chromatography.

In addition, we sought to further explore the synthetic potential of this photochemical protocol by introducing a crossover experiment, which, to the best of our knowledge, has not been investigated in closely related systems. Using meta-bromo hydrazine in combination with two distinct diselenides, para-chloro phenyl diselenide and naphthyl diselenide, the corresponding cross-cyclized products were obtained in 40% (**9a**) and 30% (**9b**) yield, respectively. These findings demonstrate that the transformation is not restricted to simple matched substrate pairs but can also accommodate mixed chalcogen sources, thereby expanding the structural diversity accessible through this methodology. Furthermore, compared to previously reported approaches, the current methodology combines operational simplicity with photocatalyst-free conditions and an innovative strategy that incorporates selenium and sulfur derivatives in the same protocol, as corroborated by sustainability metrics (see SI, page S2).

In order to gain further insight into the mechanism of the synthesis of 5-amino-4-(arylselanyl)-1*H*-pyrazole (**4a**), several preliminary control experiments were carried out (see Scheme 5). Initially, the reaction was performed under optimized conditions but in the absence of iodine, and no product (**4a**) was observed, indicating the essential role of iodine in the transformation (see Scheme 5a). This observation is consistent with the optimization studies (Table 1, entry 20), which showed that halving the iodine amount significantly decreased the yield. Next, radical-trapping experiments were performed using 2,2,6,6-tetramethyl-1-piperidinyloxy (TEMPO) and (2,6-di-*tert*-butyl-4-methylphenol) BHT, which afforded a 20% yield with TEMPO and only trace amounts with BHT, suggesting that the reaction proceeds predominantly via a radical pathway (Scheme 5b). To prove the stage at which iodine is crucial, the isolated byproduct (**10**) was subjected to the optimized conditions in the presence of diphenyl diselenide, affording the selenylated product (**4a**) in a 95% yield, confirming direct selenylation of this intermediate (Scheme 5c). Furthermore, the reaction between **1** and **2a** under the optimized conditions afforded compound **10** in an 85% yield (Scheme 5d), as expected, since this compound was identified as a byproduct during the reaction optimization studies. In addition, compound **1** was reacted with **3** under the optimized conditions in an attempt to verify whether a C–H chalcogenated intermediate could be accessed; however, no product formation was observed (Scheme 5e) Finally, when the reaction was carried out under an inert N_2_ atmosphere, the yield dramatically decreased to 30% (Scheme 5f), demonstrating the participation of from air in the course of the reaction.

**Scheme 5:**
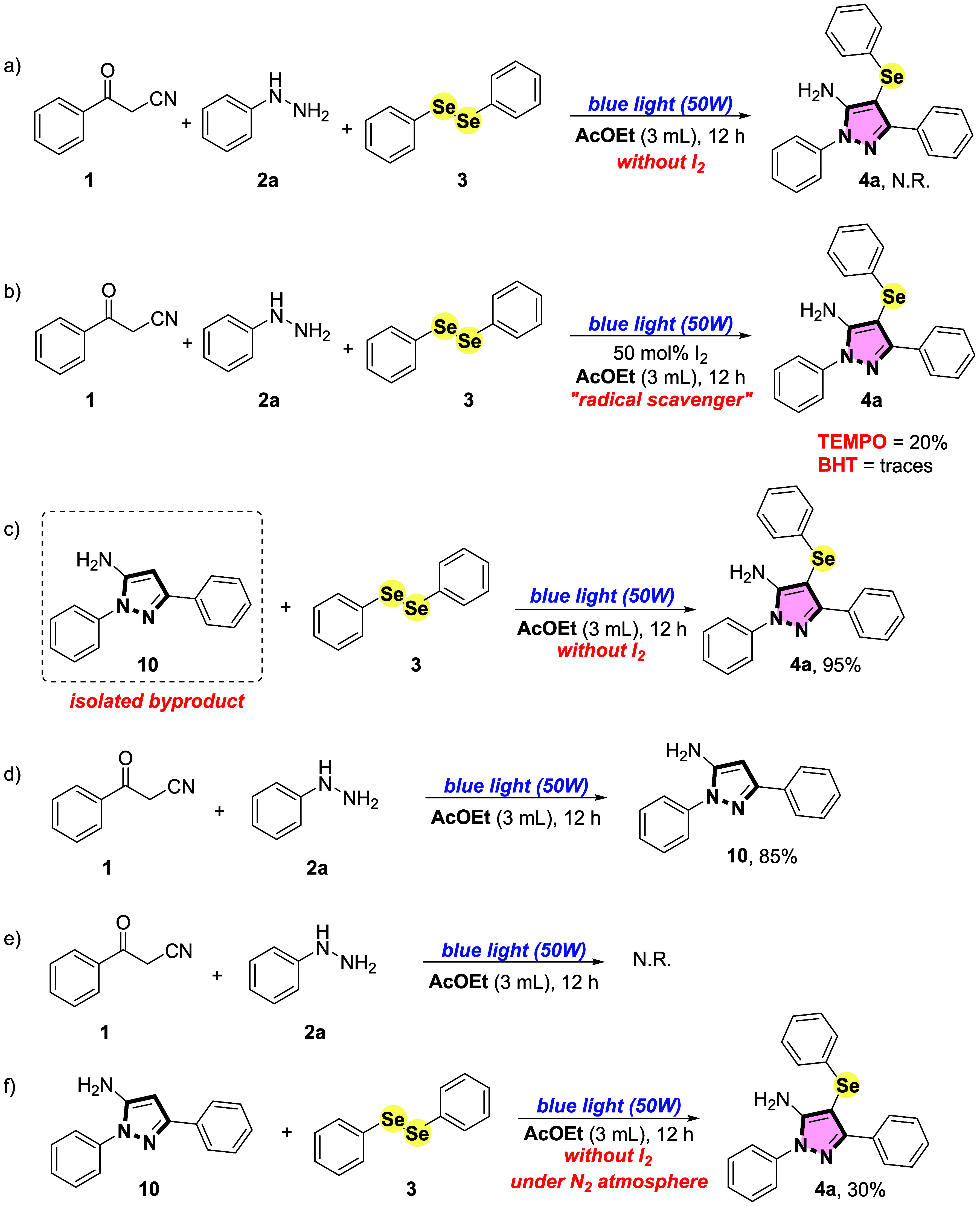
Control Experiments

On the basis of the results of our control experiments, as well as the previous literature,^33^ a reaction mechanism is proposed (Scheme 6). Initially, arylhydrazine (**2a**) reacts with benzoacetonitrile (**1**) through a 1,2-addition catalyzed by iodine acting as a Lewis acid, affording the hydrazone intermediate (**A**). This intermediate then undergoes intramolecular cyclization followed by oxidative aromatization, leading to the formation of 5-amino-1*H*-pyrazole **10**.^43–45^ Subsequently, the phenylselenyl radical (**B**) is generated under blue LED irradiation.^44,46–48^ Addition of radical (**B**) to compound (**10**) furnishes the radical intermediate (**C**), which is subsequently oxidized, likely by molecular oxygen from the air, to afford the cationic intermediate (**D**). Finally, deprotonation of (**D**) delivers the desired product (**4a**).

**Scheme 6:**
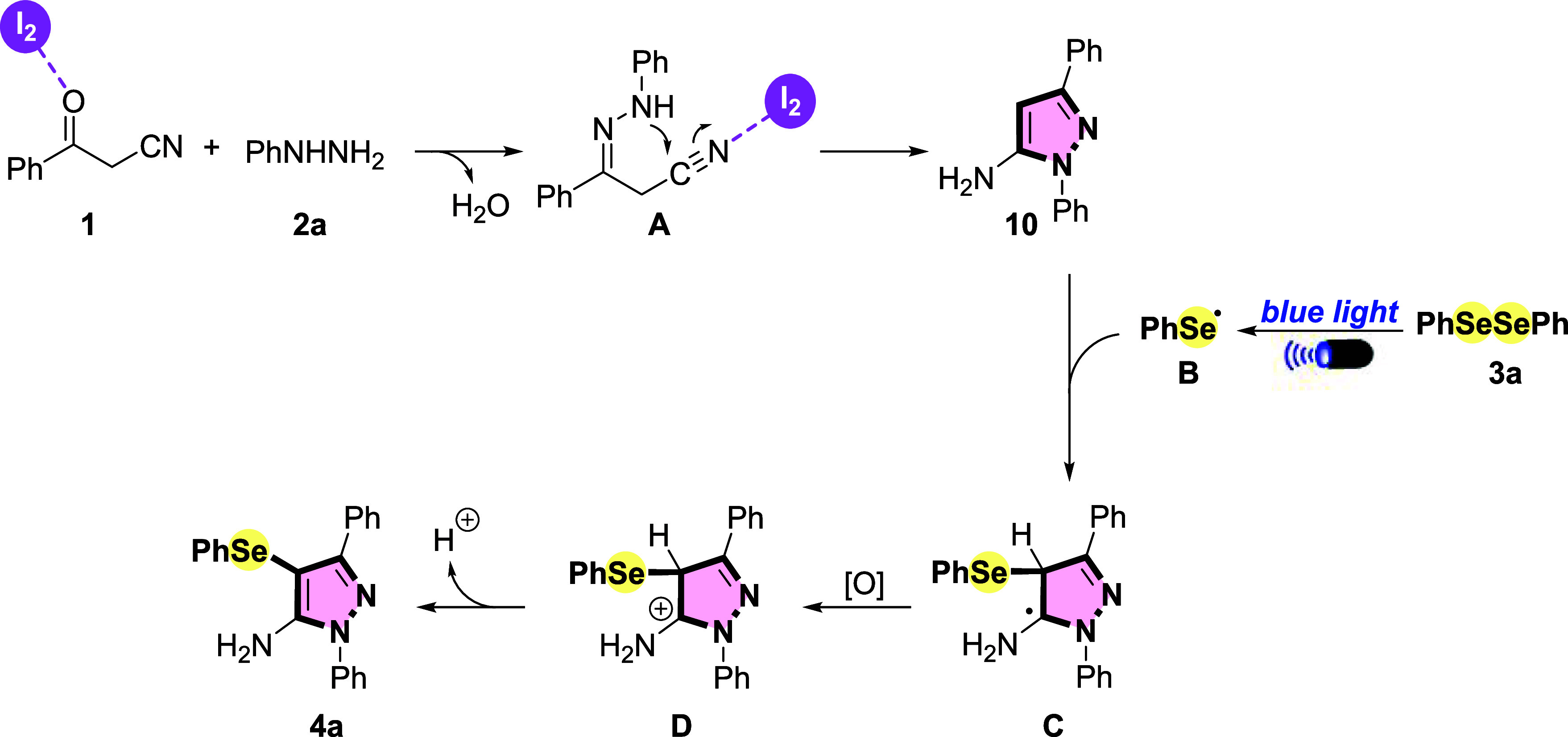
Plausible Mechanism for the Formation of 5-Amino-4-(arylselanyl)-1*H*-pyrazole (**4a**)

In the end, we turned our attention to demonstrating the synthetic application of the obtained chalcogenated pyrazoles and selected 5-amino-4-(phenylselanyl)-1*H*-pyrazole (**4a**) as a representative substrate. To further elaborate on this scaffold, we applied the classical Clauson-Kaas reaction, a well-established method for converting aromatic amines into *N*-substituted heterocycles using 2,5-dimethoxytetrahydrofuran (DMTHF) under acidic conditions.^49,50^ Accordingly, compound (**4a**) was stirred in DMTHF with acetic acid at 50 °C for 4 h, affording the pyrrole derivative (**11**) in an excellent 80% yield (Scheme 7). This successful derivatization demonstrates that the selenium-functionalized pyrazole core is fully compatible with downstream synthetic modifications, further underscoring the versatility of the methodology.

**Scheme 7:**
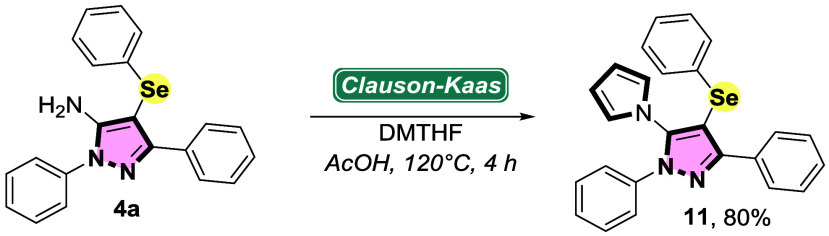
Synthesis of Pyrrole Derivative **11**

## Conclusion

In conclusion, we have developed a visible-light-driven, iodine-mediated multicomponent protocol for the efficient synthesis of 5-amino-4-chalcogenyl-1*H*-pyrazoles under mild reaction conditions. The methodology operates without photocatalysts, transition metals, or external oxidants, using ethyl acetate as a greener solvent and air as an oxidant. Notably, the same optimized conditions are applicable to both diselenides and disulfides without reoptimization, which underscores the robustness and generality of the protocol. A broad substrate scope was demonstrated, providing more than 30 structurally diverse derivatives in good to excellent yields, including gram-scale synthesis and cross-coupling transformations. Mechanistic investigations support a visible-light-induced radical pathway in which photoirradiation plays a crucial role in radical generation. Overall, this operationally simple and environmentally benign strategy provides a versatile platform for accessing chalcogen-functionalized pyrazoles with potential applications in medicinal and material chemistry.

## Experimental Section

### General Information

The reagents and solvents used in the synthesis were purchased from Sigma-Aldrich Brazil and LabSynth, all of which were used without prior purification. The reactions were monitored by thin-layer chromatography (TLC) using silica gel 60 F_254_ aluminum sheets, and the visualization of the spots was done under UV light (254 nm), stained with iodine, or by the mixture of 5% vanillin in 10% H_2_SO_4_ and heat as developing agents. Column chromatography was performed on silica gel (230–365 mesh). ^1^H NMR spectra were obtained on a Bruker Avance NEO 500 MHz instrument employing a direct broadband probe at 500 MHz. The spectra were recorded in CDCl_3_ solutions. The chemical shifts are reported in ppm, referenced to tetramethylsilane (TMS) as the internal reference. Coupling constants (*J*) are reported in Hertz. Abbreviations used to denote the multiplicity of a particular signal are s (singlet), d (doublet), dd (doublet of doublets), t (triplet), and m (multiplet). ^13^C­{^1^H} NMR spectra were obtained on a Bruker Avance NEO 500 MHz employing a direct broadband probe at 125 MHz. The chemical shifts are reported in ppm, referenced to the solvent peak of CDCl_3_ (δ 77.0 ppm). The melting points of the substances were determined using a Fisatom apparatus (Mod. 430D) series 209219. High-resolution electrospray ionization mass spectrometry (ESI-QTOF) analyses were performed on a Bruker Daltonics micrOTOF-Q II instrument operating in positive mode. Samples were dissolved in HPLC-grade methanol containing 0.1% formic acid and introduced into the ionization source by means of a syringe pump at a flow rate of 5.0 μL/min. Data acquisition and processing were carried out using Compass 1.3 for micrOTOF-Q II software (Bruker Daltonics, Billerica, MA, USA).

**General procedure for the synthesis of 5-amino-4-chalcogenyl-1** *H* **-pyrazoles 4, 7–9:** By a multicomponent reaction, benzoylacetonitrile **1** (0.5 mmol), hydrazine **2** (1.4 equiv., 0.7 mmol), chalcogenide substrates **3** or **5** (1 equiv., 0.5 mmol), and I_2_ (50 mol %) in 3 mL of AcOEt were added in a single tube at room temperature for 12 h under magnetic stirring and a blue LED (50W). After that, the remaining iodine was neutralized with saturated Na_2_S_2_O_3_ solution and extracted with ethyl acetate (3 x 10 mL). The organic phase was separated, dried over Na_2_SO_4_, filtered, and the solvent evaporated under reduced pressure. The crude material was further purified by column chromatography (hexanes/ethyl acetate) on silica gel.

*Note*: In the large-scale reaction, **1a** (3 mmol, 0.435 g), **2a** (4.2 mmol, 0.321 g), **3a** (3 mmol, 0.942 g), and I_2_ (50 mol %, 0.381 g) were used in 18 mL of AcOEt, and product **4a** was obtained at 91% yield (1.061 g).

**Caution!** Hydrazine is toxic and corrosive, and iodine is corrosive and can cause skin and eye irritation. For safety, all manipulations involving these reagents were performed in a well-ventilated fume hood using appropriate personal protective equipment. LED irradiation can irritate the eyes; therefore, appropriate eye protection was used during all of the photochemical experiments.

**1,3-Diphenyl-4-(phenylselanyl)-1** *H* **-pyrazol-5-amine (4a):** ^26^ Purified by column chromatography (hexane/ethyl acetate = 97:3); Yield: 0.186 g (95%); Yellow solid, m.p: 124–125 °C; ^1^H NMR (CDCl_3_, 500 MHz) δ (ppm) = 7.93–7.91 (d, *J* = 10.0 Hz, 2H); 7.64 (d, *J* = 5.0 Hz, 2H); 7.45 (t, *J* = 5.0 Hz 2H); 7.32 (t, J = 5.0 Hz, 3H); 7.28 (d, J = 5.0 Hz, 2H); 7.25 (d, J = 5.0 Hz, 2H); 7.18 (t, J = 5.0, 2H); 7.11 (t, J = 5.0 Hz, 1H); 4.30 (s, 2H). ^13^C­{^1^H} NMR (CDCl_3_, 126 MHz) δ (ppm) = 153.6, 149.5, 138.7, 133.1, 133.0, 129.6, 129.3, 128.2, 128.2, 127.8, 127.6, 125.9, 123.6, 82.4.

**4-(Mesitylselanyl)-1,3-diphenyl-1** *H* **-pyrazol-5-amine (4b)**: Purified by column chromatography (hexane/ethyl acetate = 97:3); Yield: 0.207 g (67%); Brown oil; ^1^H NMR (CDCl_3_, 500 MHz) δ (ppm) = 7.80 (d, J = 5.0 Hz, 2H); 7.59 (d, J = 10.0 Hz, 2H); 7.45 (t, J = 10.0 Hz 2H); 7.37–7.30 (m, 4H); 4.01 (s, 2H); 2.34 (s, 6H); 2.21 (s, 3H). ^13^C­{^1^H} NMR (CDCl_3_, 126 MHz) δ (ppm) = 153.5, 147.6, 141.6, 138.6, 137.6, 133.6, 129.5, 129.0, 128.2, 128.1, 127.9, 127.9, 127.5, 123.7, 85.0, 24.0, 20.8. HRMS (ESI) *m*/*z*: [M + H]^+^ Calcd for C_24_H_23_N_3_SeH 434.1137; Found: 434.1126.

**4-(Naphthalen-1-ylselanyl)-1,3-diphenyl-1** *H* **-pyrazol-5-amine (4e)**: Purified by column chromatography (hexane/ethyl acetate = 97:3); Yield: 0.176 g (80%); Brown solid, m.p: 131–132 °C; ^1^H NMR (CDCl_3_, 500 MHz) δ (ppm) = 8.12 (d, J = 10.0 Hz, 1H); 7.94 (d, J = 5.0, 2H); 7.85 (d, *J* = 10.0 Hz, 1H); 7.71 (d, *J* = 5.0 Hz, 2H); 7.67 (d, *J* = 10.0 Hz, 1H); 7.56 (m, 4H); 7.50–7.56 (m, 4H); 7.38 (t, J = 5.0 Hz, 1H); 7.32–7.26 (m, 5H); 4.30 (s, 2H). ^13^C­{^1^H} NMR (CDCl_3_, 126 MHz) δ (ppm) = 154.0, 149.6, 138.7, 134.0, 132.9, 132.1, 131.5, 129.6, 128.6, 128.2, 128.2, 127.8, 127.7, 126.3, 126.3, 126.2, 126.1, 125.1, 125.0, 123.7, 81.0. HRMS (ESI) *m*/*z*: [M + H]^+^ Calcd for C_25_H_19_N_3_SeH 442.0818; Found: 442.0814.

**1,3-Diphenyl-4-(thiophen-2-ylselanyl)-1** *H* **-pyrazol-5-amine (4f)**: Purified by column chromatography (hexane/ethyl acetate = 97:3); Yield: 0.089 g (45%); Brown oil; ^1^H NMR (CDCl_3_, 500 MHz) δ (ppm) = 8.00 (d, *J* = 10.0 Hz, 2H); 7.64 (d, *J* = 5.0 Hz, 2H); 7.49 (d, *J* = 5.0 Hz, 2H); 7.42 (d, *J* = 10.0 Hz, 2H); 7.38–7.35 (m, 2H); 7.26 (d, *J* = 5.0 Hz, 1H); 7.06 (d, J = 3.5 Hz, 1H); 6.91–6.89 (dd, *J* = 3.5, 5.0 Hz, 1H); 4.39 (s, 2H). ^13^C­{^1^H} NMR (CDCl_3_, 126 MHz) δ (ppm) = 146.6, 138.1, 136.5, 133.4, 132.4, 131.5, 129.5, 129.1, 129.1, 127.4, 126.4, 125.8, 103.3. MS (APCI) *m*/*z*: [M + H]^+^ Calcd for C_19_H_15_N_3_SSeH 398.02; Found 398.41.

**4-(Butylselanyl)-1,3-diphenyl-1** *H* **-pyrazol-5-amine (4g)**: Purified by column chromatography (hexane/ethyl acetate = 97:3); Yield: 0.052 g (32%); Brown oil; ^1^H NMR (CDCl_3_, 500 MHz) δ (ppm) = 8.08 (d, *J* = 10.0 Hz, 2H); 7.66 (d, *J* = 5.0 Hz, 2H); 7.49 (t, *J* = 10.0 Hz, 2H); 7.41 (t, *J* = 10.0 Hz, 2H); 7.36–7.32 (m, 2H), 4.30 (s, 2H), 2.56 (t, *J* = 5.0 Hz, 2H), 1.56–1.50 (m, 2H), 1.36–1.28 (m, 2H), 0.80 (t, *J* = 5.0 Hz, 3H). ^13^C­{^1^H} NMR (CDCl_3_, 126 MHz) δ (ppm) = 153.0, 149.0, 138.9, 133.6, 129.5, 128.0, 127.9, 127.9, 127.4, 123.5, 83.7, 32.2, 29.2, 22.7, 13.5. HRMS (ESI) *m*/*z*: [M + H]^+^ Calcd for C_19_H_21_N_3_SeH 372.0974; Found: 372.0968.

**4-((4-Chlorophenyl)­selanyl)-1,3-diphenyl-1** *H* **-pyrazol-5-amine (4h)**:^26^ Purified by column chromatography (hexane/ethyl acetate = 97:3); Yield: 0.176 g (83%); Brown oil; ^1^H NMR (CDCl_3_, 500 MHz) δ (ppm) = 7.89 (d, *J* = 5.0 Hz, 2H); 7.62 (d, *J* = 5.0 Hz, 2H); 7.45 (t, *J* = 5.0 Hz, 2H); 7.32–7.21 (m, 4H); 7.00–7.97 (m, 4H), 4.23 (s, 2H). ^13^C­{^1^H} NMR (CDCl_3_, 126 MHz) δ (ppm) = 152.6, 148.4, 137.6, 131.8, 130.9, 130.3, 128.6, 128.3, 128.1, 127.3, 127.2, 126.7, 122.7, 81.1.

**4-((4-Fluorophenyl)­selanyl)-1,3-diphenyl-1** *H* **-pyrazol-5-amine (4i)**: Purified by column chromatography (hexane/ethyl acetate = 97:3); Yield: 0.133 g (65%); Brown solid, m.p: 123–124 °C; ^1^H NMR (CDCl_3_, 500 MHz) δ (ppm) = 7.92 (d, *J* = 5.0, 2H); 7.68 (d, *J* = 10.0 Hz, 2H); 7.51 (t, *J* = 10.0 Hz, 2H); 7.40–7.31 (m, 4H); 7.25–7.21 (m, 2H); 6.93 (t, *J* = 10.0, 2H); 4.32 (s, 2H). ^13^C­{^1^H} NMR (CDCl_3_, 126 MHz) δ (ppm) = 162,7, 160.7 (d, *J* = 250.0 Hz; C_C–F_), 153.5, 149.4, 138.6, 132.9, 129.8, 129.7, 129.6, 129.5, 128.4, 128.3, 128.2, 127.8, 127.4, 127.3 (d, *J*_*C–F*_ = 3.8 Hz; C_arom_), 125.6, 124.1, 123.7, 116.5, 116.3 (d, *J*_*C–F*_ = 21.3 Hz; C_arom_), 82.8. HRMS (ESI) *m*/*z*: [M + H]^+^ Calcd for C_21_H_16_FN_3_SeH 410.0573; Found: 410.0563.

**1,3-Diphenyl-4-((4-(trifluoromethyl)­phenyl)­selanyl)-1** *H* **-pyrazol-5-amine (4j)**: Purified by column chromatography (hexane/ethyl acetate = 97:3); Yield: 0.128 g (56%); Orange oil; ^1^H NMR (CDCl_3_, 500 MHz) δ (ppm) = 7.81 (d, *J* = 5 Hz, 2H); 7.61 (d, *J* = 5.0 Hz, 2H); 7.45 (t, *J* = 5.0 Hz, 2H); 7.38 (d, *J* = 10.0 Hz, 2H); 7.29–7.24 (m, 6H); 4.26 (s, 2H). ^13^C­{^1^H} NMR (CDCl_3_, 126 MHz) δ (ppm) = 152.6, 148.5, 137.62, 137.61 (d, *J*_*C–F*_ = 1.3 Hz; C_arom_), 137.4, 131.6, 128.7 (q, *J*_*C–F*_ = 245.0 Hz; C_C–F_), 128.5 (q, *J*_*C–F*_ = 29,3 Hz; C_arom_), 127.95, 127.90, 127.7, 127.4, 127.2, 127.14 (d, *J*_*C–F*_ = 3.6 Hz; C_arom_), 127.11, 127.0, 126.9, 126.7, 124.9 (q, *J*_*C–F*_ = 3.5 Hz; C_arom_), 124.6, 124.2, 123.1, 122.7, 122.3, 120.7, 80.15. HRMS (ESI) *m*/*z*: [M + H]^+^ Calcd for C_22_H_16_F_3_N_3_SeH 460.0535; Found: 460.0531.

**1,3-Diphenyl-4-(phenylthio)-1** *H* **-pyrazol-5-amine (6a)**:^30^ Purified by column chromatography (hexane/ethyl acetate = 97:3); Yield: 0.168 g (98%); Brow oil; ^1^H NMR (CDCl_3_, 500 MHz) δ (ppm) = 7.95 (d, *J* = 5.0 Hz, 2H); 7.68 (d, *J* = 10.0 Hz, 2H); 7.51 (t, *J* = 10.0 Hz, 2H); 7.38 (d, J = 10.0 Hz, 1H); 7.36–7.29 (m, 3H); 7.24 (t, *J* = 10.0 Hz, 2H); 7.16 (d, *J* = 5.0, 2H); 7.10 (t, *J* = 5.0 Hz, 1H); 4.30 (s, 2H). ^13^C­{^1^H} NMR (CDCl_3_, 126 MHz) δ (ppm) = 153.4, 149.5, 138.6, 138.1, 132.5, 129.6, 129.0, 128.3, 128.3, 128.2, 127.7, 127.5, 125.1, 125.1, 123.6, 86.3.

**1,3-Diphenyl-4-(p-tolylthio)-1** *H* **-pyrazol-5-amine (6b)**:^30^ Purified by column chromatography (hexane/ethyl acetate = 97:3); Yield: 0.148 g (83%); Beige solid, m.p: 126–127 °C; ^1^H NMR (CDCl_3_, 500 MHz) δ (ppm) = 7.89 (d, *J* = 10.0 Hz, 2H); 7.62 (d, J = 10.0 Hz, 2H); 7.44 (t, *J* = 5.0 Hz, 2H); 7.32 (d, *J* = 5.0 Hz, 1H); 7.29 (m, 3H); 7.01 (t, *J* = 5.0 Hz, 4H); 4.24 (s, 2H); 2.21 (s, 3H). ^13^C­{^1^H} NMR (CDCl_3_, 126 MHz) δ (ppm) = 153.3, 149.4, 138.6, 134.9, 134.5, 132.6, 129.8, 129.65, 128.2, 128.2, 127.7, 127.5, 125.3, 123.6, 86.8, 20.9.

**1,3-Diphenyl-4-(m-tolylthio)-1** *H* **-pyrazol-5-amine (6c)**: Purified by column chromatography (hexane/ethyl acetate = 97:3); Yield: 0.135 g (76%); Yellow solid, m.p: 127–128 °C; ^1^H NMR (CDCl_3_, 500 MHz) δ (ppm) = 7.95 (d, *J* = 10.0, Hz, 2H); 7.68 (d, *J* = 10.0 Hz, 2H); 7.50 (t, *J* = 5.0 Hz, 2H); 7.37 (d, *J* = 10.0 Hz, 1H); 7.35–7.27 (m, 3H); 7.12 (t, *J* = 5.0 Hz, 1H); 6.98 (s, 1H); 6.93 (dd, *J* = 10.0, 5.0 Hz, 2H); 4.29 (s, 2H); 2.27 (s, 3H). ^13^C­{^1^H} NMR (CDCl_3_, 126 MHz) δ (ppm) = 153.4, 149.5, 138.8, 138.6, 137.9, 132.6, 129.6, 128.9, 128.2, 127.7, 127.5, 126.0, 125.7, 123.5, 122.2, 86.4, 21.4. HRMS (ESI) *m*/*z*: [M + H]^+^ Calcd for C_22_H_19_N_3_SH 358.1372; Found: 358.1374.

**1,3-Diphenyl-4-(o-tolylthio)-1** *H* **-pyrazol-5-amine (6d)**: Purified by column chromatography (hexane/ethyl acetate = 97:3); Yield: 0.125 g (50%); Brown solid, m.p: 89–90 °C; ^1^H NMR (CDCl_3_, 500 MHz) δ (ppm) = 7.83 (d, *J* = 10.0 Hz, 2H); 7.61 (d, *J* = 10.0 Hz, 2H); 7.43 (t, *J* = 5.0 Hz, 2H); 7.30 (t, *J* = 5.0 Hz, 1H); 7.26–7.21 (m, 3H); 7.07 (d, *J* = 5.0 Hz, 1H); 6.85 (d, *J* = 5.0, Hz, 1H); 4.19 (s, 2H); 2.36 (s, 3H). ^13^C­{^1^H} NMR (CDCl_3_, 126 MHz) δ (ppm) = 153.6, 149.4, 138.6, 136.8, 134.4, 132.5, 130.1, 129.6, 128.2, 128.2, 127.7, 127.5, 126.6, 124.7, 124.7, 123.9, 123.6, 85.5, 19.7. HRMS (ESI) *m*/*z*: [M + H]^+^ Calcd for C_22_H_19_N_3_SH 358.137245; Found: 358.1368.

**4-((4-Fluorophenyl)­thio)-1,3-diphenyl-1*H*-pyrazol-5-amine (6e)**: Purified by column chromatography (hexane/ethyl acetate = 97:3); Yield: 0.090 g (50%); Yellow solid, m.p: 103–104 °C; ^1^H NMR (CDCl_3_, 500 MHz) δ (ppm) = 7.93 (d, *J* = 10.0 Hz, 2H); 7.68 (d, *J* = 7.6 Hz, 2H); 7.52 (t, *J* = 7.9 Hz, 2H); 7.39 (t, *J* = 7.5 Hz, 1H); 7.36–7.31 (m, 3H); 7.20 (d, *J* = 8.6 Hz, 2H), 7.08 (d, *J* = 8.6 Hz, 2H), 4.32 (s, 2H). ^13^C­{^1^H} NMR (CDCl_3_, 126 MHz) δ (ppm) = 162,1, 160.2 (d, *J* = 245.0 Hz; C_arom_), 153.3, 149.6, 138.6, 133.2 (d, *J*_*C–F*_ = 3.0 Hz; C_arom_), 132.6, 129.8, 128.5, 128.4, 127.9, 127.6, 127.1, 127.0 (d, *J*_*C–F*_ = 8.0 Hz; C_arom_), 123.7, 116.3, 116.1 (d, *J*_*C–F*_ = 22.0 Hz; C_arom_), 86.8. HRMS (ESI) *m*/*z*: [M + H]^+^ Calcd for C_21_H_16_FN_3_SH 362.1121; Found: 362.1117.

**4-((4-Chlorophenyl)­thio)-1,3-diphenyl-1** *H* **-pyrazol-5-amine (6f)**:^30^ Purified by column chromatography (hexane/ethyl acetate = 97:3); Yield: 0.113 g (60%); Yellow solid, m.p: 111–113 °C; ^1^H NMR (CDCl_3_, 500 MHz) δ (ppm) = 7.94–7.91 (m, 2H); 7.68 (d, *J* = 7.6 Hz, 2H); 7.52 (t, *J* = 7.9 Hz, 2H); 7.39 (t, *J* = 7.5 Hz, 1H); 7.36–7.31 (m, 3H); 7.20 (d, *J* = 8.6 Hz, 2H), 7.08 (d, *J* = 8.6 Hz, 2H), 4.32 (s, 2H). ^13^C­{^1^H} NMR (CDCl_3_, 126 MHz) δ (ppm) = 153.3, 149.5, 138.5, 136.8, 132.4, 131.0, 129.7, 129.1, 128.4, 128.3, 127.8, 127.4, 126.4, 123.6, 85.8.

**3-Phenyl-4-(phenylselanyl)-1-(o-tolyl)-1** *H* **-pyrazol-5-amine (7a)**:^27^ Purified by column chromatography (hexane/ethyl acetate = 97:3); Yield: 0.060 g (30%); Yellow oil; ^1^H NMR (CDCl_3_, 500 MHz) δ (ppm) = 7.86–7.83 (m, 2H); 7.34 (d, *J* = 7.5 Hz, 1H); 7.30–7.27 (m, 2H); 7.25 (t, *J* = 7.1 Hz, 3H); 7.21 (d, *J* = 7.1 Hz, 1H); 7.19 (d, *J* = 7.2 Hz, 2H); 7.13 (t, *J* = 7.5 Hz, 2H); 7.06 (t, *J* = 7.2 Hz, 1H); 3.95 (s, 2H); 2.16 (s, 3H). ^13^C­{^1^H} NMR (CDCl_3_, 126 MHz) δ (ppm) = 153.3, 150.2, 136.7, 136.8, 133.4, 133.1, 131.5, 129.8, 128.1, 128.0, 127.8, 127.6, 127.0, 125.7, 80.3, 17.5.

**1-(4-Bromophenyl)-3-phenyl-4-(phenylselanyl)-1** *H* **-pyrazol-5-amine (7b)**: Purified by column chromatography (hexane/ethyl acetate = 97:3); Yield: 0.197 g (84%); Yellowish solid, m.p: 133–134 °C; ^1^H NMR (CDCl_3_, 500 MHz) δ (ppm) = 7.91 (d, *J* = 9.7 Hz, 2H); 7.65–7.60 (m, 4H); 7.37–7.32 (m, 3H); 7.27 (d, *J* = 9.8 Hz, 2H); 7.24–7.20 (m, 2H); 7.18–7.14 (m, 1H); 4.31 (s, 2H). ^13^C­{^1^H} NMR (CDCl_3_, 126 MHz) δ (ppm) = 154.0, 149.5, 137.8, 132.8, 132.8, 132.7, 129.3, 128.4, 128.2, 127.9, 127.8, 126.0, 125.0, 121.1, 83.2 HRMS (ESI) *m*/*z*: [M + H]^+^ Calcd for C_21_H_16_BrN_3_SeH 469.9773; Found: 469.9756.

**1-(3-Bromophenyl)-3-phenyl-4-(phenylselanyl)-1** *H* **-pyrazol-5-amine (7c)**: Purified by column chromatography (hexane/ethyl acetate = 97:3); Yield: 0.159 g (68%); Yellowish oil; ^1^H NMR (CDCl_3_, 500 MHz) δ (ppm) = 7.86 (m, 3H); 7.60 (d, *J* = 9.8 Hz, 1H); 7.44 (d, *J* = 8.8 Hz, 1H); 7.31 (d, *J* = 8.1 Hz, 1H); 7.29–7.25 (m, 3H); 7.21–7.16 (m, 3H); 7.15 (t, *J* = 7.5 Hz, 2H); 7.11–7.08 (m, 1H); 4.28 (s, 2H). ^13^C­{^1^H} NMR (CDCl_3_, 126 MHz) δ (ppm) = 154.1, 149.6, 139.9, 132.8, 132.7, 130.8, 130.6, 129.4, 128.4, 128.2, 127.9, 127.9, 126.6, 126.0, 123.2, 121.7, 83.2. HRMS (ESI) *m*/*z*: [M + H]^+^ Calcd for C_21_H_16_BrN_3_SeH 469.9763; Found: 469.9756.

**1-(3,4-Dichlorophenyl)-3-phenyl-4-(phenylselanyl)-1** *H* **-pyrazol-5-amine (7d)**: Purified by column chromatography (hexane/ethyl acetate = 97:3); Yield: 0.066 g (29%); Yellowish oil; ^1^H NMR (CDCl_3_, 500 MHz) δ (ppm) = 7.84 (d, *J* = 7.8 Hz, 3H); 7.55 (dd, *J* = 8.6, 2.3 Hz, 1H); 7.51 (d, *J* = 8.6 Hz, 1H); 7.31–7.26 (m, 3H); 7.19–7.14 (m, 4H); 7.10 (t, *J* = 7.0 Hz, 1H); 4.27 (s, 2H). ^13^C­{^1^H} NMR (CDCl_3_, 126 MHz) δ (ppm) = 154.2, 149.5, 138.0, 133.5, 132.6, 132.5, 131.3, 131.0, 129.3, 128.5, 128.2, 127.9, 127.8, 126.0, 125.0, 122.0, 83.8, 77.0. HRMS (ESI) *m*/*z*: [M + H]^+^ Calcd for C_21_H_15_Cl_2_N_3_SeH 459.9877; Found: 459.9874.

**1-(3,5-Dichlorophenyl)-3-phenyl-4-(phenylselanyl)-1** *H* **-pyrazol-5-amine (7f)**: Purified by column chromatography (hexane/ethyl acetate = 97:3); Yield: 0.156 g (68%); Yellowish solid, m.p: 122–123 °C; ^1^H NMR (CDCl_3_, 500 MHz) δ (ppm) = 7.82 (dd, *J* = 8.0, 1.6 Hz, 2H); 7.62 (d, *J* = 1.8 Hz, 2H); 7.30–7.25 (m, 4H); 7.18–7.12 (m, 4H); 7.10–7.07 (m, 1H); 4.29 (s, 2H). ^13^C­{^1^H} NMR (CDCl_3_, 126 MHz) δ (ppm) = 154.5, 149.6, 140.5, 135.9, 129.4, 128.6, 128.2, 128.0, 127.8, 127.2, 126.1, 114.3, 84.1. HRMS (ESI) *m*/*z*: [M + H]^+^ Calcd for C_21_H_15_Cl_2_N_3_SeH 459.9877; Found: 459.9875.

**1-(2,5-Dichlorophenyl)-3-phenyl-4-(phenylselanyl)-1** *H* **-pyrazol-5-amine (7g)**: Purified by column chromatography (hexane/ethyl acetate = 97:3); Yield: 0.041 g (18%); Orange solid, m.p: 158–159 °C; ^1^H NMR (CDCl_3_, 500 MHz) δ (ppm) = 7.81 (dd, *J* = 8.1, 1.5 Hz, 2H); 7.56 (d, *J* = 2.5 Hz, 1H); 7.41 (d, *J* = 8.7 Hz, 1H); 7.33 (dd, *J* = 8.7, 2.5 Hz, 1H); 7.28–7.23 (m, 3H); 7.19–7.13 (m, 5H); 7.08 (t, *J* = 7.1 Hz, 1H); 4.10 (s, 2H). ^13^C­{^1^H} NMR (CDCl_3_, 126 MHz) δ (ppm) = 154.6, 150.9, 136.7, 133.6, 132.9, 132.6, 131.3, 130.8, 130.2, 130.1, 129.3, 128.4, 128.1, 127.9, 127.7, 125.9, 81.9. HRMS (ESI) *m*/*z*: [M + H]^+^ Calcd for C_21_H_15_Cl_2_N_3_SeH 459.9877; Found: 459.9877.

**1-(2,4-Dichlorophenyl)-3-phenyl-4-(phenylselanyl)-1** *H* **-pyrazol-5-amine (7h)**:^28^ Purified by column chromatography (hexane/ethyl acetate = 97:3); Yield: 0.046 g (20%); Brown solid, m.p: 149–150 °C; ^1^H NMR (CDCl_3_, 500 MHz) δ (ppm) = 7.81 (dd, *J* = 8.1, 1.5 Hz, 2H); 7.52 (d, *J* = 2.2 Hz, 1H); 7.48 (d, *J* = 8.5 hz, 1H); 7.35 (dd, *J* = 8.5, 2.3 Hz, 1H); 7.29–7.24 (m, 3H); 7.19–7.13 (m, 4H); 7.08 (t, *J* = 7.1 Hz, 1H); 4.07 (s, 2H). ^13^C­{^1^H} NMR (CDCl_3_, 126 MHz) δ (ppm) = 154.5, 151.0, 136.2, 134.5, 133.0, 132.9, 132.7, 130.9, 130.4, 129.3, 128.5, 128.4, 128.2, 127.9, 127.7, 125.9, 81.8.

**3-Phenyl-4-(phenylselanyl)-1-(4-(trifluoromethyl)­phenyl)-1** *H* **-pyrazol-5-amine (7k)**: Purified by column chromatography (hexane/ethyl acetate = 97:3); Yield: 0.128 g (56%); Brown solid, m.p: 72–73 °C; ^1^H NMR (CDCl_3_, 500 MHz) δ (ppm) = 7.85 (dd, *J* = 8.0, 1.4 Hz, 2H); 7.82 (d, *J* = 8.4 Hz, 2H); 7.70 (d, *J* = 8.5 Hz, 2H); 7.31–7.26 (m, 3H); 7.19 (t, *J* = 5.9 Hz, 2H); 7.15 (t, *J* = 7.4 Hz, 2H); 7.09 (t, *J* = 7.1 Hz, 1H); 4.31 (s, 2H). ^13^C­{^1^H} NMR (CDCl_3_, 126 MHz) δ (ppm) = 170.0, 137.8, 131.5, 130.5, 129.9, 129.0, 128.8, 128.2, 127.8, 126.7, 123.1, 100.7. HRMS (ESI) *m*/*z*: [M + H]^+^ Calcd for C_22_H_16_F_3_N_3_SeH 460.0535; Found: 460.0543.

**3-Phenyl-4-(phenylthio)-1-(o-tolyl)-1** *H* **-pyrazol-5-amine (8a)**: Purified by column chromatography (hexane/ethyl acetate = 97:3); Yield: 0.080 g (45%), Orange oil; ^1^H NMR (CDCl_3_, 500 MHz) δ (ppm) = 7.94 (d, *J* = 8.2 Hz, 2H); 7.42 (d, *J* = 7.5 Hz, 1H); 7.39–7.36 (m, 2H); 7.35–7.28 (m, 2H); 7.24 (t, *J* = 7.7 Hz, 2H); 7.16 (d, *J* = 8.3 Hz, 2H); 7.10 (t, *J* = 7.2 Hz, 1H); 4.03 (s, 2H); 2.25 (s, 3H). ^13^C­{^1^H} NMR (CDCl_3_, 126 MHz) δ (ppm) = 153.0, 150.2, 138.5, 136.6, 136.5, 132.7, 131.5, 129.8, 129.0, 128.2, 128.1, 127.8, 127.5, 127.1, 125.0, 84.3, 17.6. HRMS (ESI) *m*/*z*: [M + H]^+^ Calcd for C_22_H_19_N_3_SH 358.1372; Found: 358.1375.

**1-(4-Bromophenyl)-3-phenyl-4-(phenylthio)-1** *H* **-pyrazol-5-amine (8b)**:^28^ Purified by column chromatography (hexane/ethyl acetate = 97:3); Yield: 0.073 g (35%); Brown solid, m.p: 128–129 °C; ^1^H NMR (CDCl_3_, 500 MHz) δ (ppm) = 7.85 (d, *J* = 9.7 Hz, 2H); 7.54 (d, *J* = 8.9 Hz, 2H); 7.50 (d, *J* = 8.9 Hz, 2H); 7.27–7.22 (m, 3H); 7.18–7.13 (m, 2H); 7.07 (dd, *J* = 8.4, 1.1 Hz, 2H); 7.03 (t, *J* = 7.3 Hz, 1H); 4.21 (s, 2H). ^13^C­{^1^H} NMR (CDCl_3_, 126 MHz) δ (ppm) = 153.7, 149.5, 137.8, 137.6, 132.7, 132.3, 129.1, 128.4, 128.3, 127.5, 125.2, 125.2, 124.8, 121.1, 87.0.

**1-(3-Bromophenyl)-3-phenyl-4-(phenylthio)-1** *H* **-pyrazol-5-amine (8c)**:^28^ Purified by column chromatography (hexane/ethyl acetate = 97:3); Yield: 0.122 g (58%), Orange oil; ^1^H NMR (CDCl_3_, 500 MHz) δ (ppm) = 7.86–7.83 (m, 3H); 7.57 (d, *J* = 10.9 Hz, 1H); 7.43 (d, *J* = 10.8 Hz, 1H); 7.29–7.23 (m, 4H); 7.19–7.14 (m, 2H); 7.07 (dd, *J* = 8.4, 1.1 Hz, 2H); 7.04 (t, *J* = 7.3 Hz, 1H); 4.26 (s, 2H). ^13^C­{^1^H} NMR (CDCl_3_, 126 MHz) δ (ppm) = 153.8, 149.6, 139.8, 137.8, 132.2, 130.8, 130.6, 129.1, 128.5, 128.3, 127.5, 126.5, 125.2, 125.2, 123.2, 121.6, 87.1.

**1-(3,4-Dichlorophenyl)-3-phenyl-4-(phenylthio)-1** *H* **-pyrazol-5-amine (8d)**: Purified by column chromatography (hexane/ethyl acetate = 97:3); Yield: 0.109 g (53%); Orange oil; ^1^H NMR (CDCl_3_, 500 MHz) δ (ppm) = 7.83 (dd, J = 8.0, 1.6 Hz, 2H); 7.78 (s, 1H); 7.49–7.43 (m, 2H); 7.27–7.22 (m, 3H); 7.17–7.12 (m, 2H); 7.06–7.01 (m, 4H); 4.25 (s, 2H). ^13^C­{^1^H} NMR (CDCl_3_, 126 MHz) δ (ppm) = 171.13, 154.13, 149.90, 138.32, 137.88, 133.84, 132.46, 131.56, 131.29, 129.28, 128.73, 128.45, 127.75, 125.66, 125.55, 125.16, 122.21, 88.30. HRMS (ESI) *m*/*z*: [M + H]^+^ Calcd for C_21_H_15_Cl_2_N_3_SH 412.0436; Found: 412.0434.

**1-(3,5-Dichlorophenyl)-3-phenyl-4-(phenylthio)-1*H*-pyrazol-5-amine (8f)**: Purified by column chromatography (hexane/ethyl acetate = 97:3); Yield: 0.057 g (60%); Orange oil; ^1^H NMR (CDCl_3_, 500 MHz) δ (ppm) = 7.92 (dd, J = 8.0, 1.6 Hz, 2H); 7.67 (s, 2H); 7.35–7.30 (m, 4H); 7.23–7.21 (m, 2H); 7.13–7.08 (m, 3H); 4.35 (s, 2H). ^13^C­{^1^H} NMR (CDCl_3_, 126 MHz) δ (ppm) = 154.12, 149.69, 140.35, 137.50, 135.91, 131.99, 129.10, 128.64, 128.30, 127.50, 127.24, 125.33, 125.25, 87.99. HRMS (ESI) *m*/*z*: [M + H]^+^ Calcd for C_21_H_15_Cl_2_N_3_S [M + H]^+^: 412.0436; Found: 412.0433.

**1-(2,5-Dichlorophenyl)-3-phenyl-4-(phenylthio)-1** *H* **-pyrazol-5-amine (8g)**: Purified by column chromatography (hexane/ethyl acetate = 97:3); Yield: 0.105 g (51%); Orange solid, m.p: 142–142 °C; ^1^H NMR (CDCl_3_, 500 MHz) δ (ppm) = 7.83 (d, *J* = 9.7 Hz, 2H); 7.56 (d, *J* = 2.4 Hz, 1H); 7.42 (d, *J* = 8.7 Hz, 1H); 7.34 (dd, *J* = 8.7, 2.5, Hz, 1H); 7.27–7.23 (m, 3H); 7.17 (t, *J* = 7.7 Hz, 2H); 7.08 (d, *J* = 9.6 Hz, 2H); 7.05–7.01 (m, 1H); 4.10 (s, 2H). ^13^C­{^1^H} NMR (CDCl_3_, 126 MHz) δ (ppm) = 154.3, 150.8, 137.8, 136.6, 133.7, 132.2, 131.3, 130.9, 130.2, 129.1, 128.5, 128.2, 127.5, 125.1, 125.0, 85.9. HRMS (ESI) *m*/*z*: [M + H]^+^ Calcd for C_21_H_15_Cl_2_N_3_SH 412.0436; Found: 412.0437.

**1-(2,4-Dichlorophenyl)-3-phenyl-4-(phenylthio)-1** *H* **-pyrazol-5-amine (8h)**: Purified by column chromatography (hexane/ethyl acetate = 97:3); Yield: 0.098 g (48%); Beige solid, m.p: 150–151 °C; ^1^H NMR (CDCl_3_, 500 MHz) δ (ppm) = 7.83 (dd, *J* = 8.0, 1.5 Hz, 2H); 7.52 (d, *J* = 2.2 Hz, 1H); 7.46 (d, *J* = 8.5 Hz, 1H); 7.34 (dd, *J* = 8.5, 2.2 Hz, 1H); 7.27–7.22 (m, 3H); 7.17 (t, *J* = 7.7 Hz, 2H); 7.08 (d, *J* = 7.4 Hz, 2H); 7.03 (t, *J* = 7.3 Hz, 1H); 4.06 (s, 2H). ^13^C­{^1^H} NMR (CDCl_3_, 126 MHz) δ (ppm) = 154.2, 150.9, 138.0, 136.3, 134.3, 132.8, 132.2, 130.8, 130.4, 129.1, 128.8, 128.4, 128.2, 127.5, 125.1, 125.0, 85.7. HRMS (ESI) *m*/*z*: [M + H]^+^ Calcd for C_21_H_15_Cl_2_N_3_SH 412.0436; Found: 412.0434.

**1-(4-Fluorophenyl)-3-phenyl-4-(phenylthio)-1** *H* **-pyrazol-5-amine (8k)**:^28^ Purified by column chromatography (hexane/ethyl acetate = 97:3); Yield: 0.090 g (21%); Brown oil;^1^H NMR (CDCl_3_, 500 MHz) δ (ppm) = 7.94 (d, *J* = 9.7 Hz, 2H); 7.66 (d, *J* = 9.6 Hz, 2H); 7.49 (t, *J* = 7.9 Hz, 2H); 7.38 (d, *J* = 8.5 Hz, 1H); 7.35–7.30 (m, 4H); 7.12–7.09 (m, 2H); 6.93 (t, *J* = 8.7 Hz, 2H); 4.32 (s, 2H). ^13^C­{^1^H} NMR (CDCl_3_, 126 MHz) δ (ppm) = 154.17, 149.75, 141.65, 137.66, 132.19, 129.43, 129.16 (q, *J* = 235.0 Hz; C_C–F_), 129.07, 128.65, 128.37, 127.55 (d, *J*_*C–F*_ = 5.3 Hz; C_arom_), 126.88 (q, *J J*_*C–F*_ = 3.6 Hz; C_arom_), 125.32 (d, *J*_*C–F*_ = 7.8 Hz; C_arom_), 123.00, 114.17, 87.87.

**1-(4-Bromophenyl)-4-((4-chlorophenyl)­selanyl)-3-phenyl-1** *H* **-pyrazol-5-amine (9a)**: Purified by column chromatography (hexane/ethyl acetate = 97:3); Yield: 0.075 g (40%); Orange oil; ^1^H NMR (CDCl_3_, 500 MHz) δ (ppm) = 7.87 (m, 2H), 7.61 (d, *J* = 8.8 Hz, 2H), 7.56 (d, *J* = 8.8 Hz, 2H), 7.36–7.30 (m, 2H), 7.16 (s, 4H), 4.30 (s, 2H). ^13^C­{^1^H} NMR (CDCl_3_, 126 MHz) δ (ppm) = 153.85, 149.45, 137.60, 132.69, 132.56, 132.04, 131.10, 129.39, 129.22, 128.46, 128.23, 127.73, 124.89, 121.20, 82.83. HRMS (ESI) *m*/*z*: [M + H]^+^ Calcd for C_21_H_15_BrClN_3_SeH 503.9371; Found: 503.9376.

**1-(4-Bromophenyl)-4-(naphthalen-1-ylselanyl)-3-phenyl-1** *H* **-pyrazol-5-amine (9b)**: Purified by column chromatography (hexane/ethyl acetate = 97:3); Yield: 0.060 g (30%); Orange oil; ^1^H NMR (CDCl_3_, 500 MHz) δ (ppm) = (d, *J* = 8.0 Hz, 1H), 7.93 – 7.88 (m, 2H), 7.84 (d, *J* = 7.6 Hz, 1H), 7.68 – 7.65 (m, 1H), 7.60 (d, *J* = 2.5 Hz, 3H), 7.56 – 7.48 (m, 2H), 7.30 (d, *J* = 6.8 Hz, 3H), 7.27 – 7.24 (m, 2H), 4.27 (s, 2H). ^13^C­{^1^H} NMR (CDCl_3_, 126 MHz) δ (ppm) = 154.36, 149.58, 137.76, 134.03, 132.69, 132.08, 131.30, 128.64, 128.41, 128.23, 127.78, 126.43, 126.29, 126.25, 125.09, 124.92, 121.12, 81.79. HRMS (ESI) *m*/*z*: [M + H]^+^ Calcd for C_25_H_18_BrN_3_SeH 519.9920; Found: 519.9920.

**General procedure for the synthesis of 1,3-diphenyl-1*H*-pyrazol-5-amine (10)**:^27^ In a sealed tube, benzoacetonitrile (**1**, 1.0 mmol, 0.145 g) and phenylhydrazine (**2a**, 1.0 mmol, 0.107 g) were dissolved in glacial acetic acid (10 mL). The reaction mixture was stirred at 50 °C for 1 h, and the progress was monitored by TLC. After completion, the reaction was cooled to room temperature and poured into water (20 mL). The mixture was extracted with ethyl acetate (3 × 15 mL). The combined organic layers were washed with water, dried over anhydrous Na_2_SO_4_, filtered, and concentrated under reduced pressure to afford the desired product as a crude solid/oil, which was used in the next step without further purification.

**1,3-Diphenyl-1*H*-pyrazol-5-amine (10)**:^27^ Purified by column chromatography (hexane/ethyl acetate = 95:5); Yield: 0.230 g (90%); Orange oil; ^1^H NMR (CDCl_3_, 500 MHz) δ (ppm) = 7.82 −7.78 (m, 2H), 7.63 −7.59 (m, 2H), 7.47 (t, *J* = 7.9 Hz, 2H), 7.39 – 7.26 (m, 6H), 5.93 (s, 1H), 3.84 (s, 2H). ^13^C­{^1^H} NMR (CDCl_3_, 126 MHz) δ (ppm) = 187.11, 151.46, 145.85, 138.65, 129.48, 129.11, 128.48, 127.80, 127.41, 125.60, 124.10, 113.78, 88.12.

**General procedure for the synthesis of 1,3-Diphenyl-4-(phenylselanyl)-5-(1H-pyrrol-1-yl)-1** *H* **-pyrazole (11)**: In a sealed tube, compound **4a** (1.0 mmol, 0.389 g) and 2,5-dimethoxytetrahydrofuran (DMTHF, 1 equiv., 1.0 mmol, 0.132 g) were dissolved in acetic acid (3.0 mL). The reaction mixture was stirred at 120 °C until completion (monitored by TLC). After cooling to room temperature, the mixture was diluted with water and carefully neutralized with a saturated aqueous sodium bicarbonate solution. The aqueous phase was extracted with ethyl acetate (3 × 10 mL). The combined organic layers were washed with water, dried over anhydrous sodium sulfate, filtered, and concentrated under reduced pressure. The crude product was purified by column chromatography on silica gel using hexane/ethyl acetate (9:1, v/v) as the eluent to afford the desired product.

**Caution**: Reactions conducted at elevated temperatures were performed using robust glassware, positioned behind an appropriate safety shield.

**1,3-Diphenyl-4-(phenylselanyl)-5-(1*H*-pyrrol-1-yl)-1** *H* **-pyrazole (11)**: Purified by column chromatography (hexane/ethyl acetate = 97:3); Yield: 0.106 g (80%); Yellowish solid, m.p: 201–202 °C; ^1^H NMR (CDCl_3_, 500 MHz) δ (ppm) = 7.89 (d, *J* = 8.4 Hz, 2H), 7.43 (t, *J* = 7.5 Hz, 3H), 7.35 (d, *J* = 21.6 Hz, 4H), 7.29 (d, *J* = 7.2 Hz, 1H), 7.22 (d, *J* = 8.6 Hz, 1H), 6.70 (dd, *J* = 4.8, 2.7 Hz, 3H), 6.28 (t, *J* = 2.1 Hz, 2H).^13^C­{^1^H} NMR (CDCl_3_, 126 MHz) δ (ppm) = 151.36, 138.37, 132.65, 129.09, 128.70, 128.35, 127.54, 125.64, 122.97, 122.13, 110.61, 100.02. HRMS (ESI) *m*/*z*: [M + H]^+^ Calcd for C_25_H_19_N_3_SeH 442.0818; Found: 442.0824.

## Supplementary Material



## Data Availability

The data underlying this study are available in the published article and its Supporting Information. Copies of (^1^H and ^13^C) NMR spectra of the compounds.

## Notes

Reaction setup and photochemical reaction under blue LED (Figure S1); ^1^H NMR and ^13^C­{^1^H} spectra of compounds 4a–j (Figure S2–17); ^1^H and ^13^C­{^1^H} NMR spectra of compounds 6a–f (Figure S18–29); ^1^H and ^13^C­{^1^H} NMR spectra of compounds 7a–c (Figure S30–35); ^1^H and ^13^C­{^1^H} NMR spectra of compound 7d (Figure S36, 37); ^1^H and ^13^C­{^1^H} NMR spectra of compound 7f (Figure S38, 39); ^1^H and ^13^C­{^1^H} NMR spectra of compound 7g (Figure S40, 41); ^1^H and ^13^C­{^1^H} NMR spectra of compound 7h (Figure S42, 43); ^1^H and 13C­{1H} NMR spectra of compound 7k (Figure S44, 45); ^1^H and ^13^C­{^1^H} NMR spectra of compound 8a (Figure S46, 47); ^1^H and ^13^C­{^1^H} NMR spectra of compound 8b (Figure S48, 49); ^1^H and ^13^C­{1H} NMR spectra of compound 8c (Figure S50, 51); ^1^H and ^13^C­{1H} NMR spectra of compound 8d (Figure S52, 53); ^1^H and ^13^C­{^1^H} NMR spectra of compound 8f (Figure S54, 55); ^1^H and ^13^C­{^1^H} NMR spectra of compound 8g (Figure S56, 57); ^1^H and ^13^C­{^1^H} NMR spectra of compound 8h (Figure S58, 59); ^1^H and ^13^C­{^1^H} NMR spectra of compound 8k (Figure S60, 61); ^1^H and ^13^C­{^1^H} NMR spectra of compound 9a (Figure S62, 63); ^1^H and ^13^C­{^1^H} NMR spectra of compound 9b (Figure S64, 65); ^1^H and ^13^C­{^1^H} NMR spectra of compound 10 (Figure S66, 67); and ^1^H and ^13^C­{^1^H} NMR spectra of compound 11 (Figure S68, 69) (PD)
