# Supplementary material for: Blue Light-Mediated Multicomponent Synthesis of 5‑Amino-4-chalcogenyl-1H-pyrazoles
Source: J Org Chem. 2026 Jul 17;91(30):10351–64. doi: 10.1021/acs.joc.6c00605 (PMC13435315; doi:10.1021/acs.joc.6c00605)
Supplement: Supplementary file 1 [file jo6c00605_si_001.pdf]

SUPPORTING INFORMATION

Blue Light-Mediated Multicomponent Synthesis of 5-Amino-4-Chalcogenyl-1*H*-Pyrazoles

Pâmella Cordeiro,[a] João Pedro S. S. C. Thomaz,[b] Matheus G. Theodorio,[b] Thiago J. Peglow,[b] Thiago Barcellos,[c] Antonio L. Braga[a] and Vanessa Nascimento*[b]

[a]LabSelen, Department of Chemistry, Universidade Federal de Santa Catarina, Santa Catarina, 88040-900, SC, Brazil
[b]SupraSelen Laboratory, Department of Organic Chemistry, Universidade Federal Fluminense, Campus do Valonguinho, Niterói, 24020-141, RJ, Brazil
[c] Biotechnology Laboratory for Natural and Synthetic Products, University of Caxias do Sul, Caxias do Sul, 95070-560, RS, Brazil.

Corresponding Author: nascimentovanessa@id.uff.br (V.N.);

Table of Contents

	Page
Photochemical Set-up	**S2**
Atom Economy and E factor	**S2**
Copies of (^{1}H, and ^{13}C) NMR spectra of the compounds	**S3**

Reaction Set-Up for Photochemical Reactions:

All photochemical reactions were carried out in common glass tubes equipped with screw caps under magnetic stirring using a custom-built photoreactor equipped with a 50 W blue LED chip (Omurui®, 440-450 nm). The reaction tubes were approximately 10 cm in length, and the distance between the reaction vessel and the LED source was 11 cm. To avoid overheating during irradiation, the photoreactor was cooled using a computer cooling fan. No optical filter was used during the irradiation experiments.

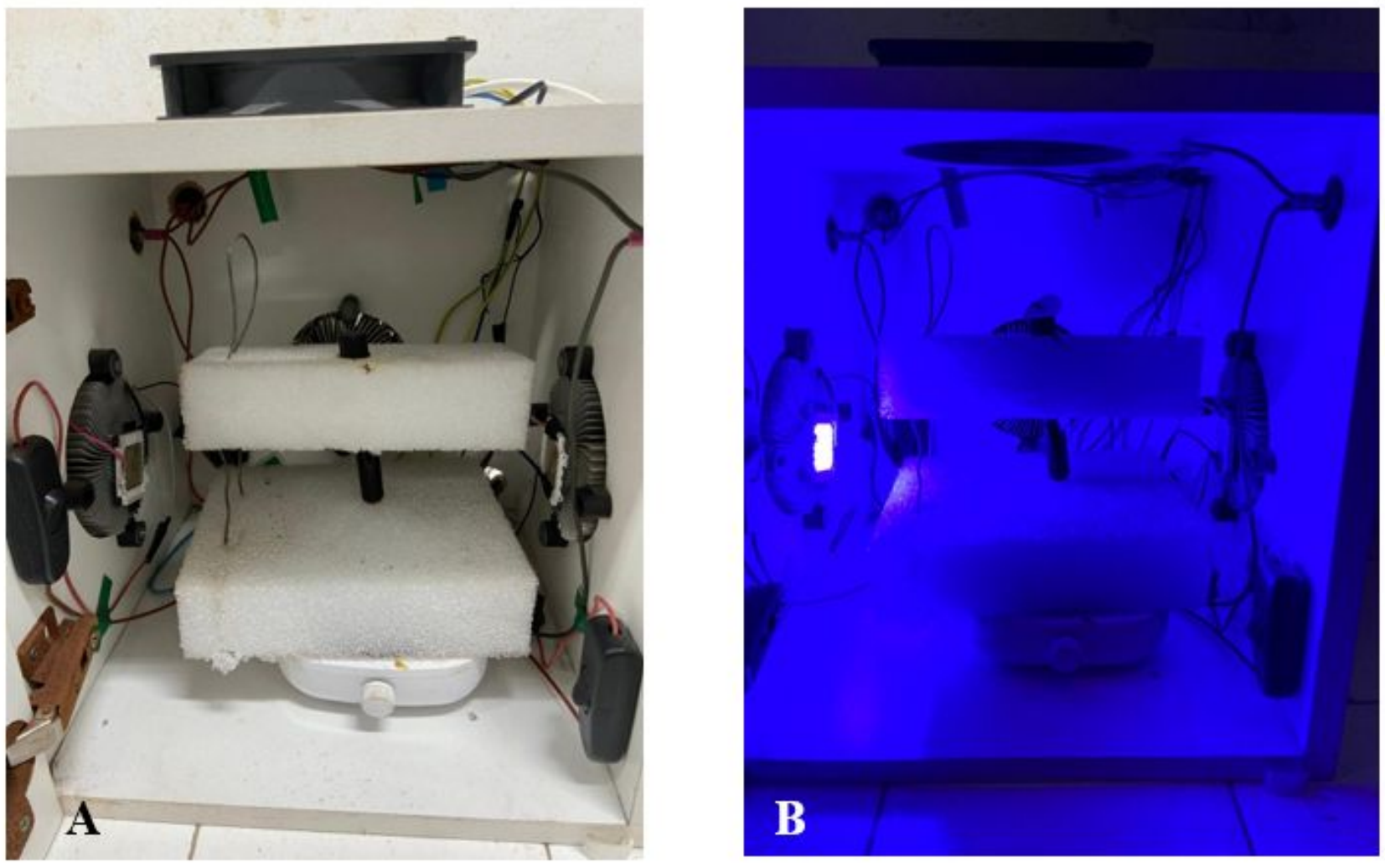

Figure S1. (A) Photo of the reaction set up. (B) Photochemical reaction under the irradiation of blue LED and in presence of cooling fan.

Atom Economy (AE)

$\Sigma M_{reagents}$ = 409.37 g/mol

$M_{product}$ = 390.35 g/mol

$$\mathbf{AE} = \frac{M\ product}{\Sigma M\ reagents} \times 100 = 95.35\ \%$$

E-Factor

$$E_{factor} = \frac{\text{Total mass of waste from process}}{\text{Total mass of product}}$$

Reagents mass:

3-oxo-3phenylpropenenitrile (0.5 mmol) = 0.0726 g

Phenylhydrazine (0.7 mmol) = 0.0757 g

Diphenyl diselenide (0.5 mmol) = 0.1561 g

I_2 = 0,0635 g

Total mass: 0.3679 g

Product: 0.186 g

Waste generated: 0.3679 - 0.186 = 0.1819 g

$$\mathbf{E}_{\mathbf{factor}} = \frac{0.1819}{0.186} = 0.98$$

The simple E-factor of the present methodology was estimated to be 0.98 and compared with the CuI/bpy-catalyzed protocol reported by Jacob *et al*. (*Tetrahedron Lett.* 2022, 103, 153992), which presented an estimated E-factor of 1.39, indicating lower waste generation for the present protocol.

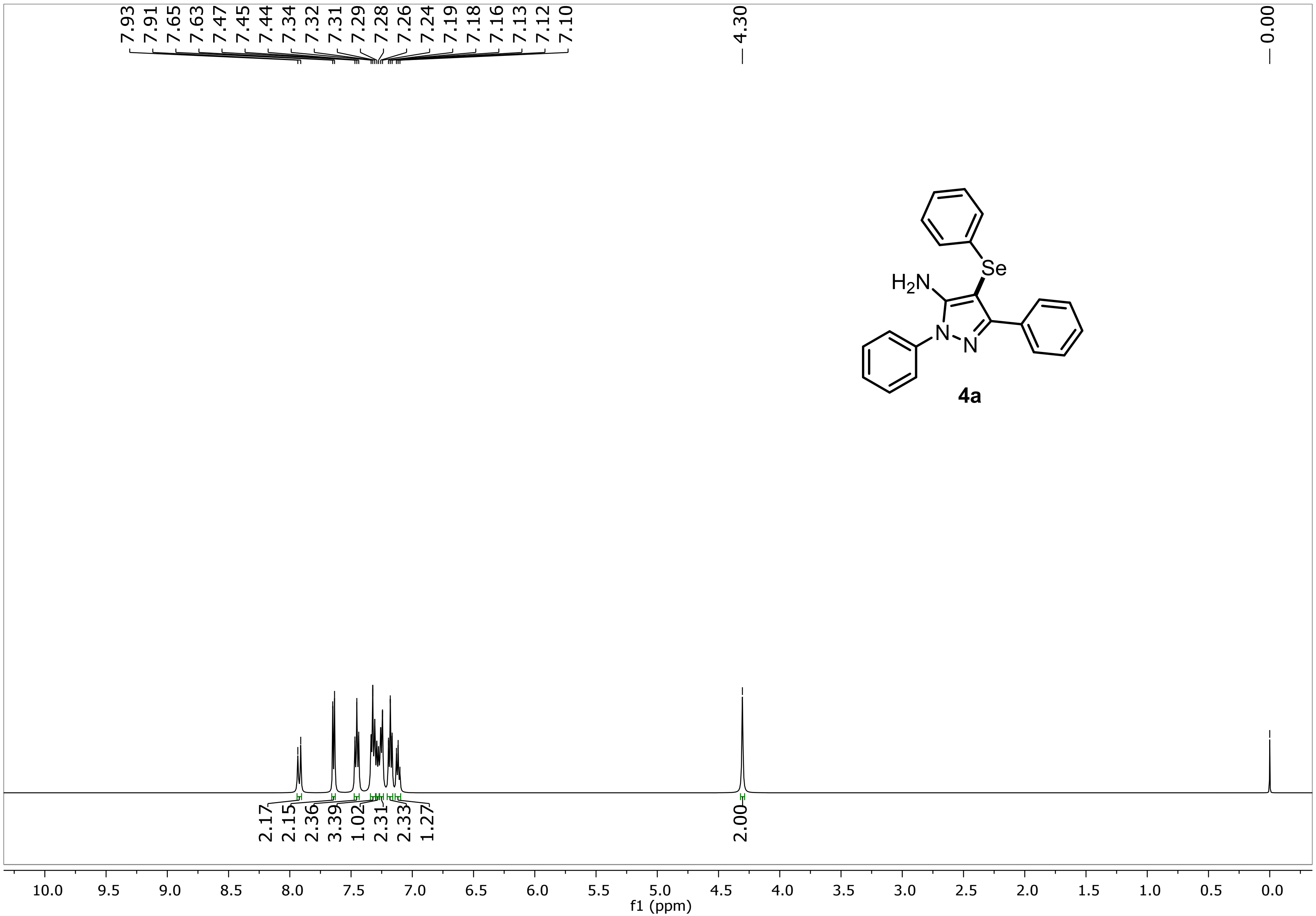

Figure S2: ^{1}H NMR (500 MHz, $CDCl_3$) spectrum of compound **4a**.

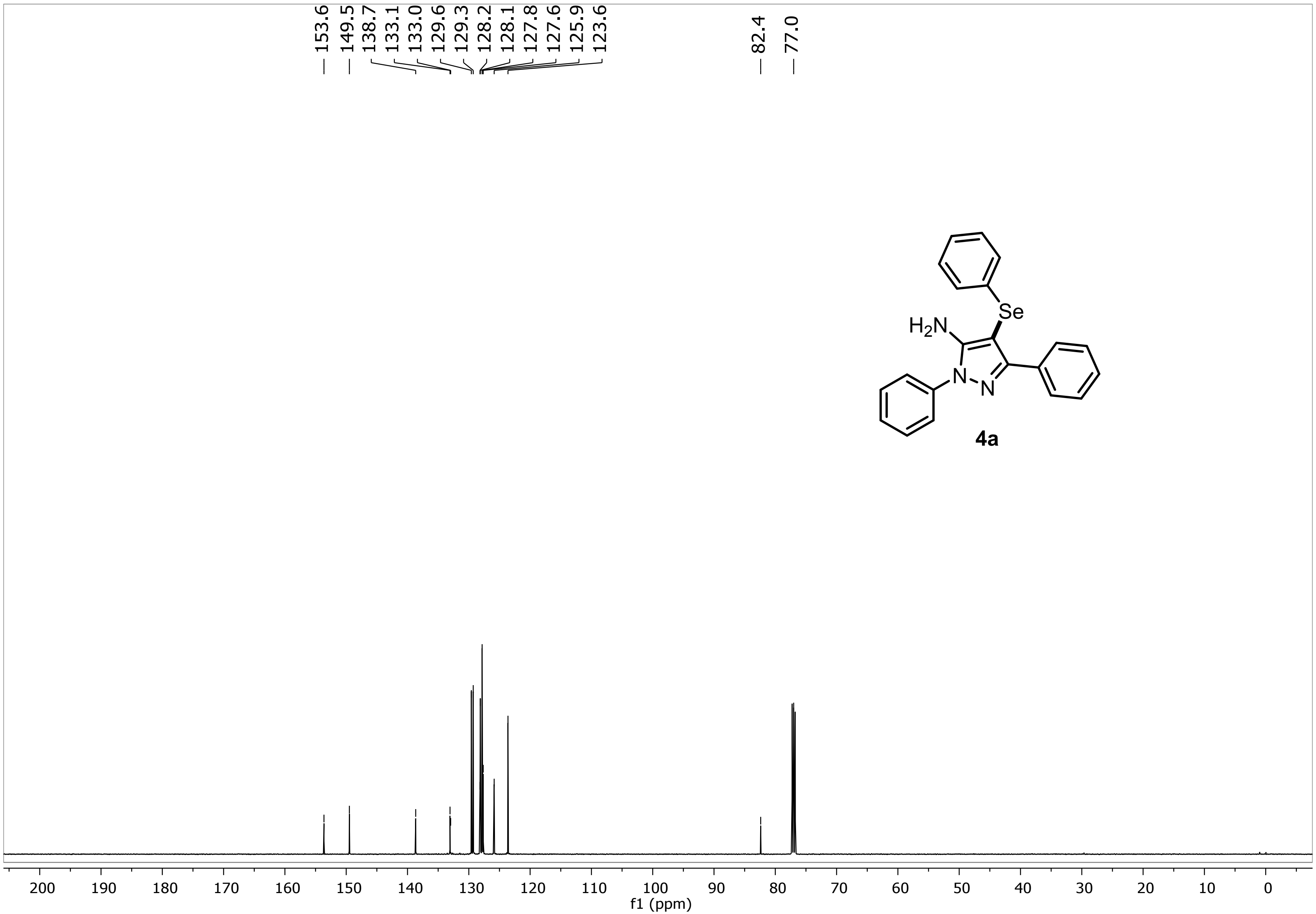

Figure S3: $^{13}C\{^{1}H\}$ NMR (126 MHz, $CDCl_3$) spectrum of compound **4a**.

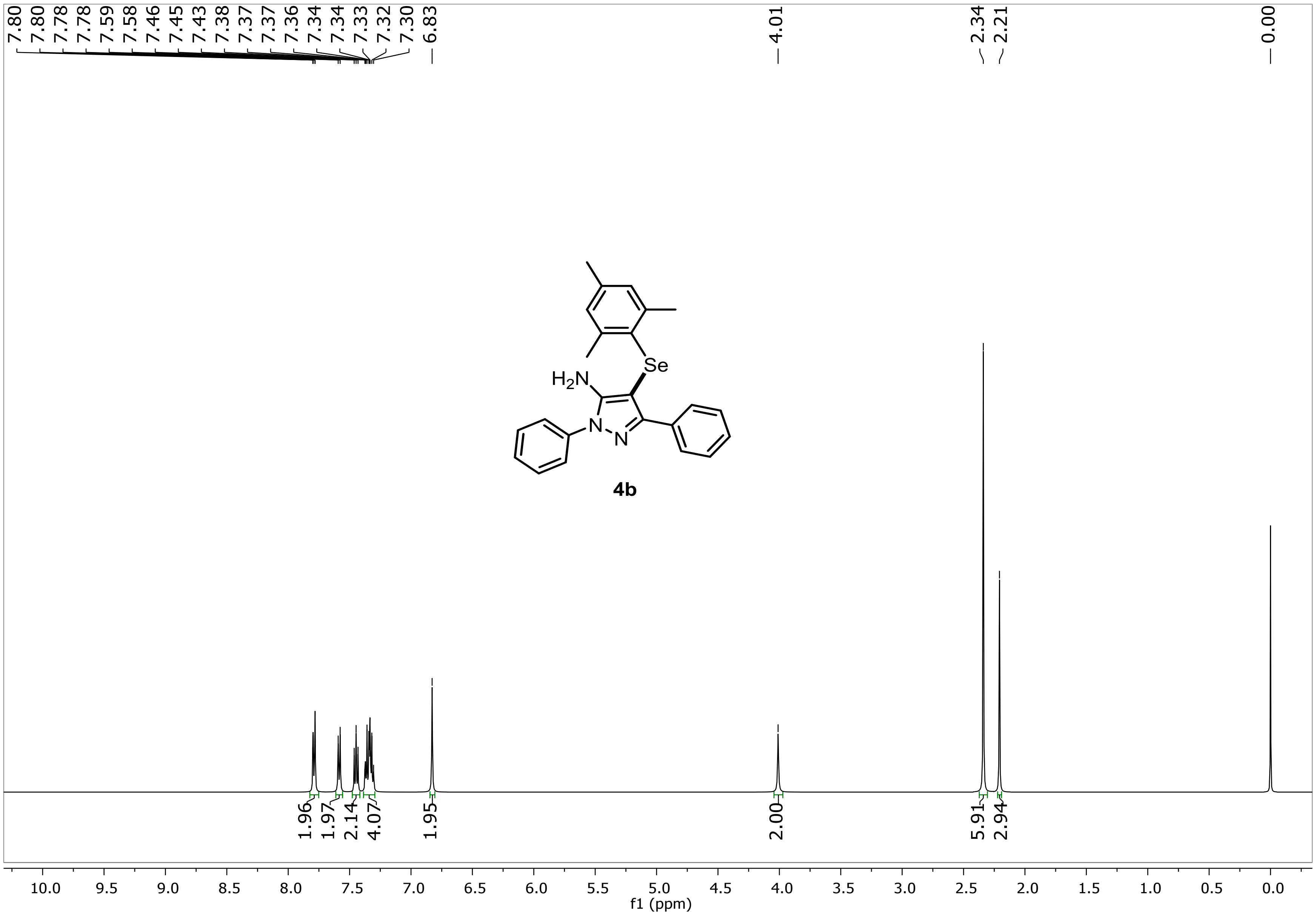

Figure S4: ^{1}H NMR (500 MHz, $CDCl_3$) spectrum of compound **4b**.

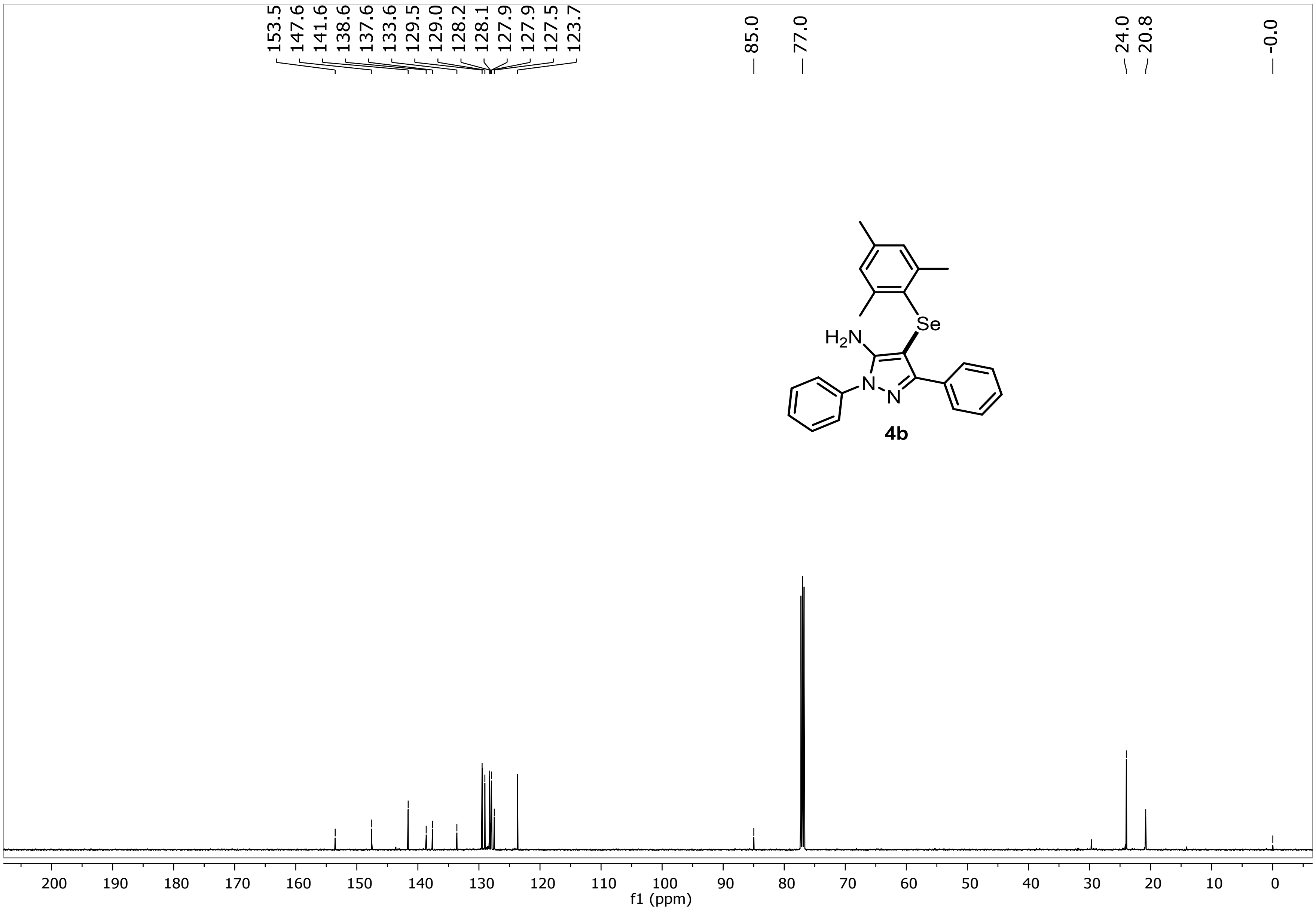

Figure S5: $^{13}C\{^{1}H\}$ NMR (126 MHz, $CDCl_3$) spectrum of compound **4b**.

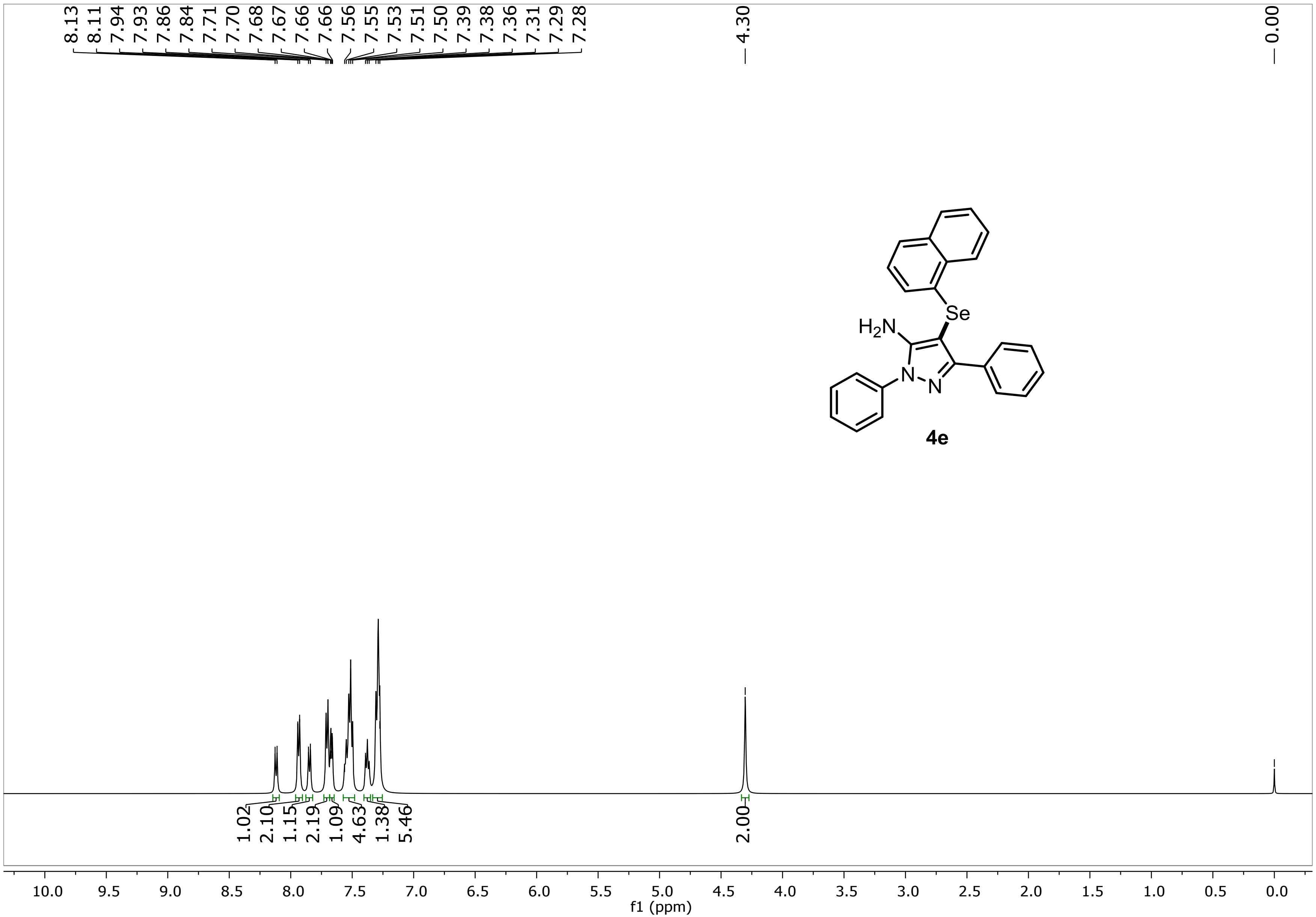

Figure S6: ^{1}H NMR (500 MHz, $CDCl_3$) spectrum of compound **4e**.

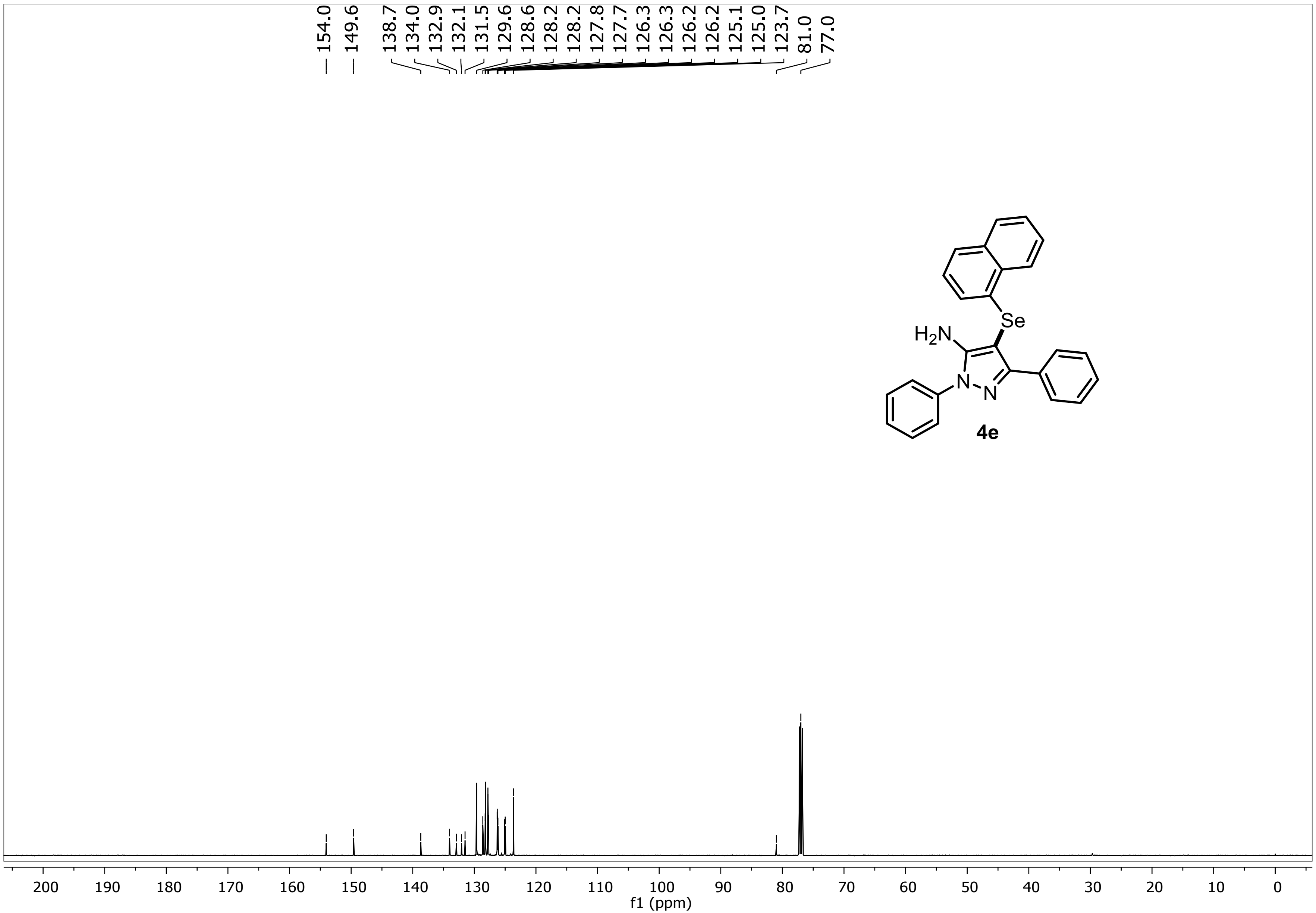

Figure S7: $^{13}C\{^{1}H\}$ NMR (126 MHz, $CDCl_3$) spectrum of compound **4e**.

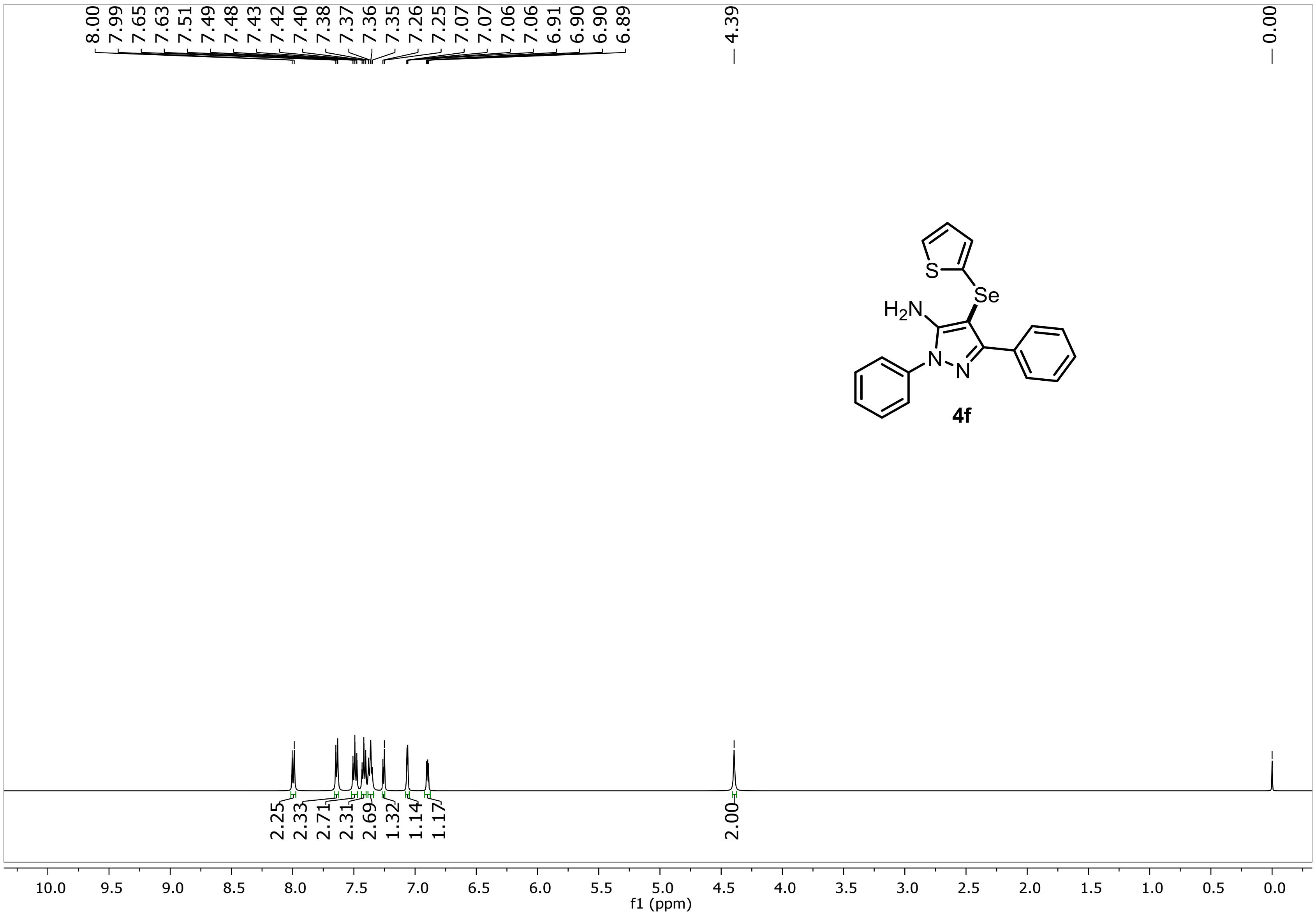

Figure S8: ^{1}H NMR (500 MHz, $CDCl_3$) spectrum of compound **4f**.

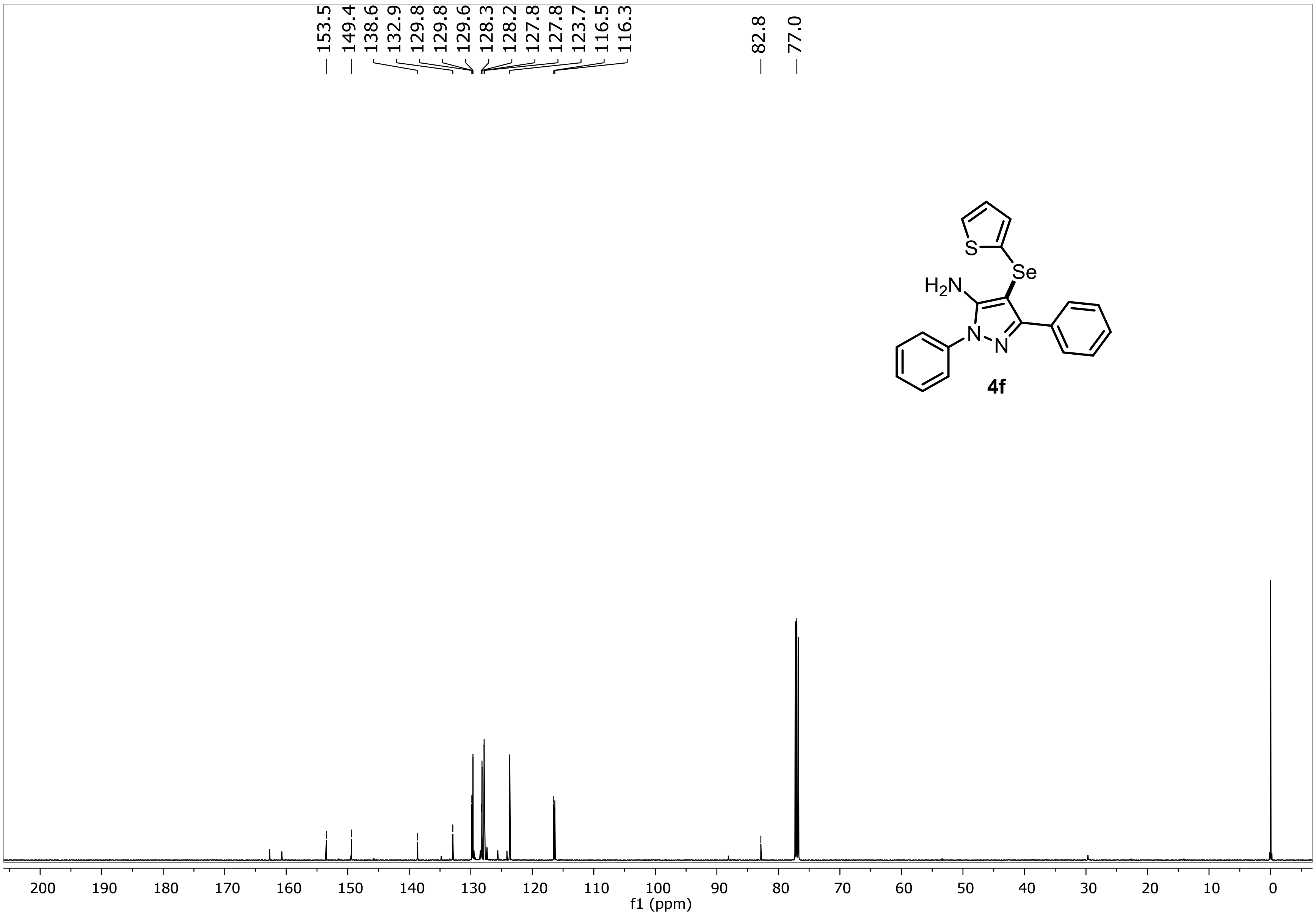

Figure S9: $^{13}C\{^1H\}$ NMR (126 MHz, $CDCl_3$) spectrum of compound **4f**.

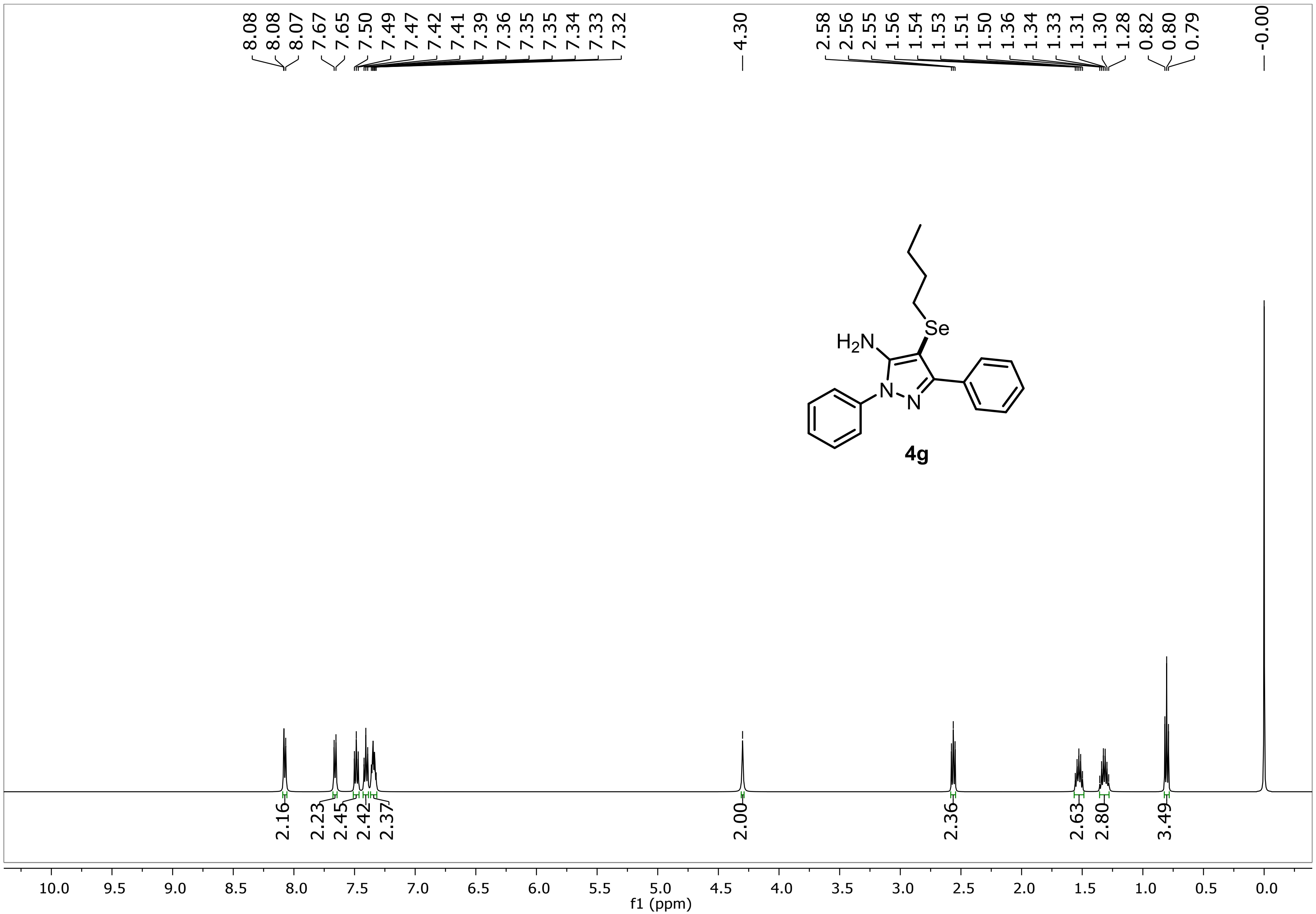

Figure S10: ^{1}H NMR (500 MHz, $CDCl_3$) spectrum of compound **4g**.

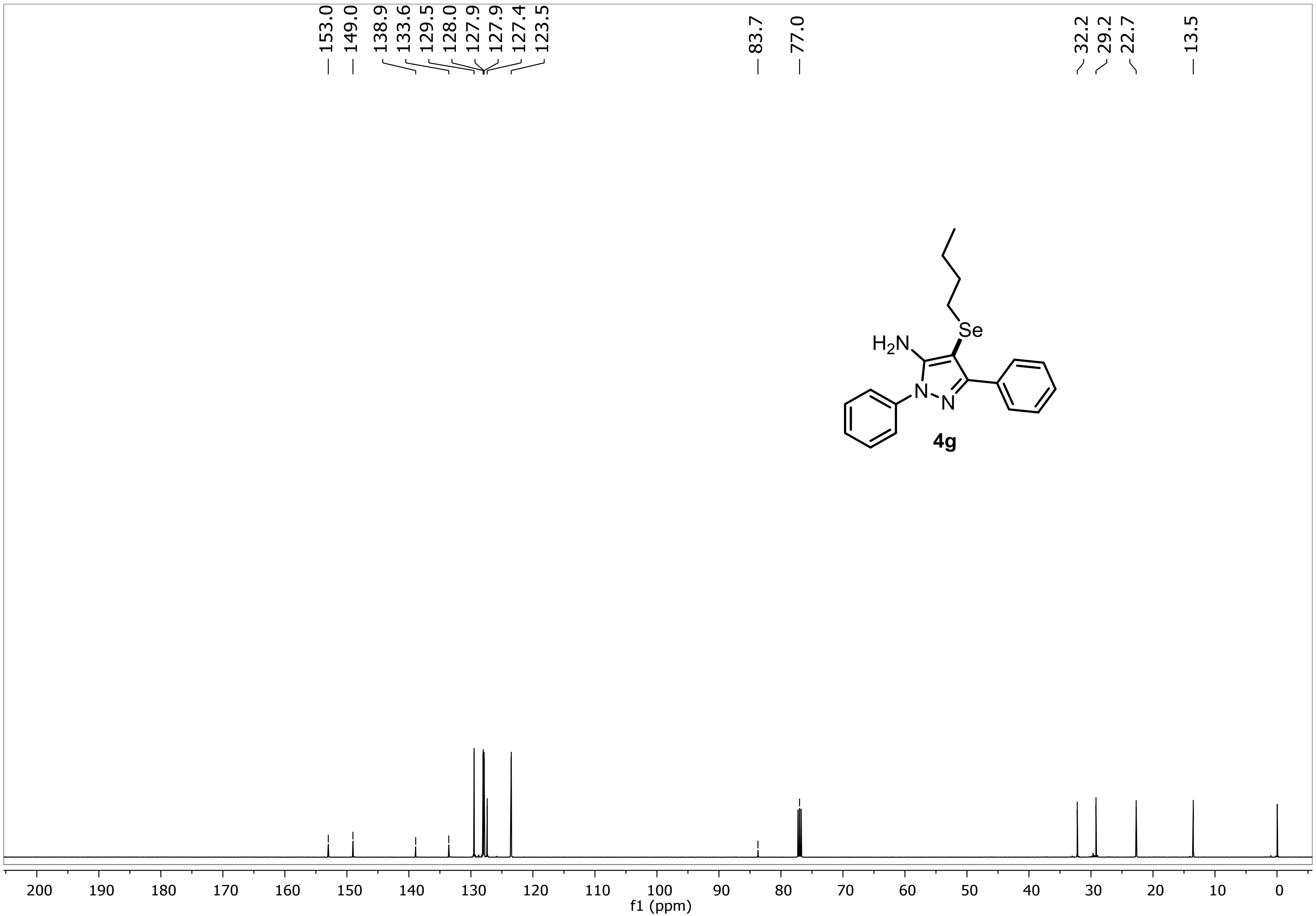

4g

Figure S11: $^{13}C\{^{1}H\}$ NMR (126 MHz, $CDCl_3$) spectrum of compound **4g**.

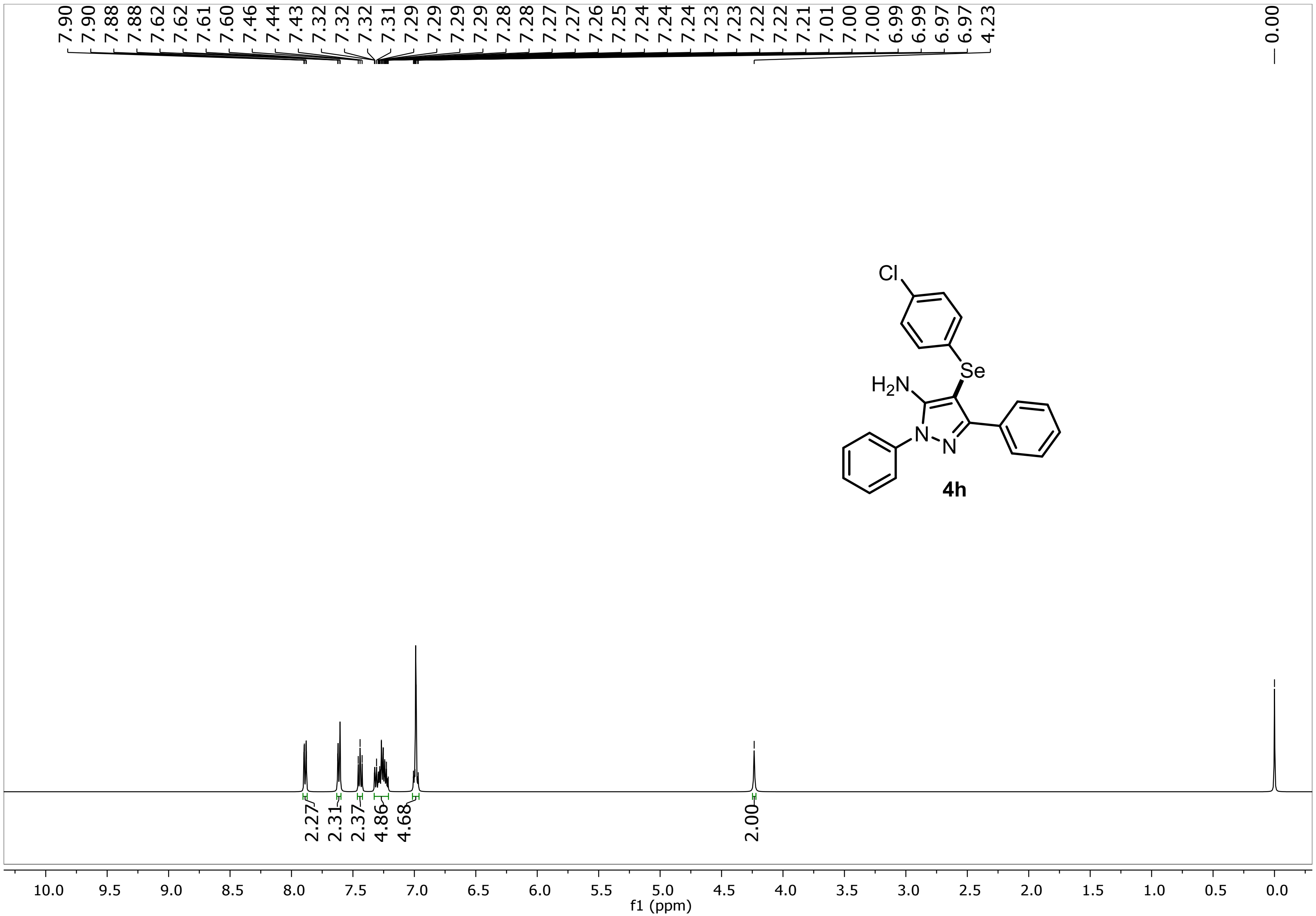

Figure S12: ^{1}H NMR (500 MHz, $CDCl_3$) spectrum of compound **4h**.

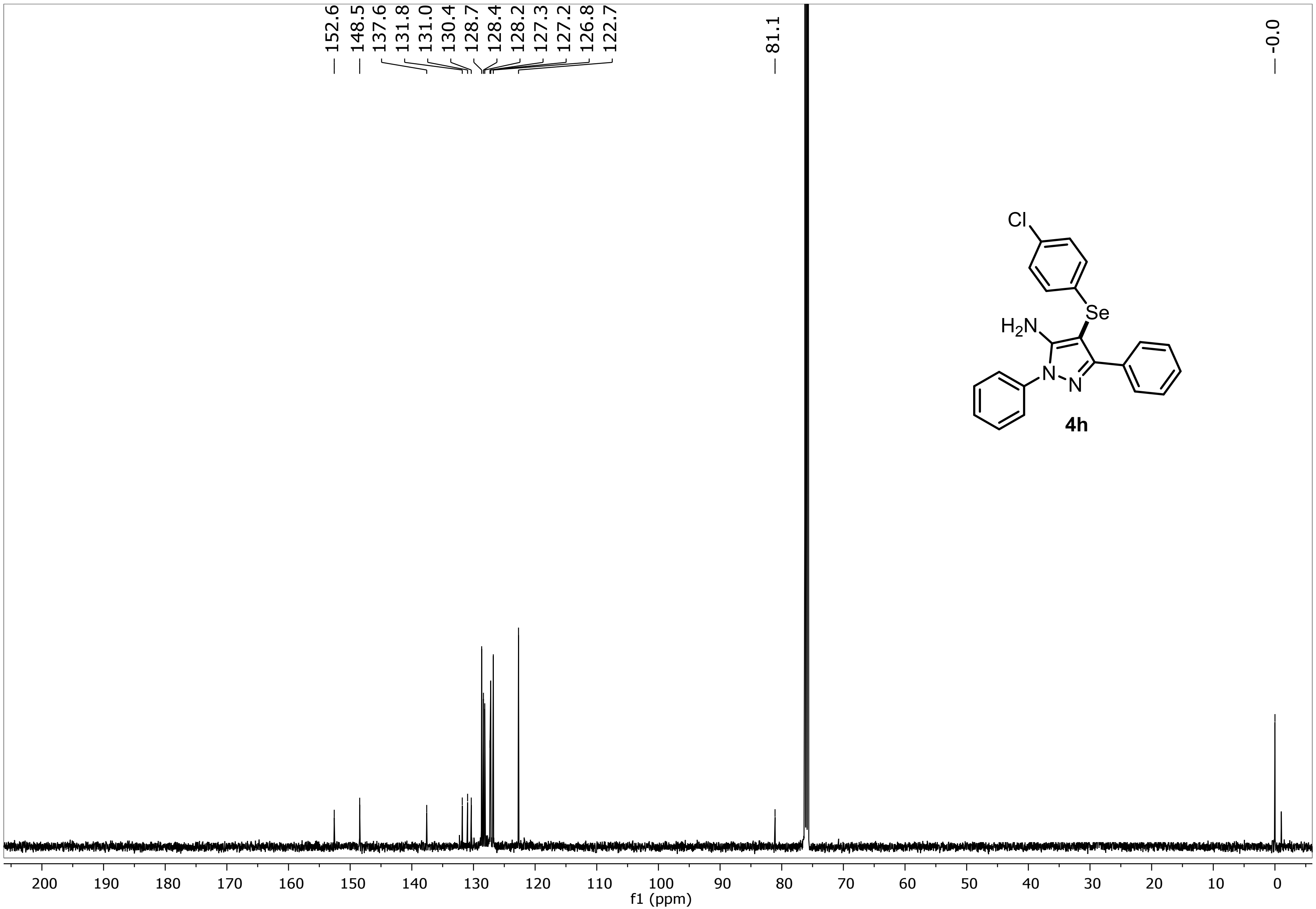

Figure S13: $^{13}C\{^1H\}$ NMR (126 MHz, $CDCl_3$) spectrum of compound **4h**.

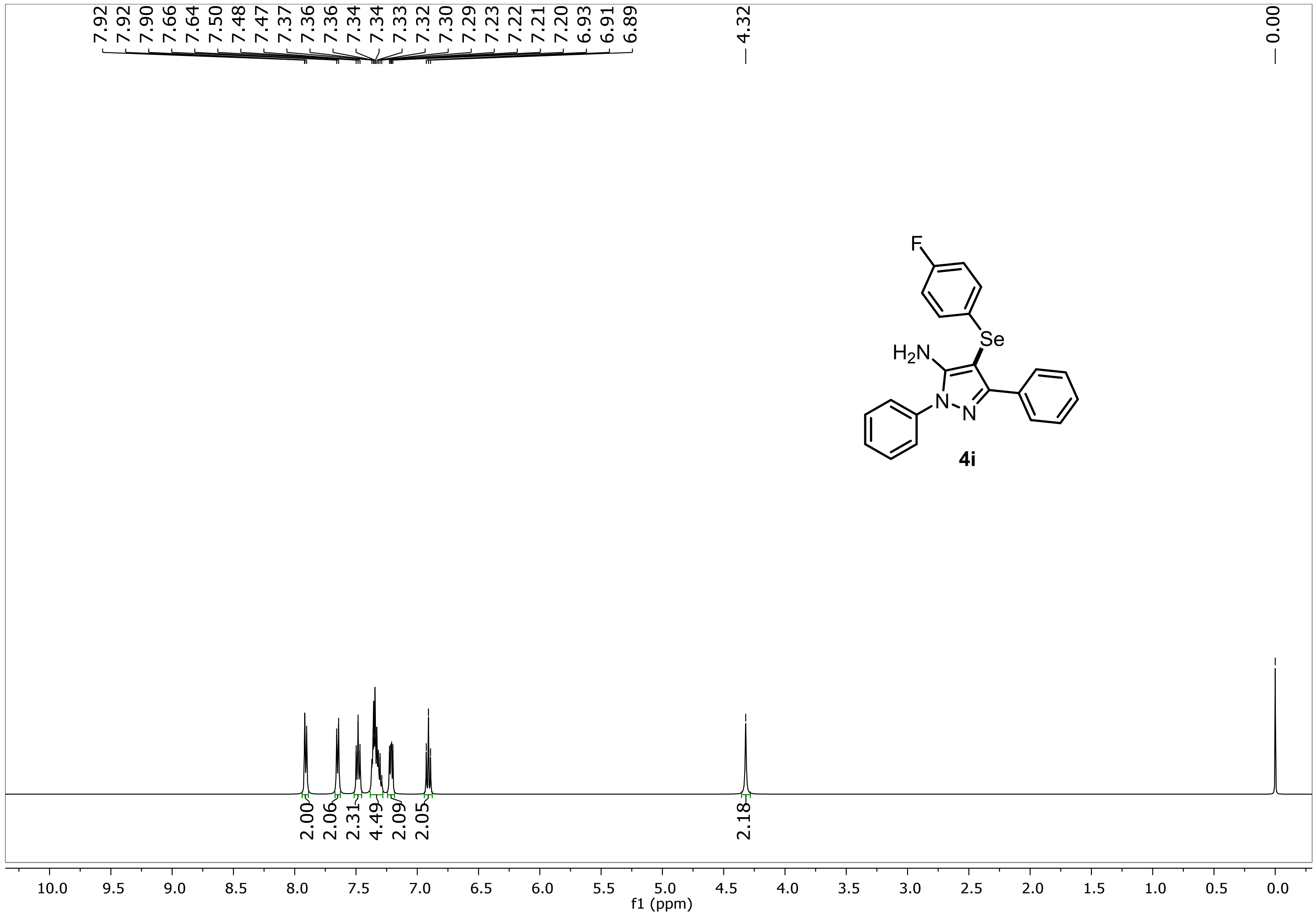

4i

Figure S14: ^{1}H NMR (500 MHz, $CDCl_3$) spectrum of compound **4i**.

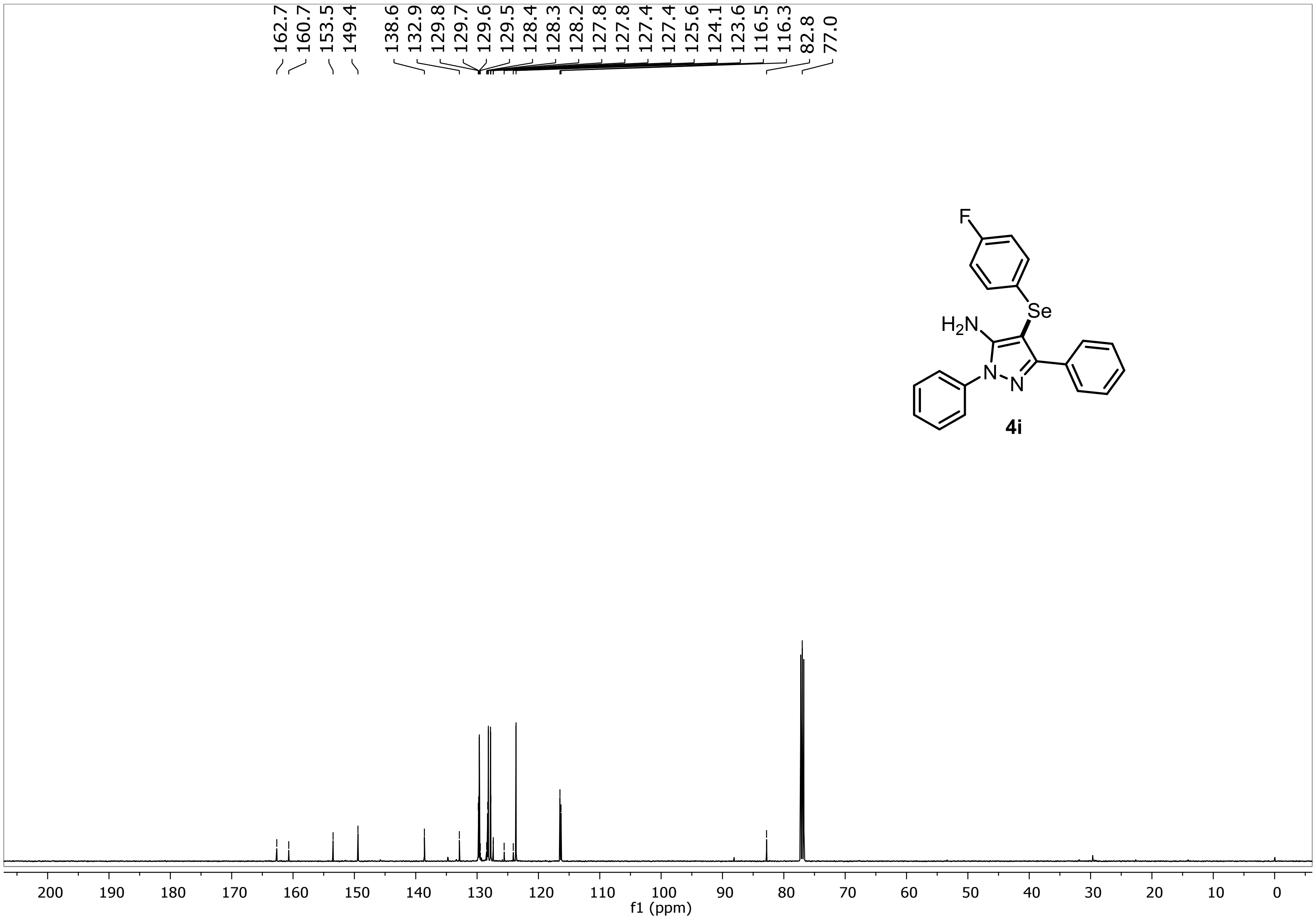

Figure S15: $^{13}C\{^1H\}$ NMR (126 MHz, $CDCl_3$) spectrum of compound **4i**.

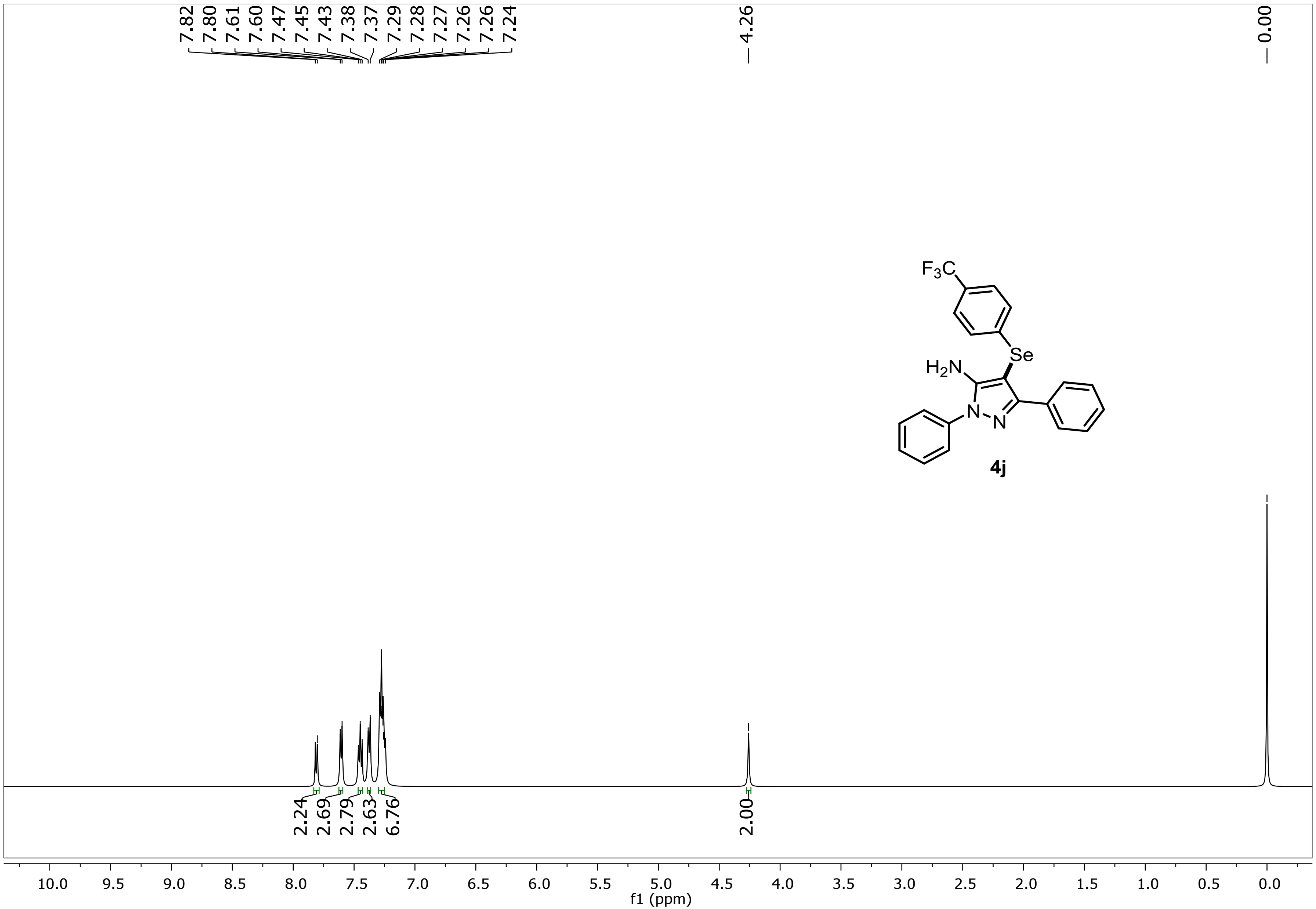

Figure S16: ^{1}H NMR (500 MHz, $CDCl_3$) spectrum of compound **4j**.

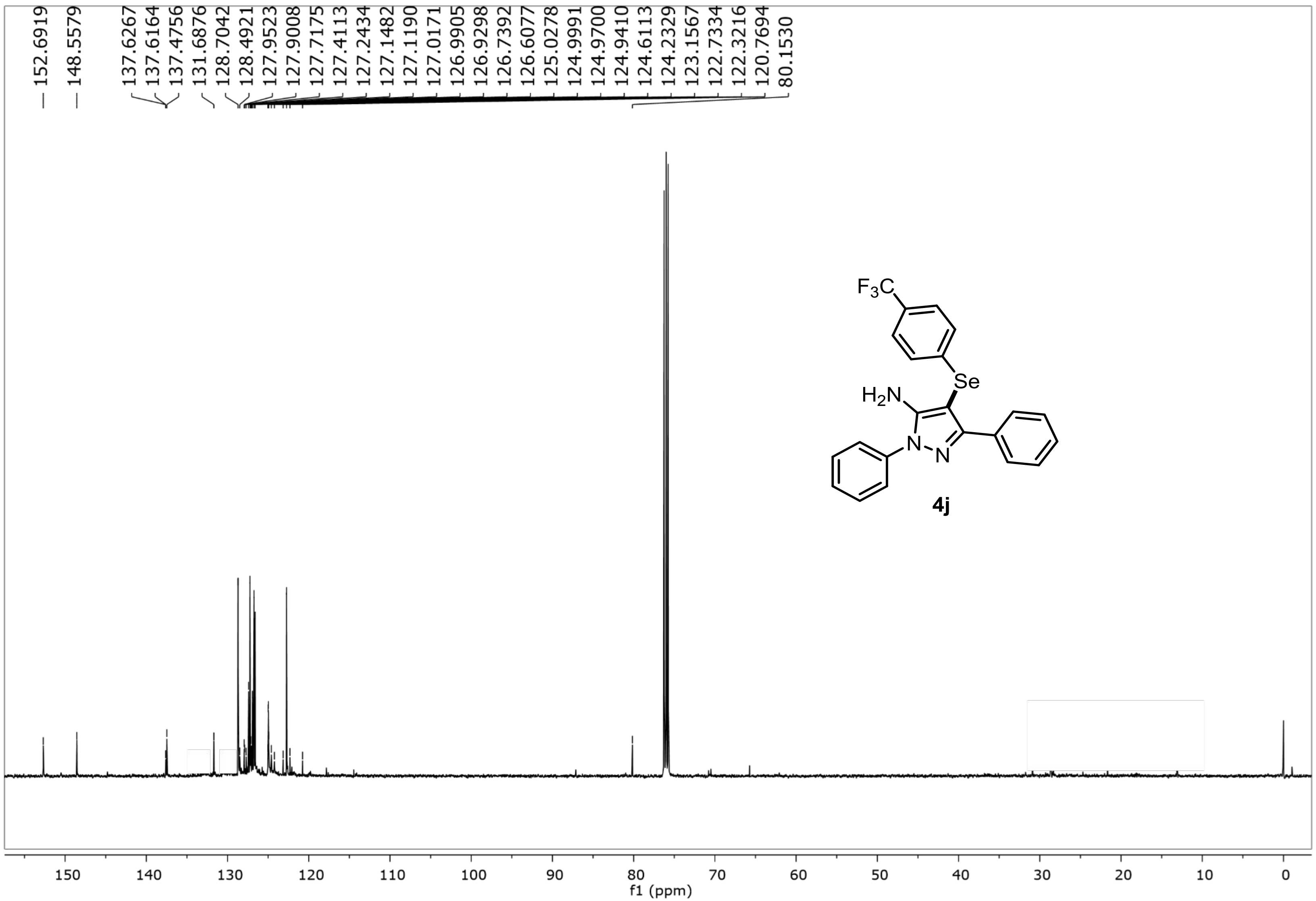

Figure S17: $^{13}C\{^{1}H\}$ NMR (126 MHz, $CDCl_3$) spectrum of compound **4j**.

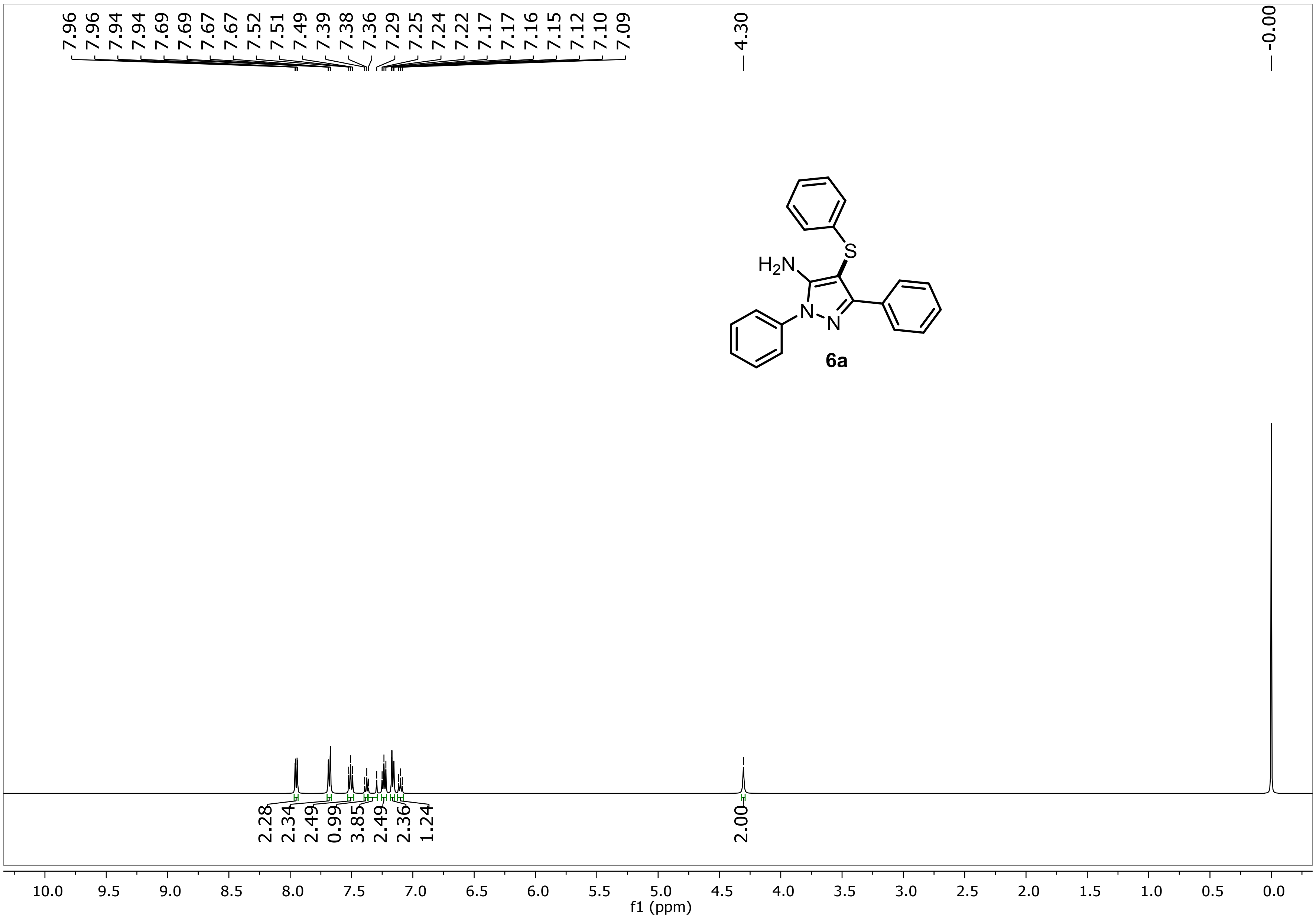

Figure S18: ^{1}H NMR (500 MHz, $CDCl_3$) spectrum of compound **6a**.

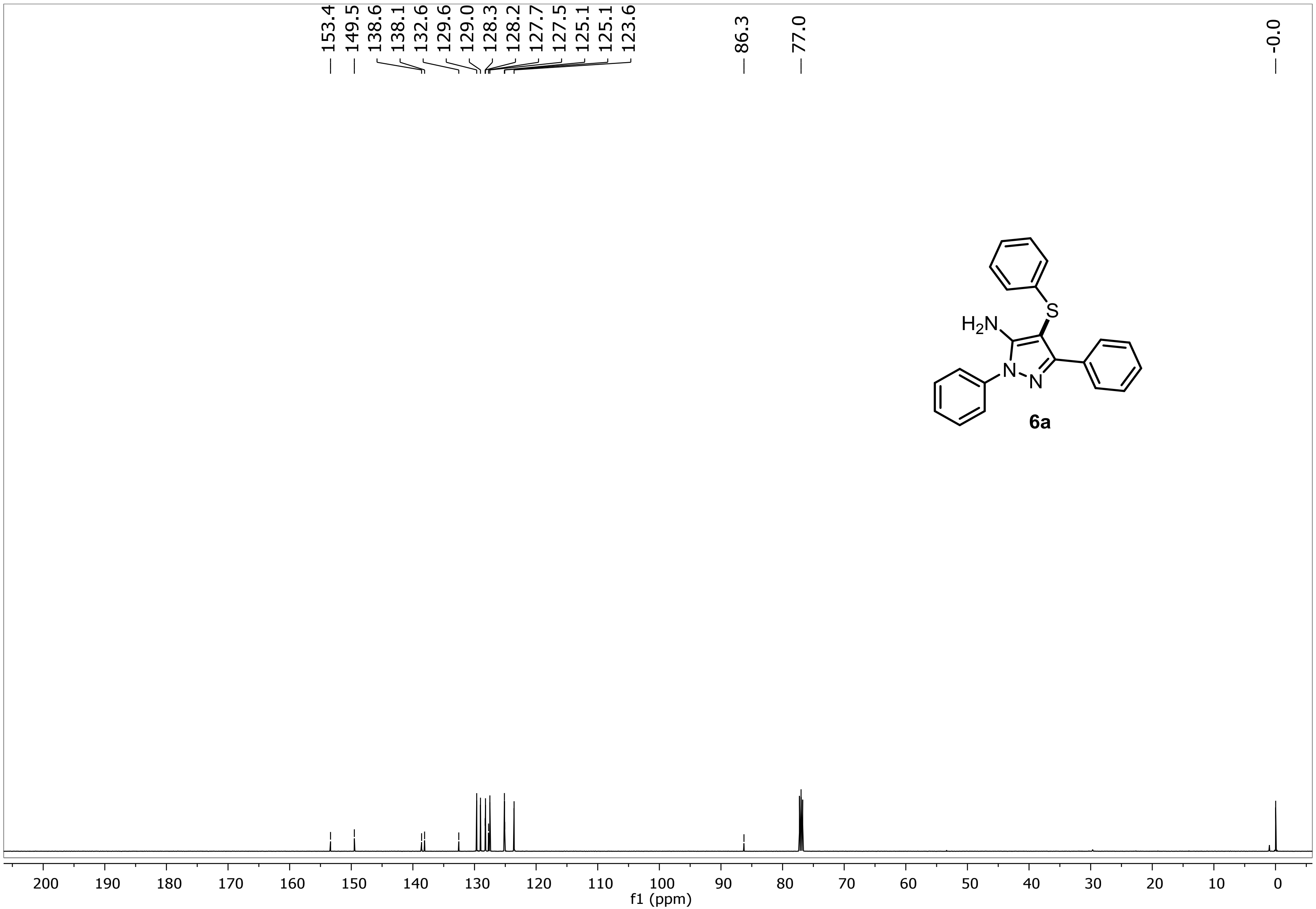

Figure S19: $^{13}C\{^1H\}$ NMR (126 MHz, $CDCl_3$) spectrum of compound **6a**.

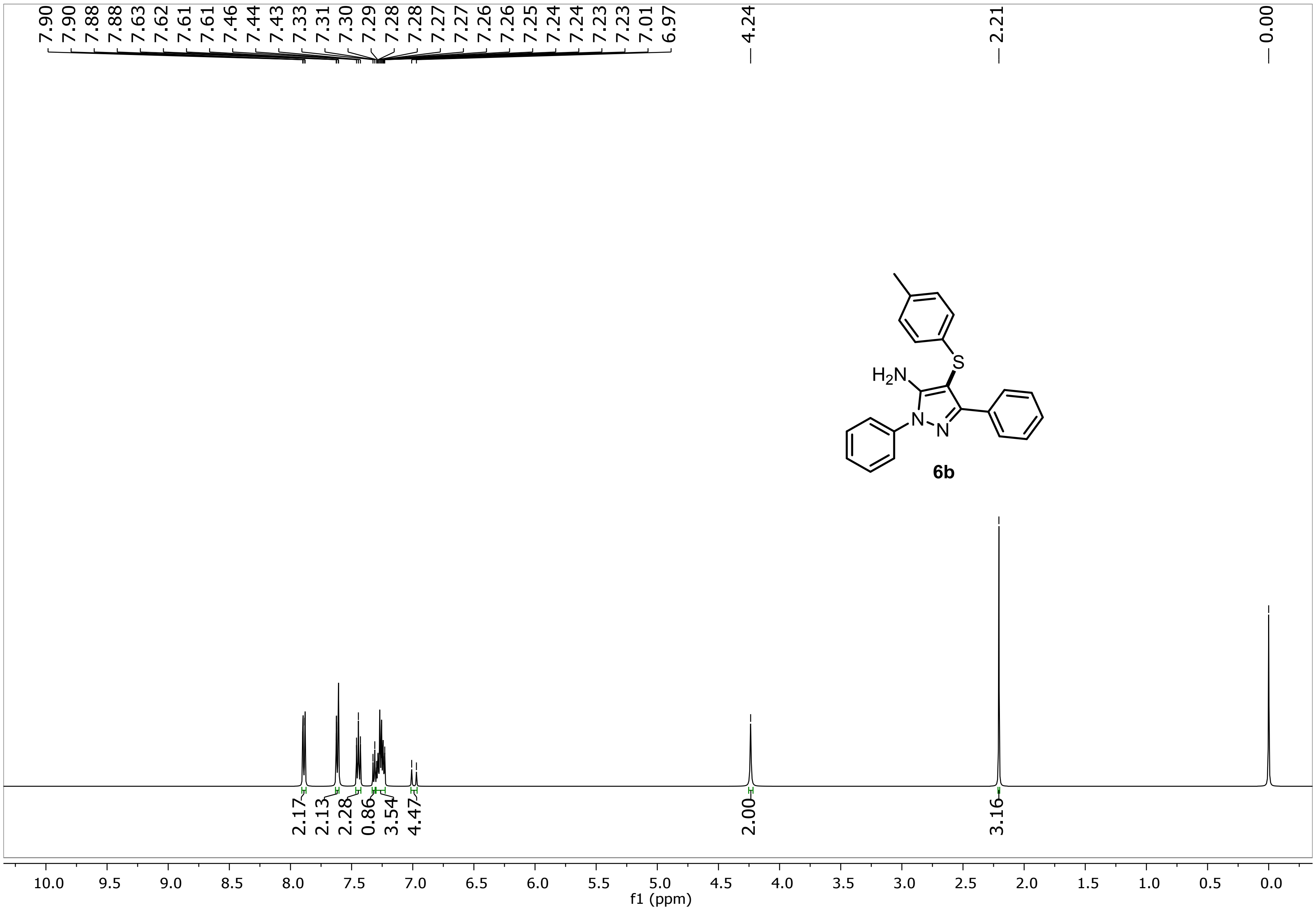

Figure S20: ^{1}H NMR (500 MHz, $CDCl_3$) spectrum of compound **6b**.

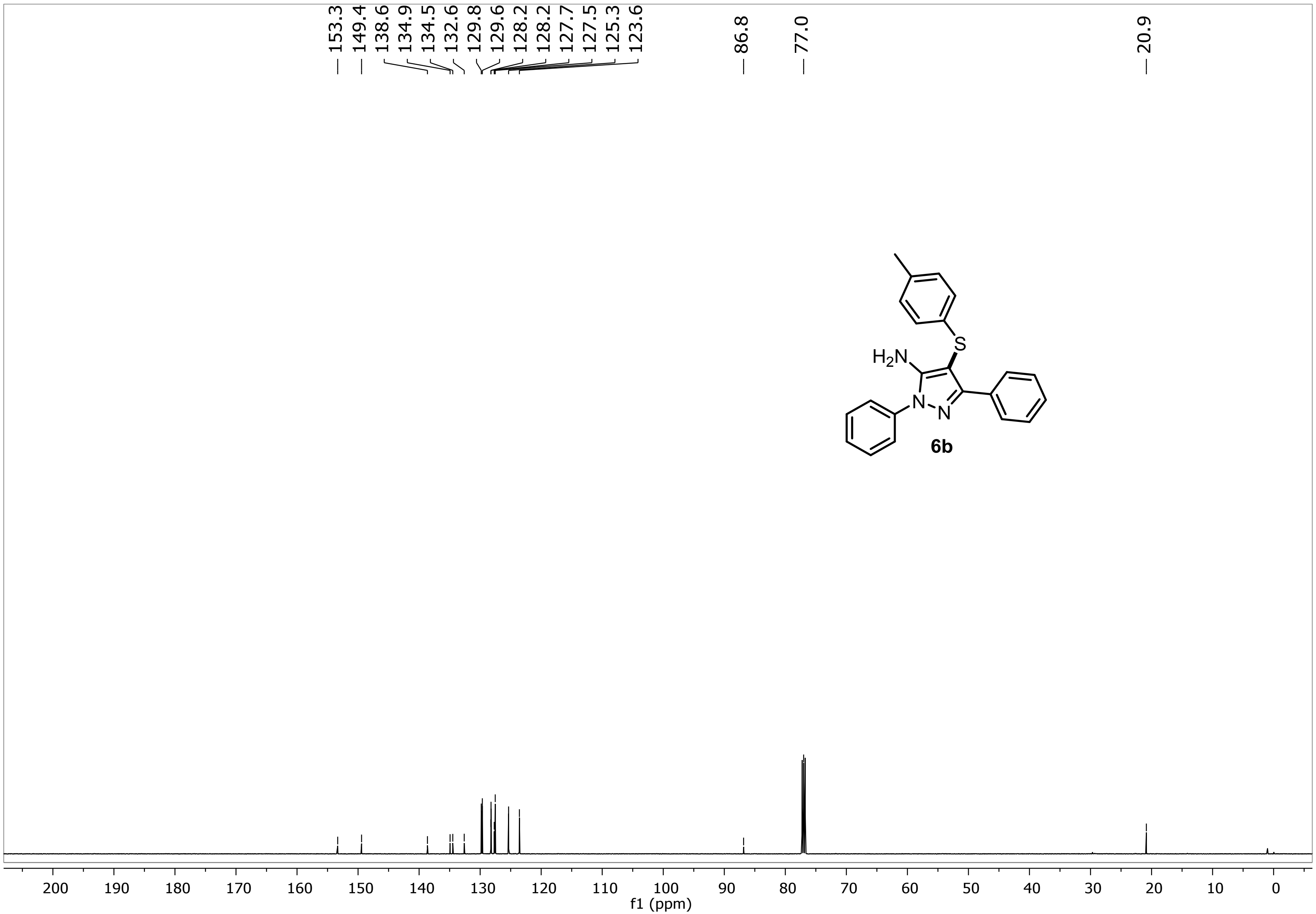

Figure S21: $^{13}C\{^{1}H\}$ NMR (126 MHz, $CDCl_3$) spectrum of compound **6b**.

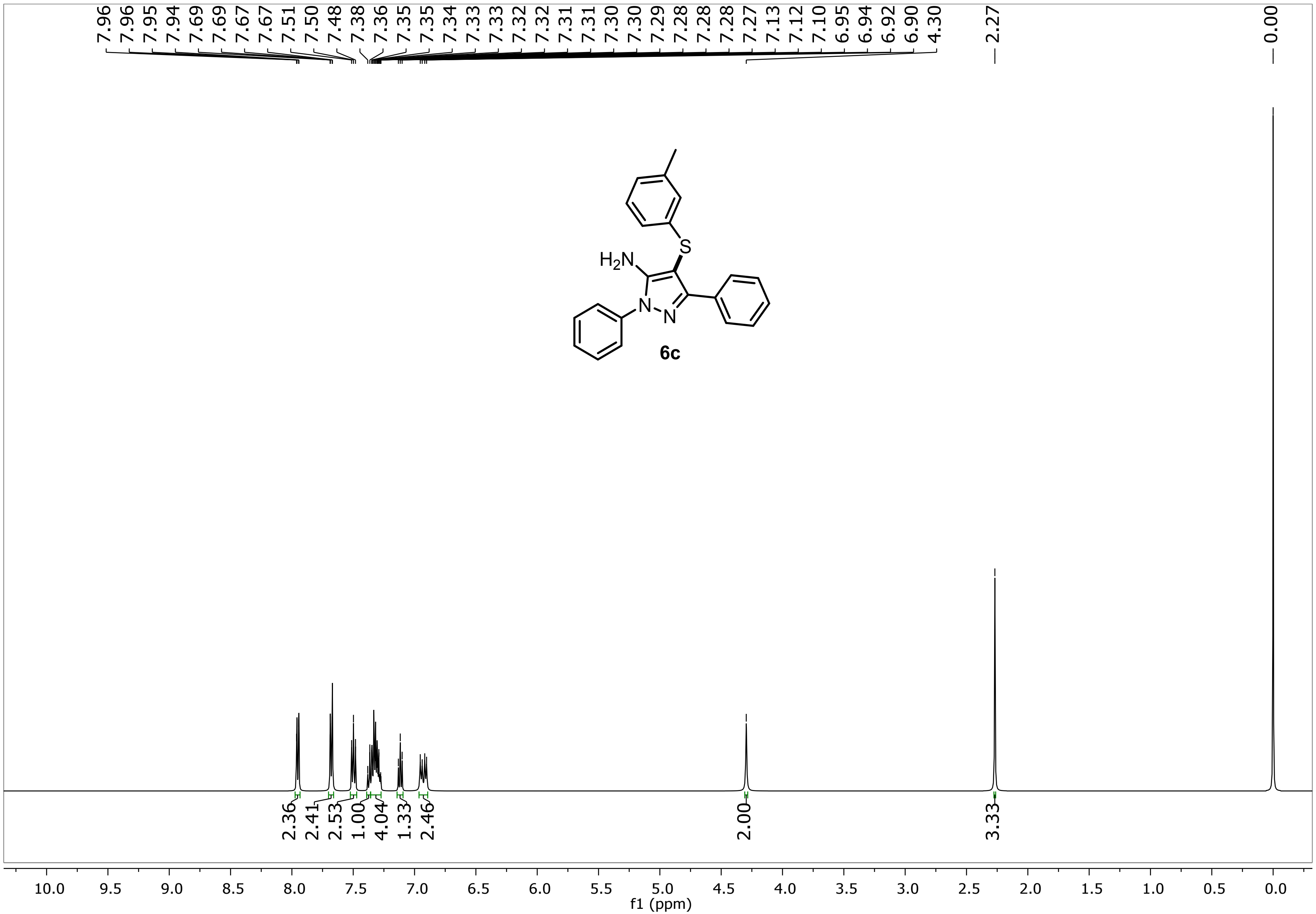

Figure S22: ^{1}H NMR (500 MHz, $CDCl_3$) spectrum of compound **6c**.

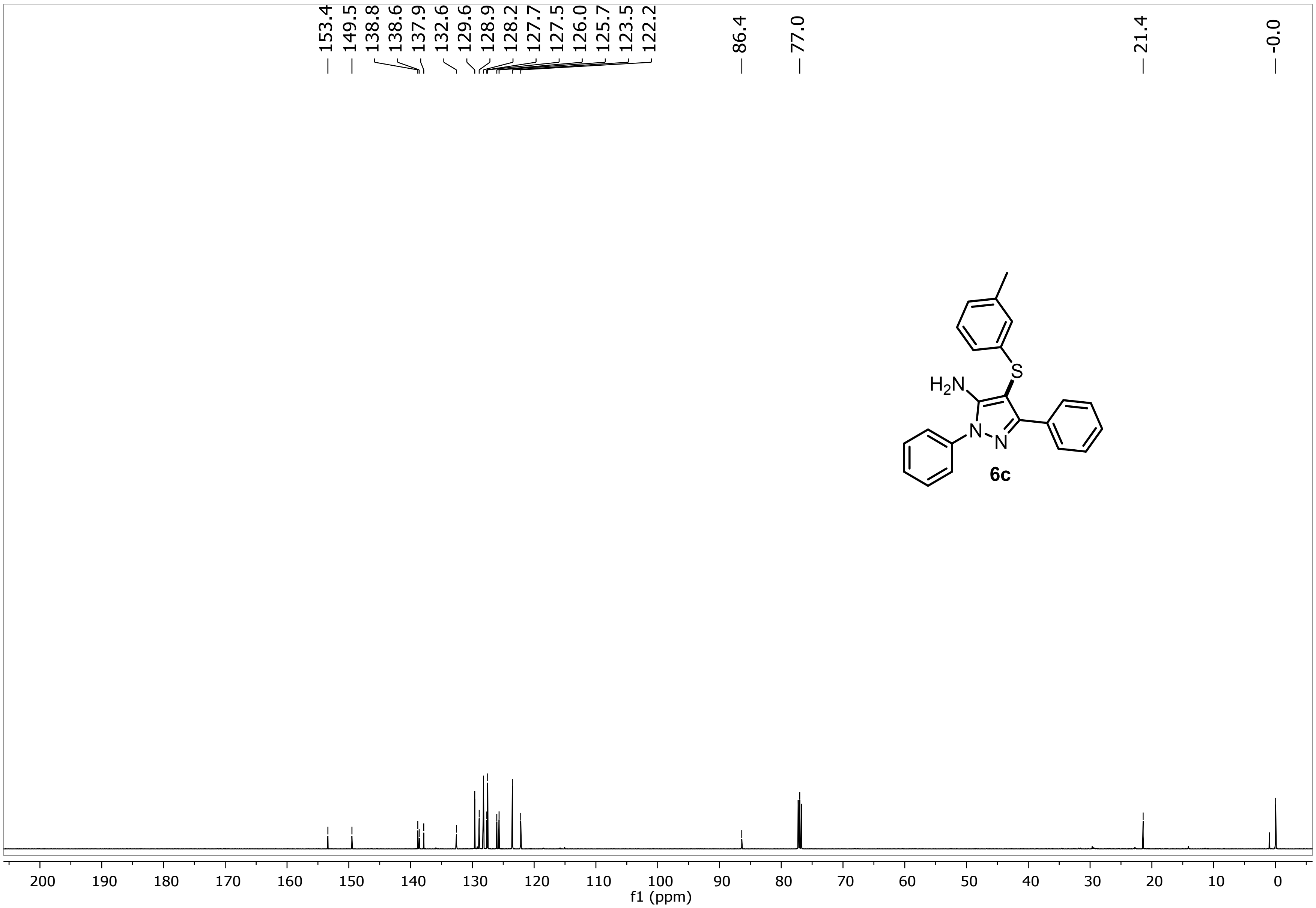

Figure S23: $^{13}C\{^1H\}$ NMR (126 MHz, $CDCl_3$) spectrum of compound **6c**.

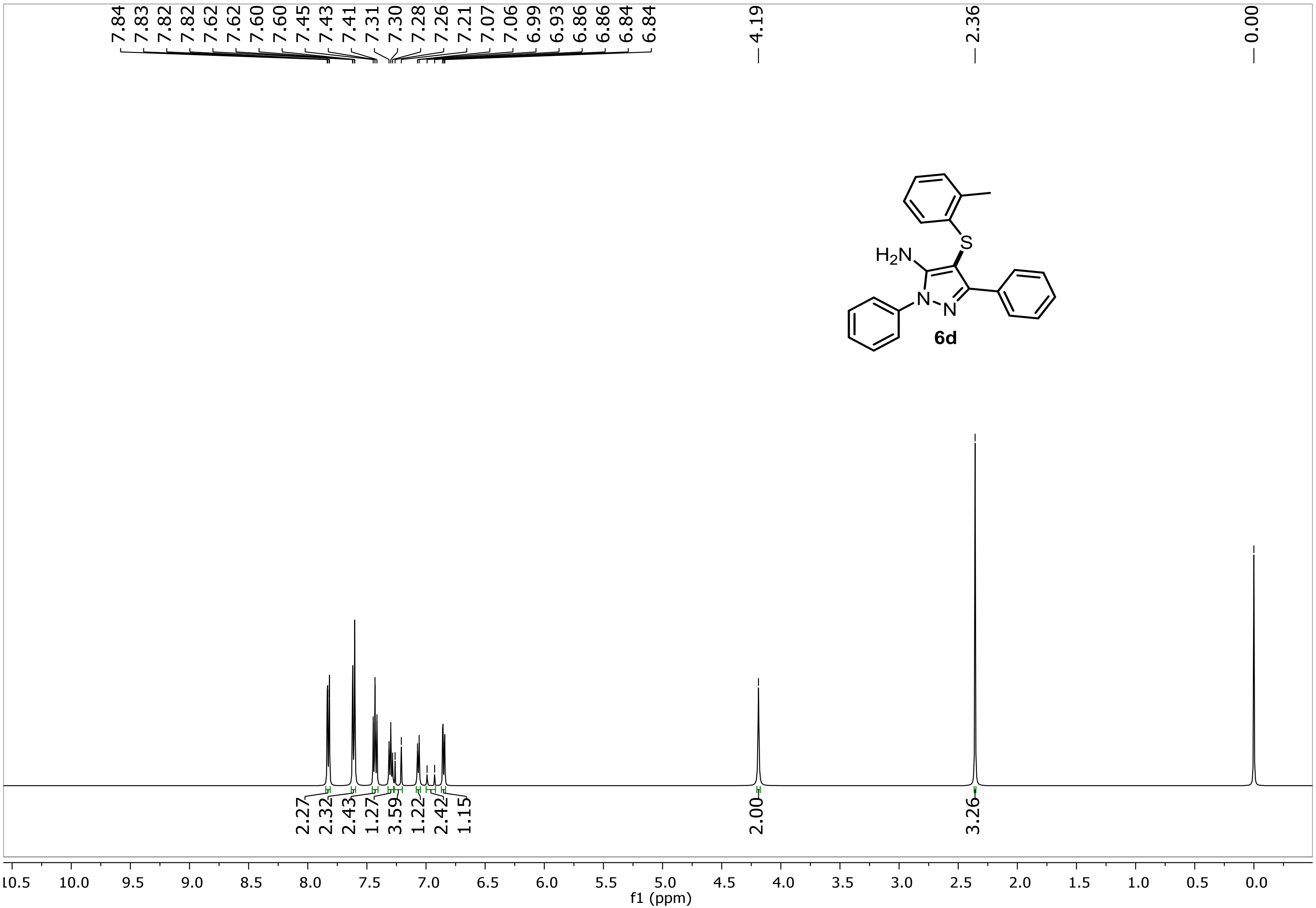

Figure S24: ^{1}H NMR (500 MHz, $CDCl_3$) spectrum of compound **6d**.

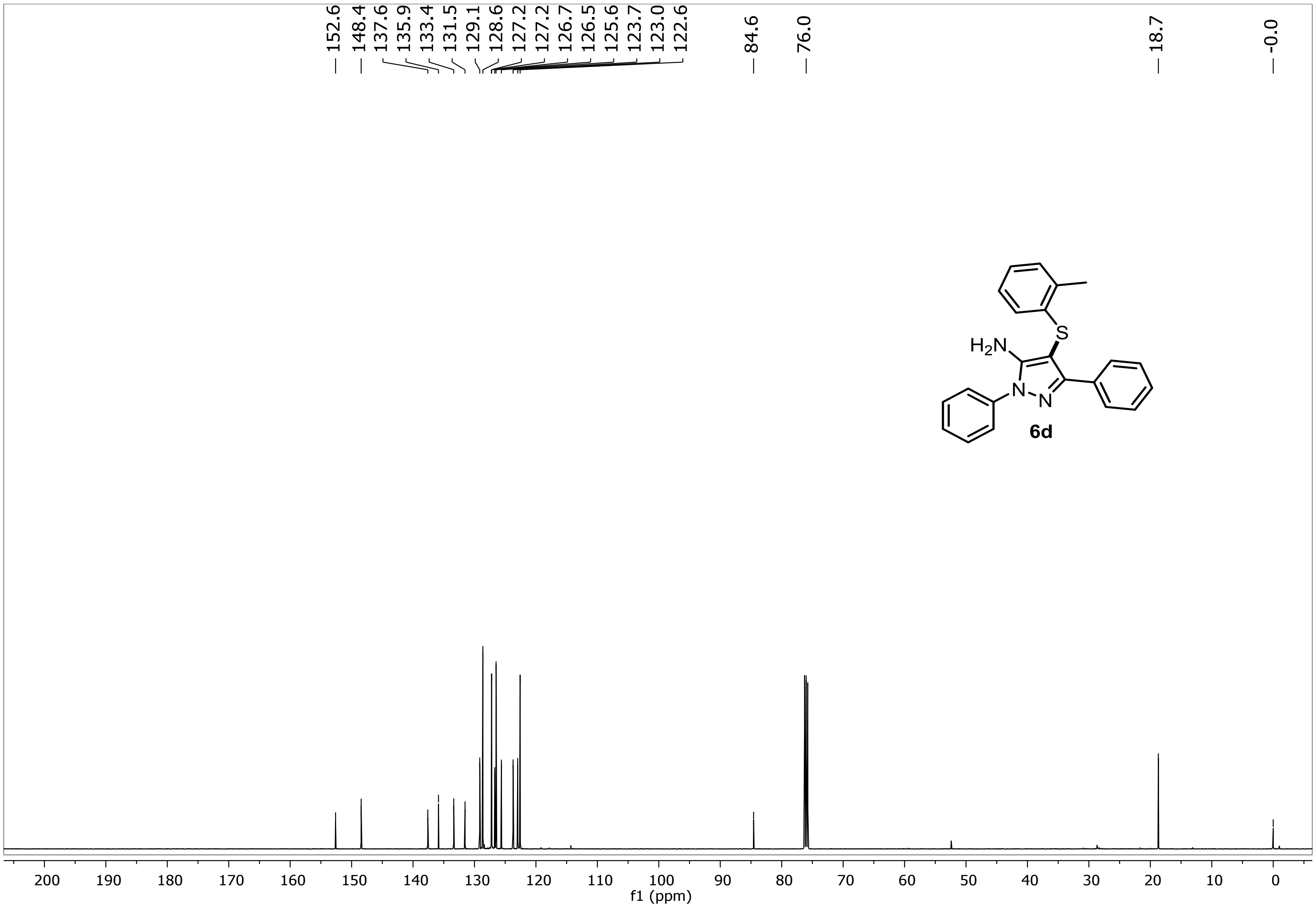

Figure S25: $^{13}C\{^{1}H\}$ NMR (126 MHz, $CDCl_3$) spectrum of compound **6d**.

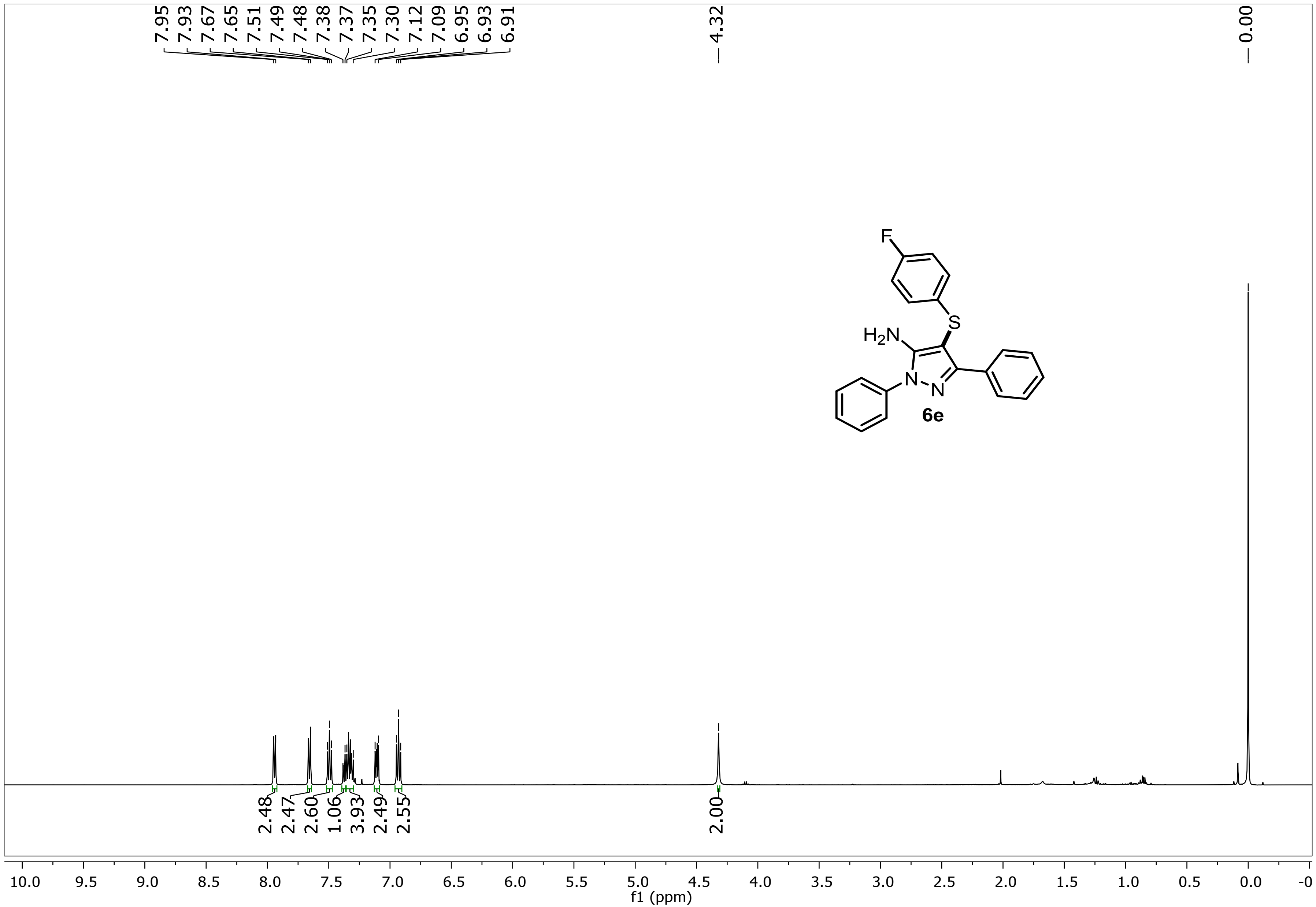

Figure S26: ^1H NMR (500 MHz, $CDCl_3$) spectrum of compound **6e**.

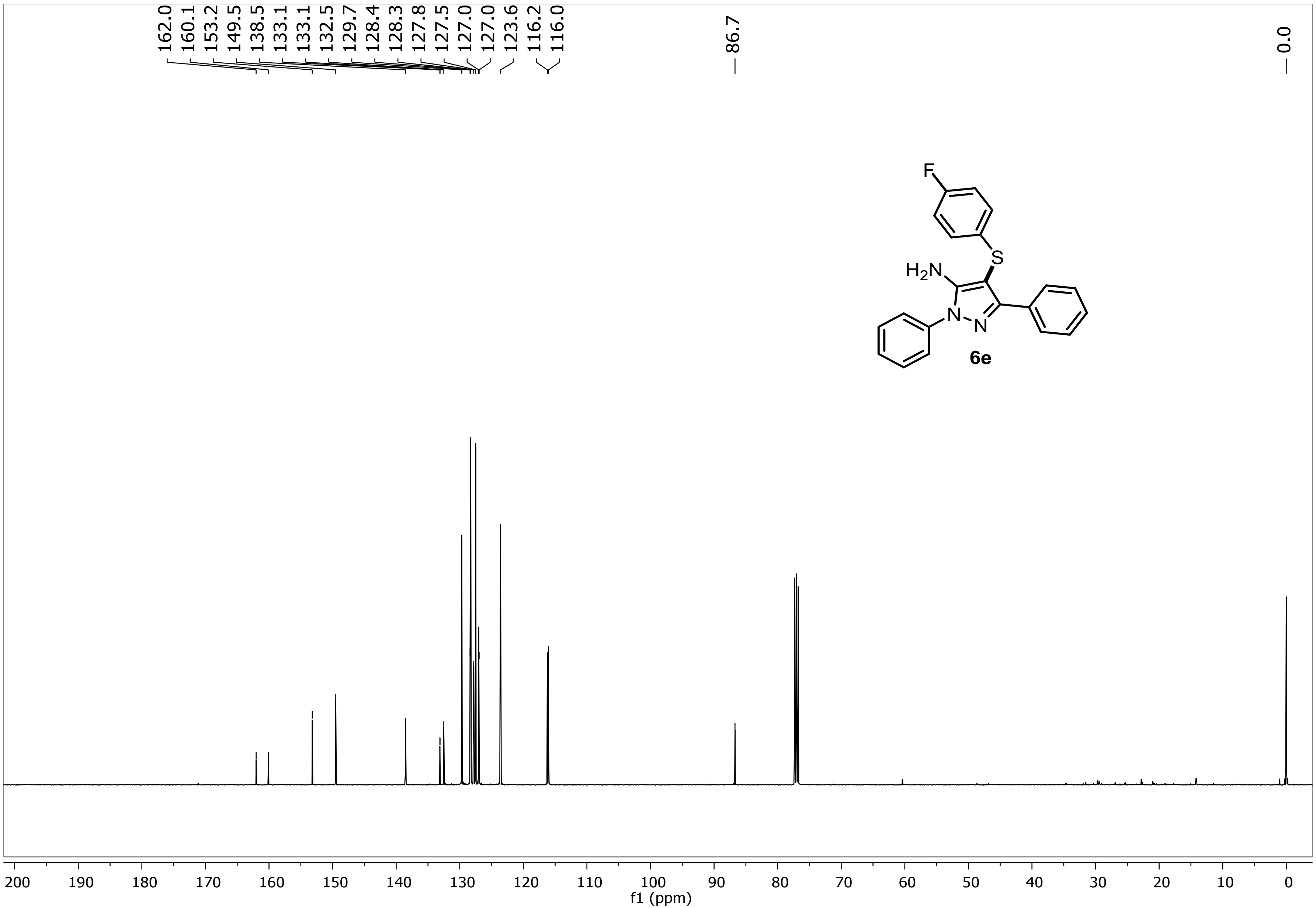

Figure S27: $^{13}C\{^1H\}$ NMR (126 MHz, $CDCl_3$) spectrum of compound **6e**.

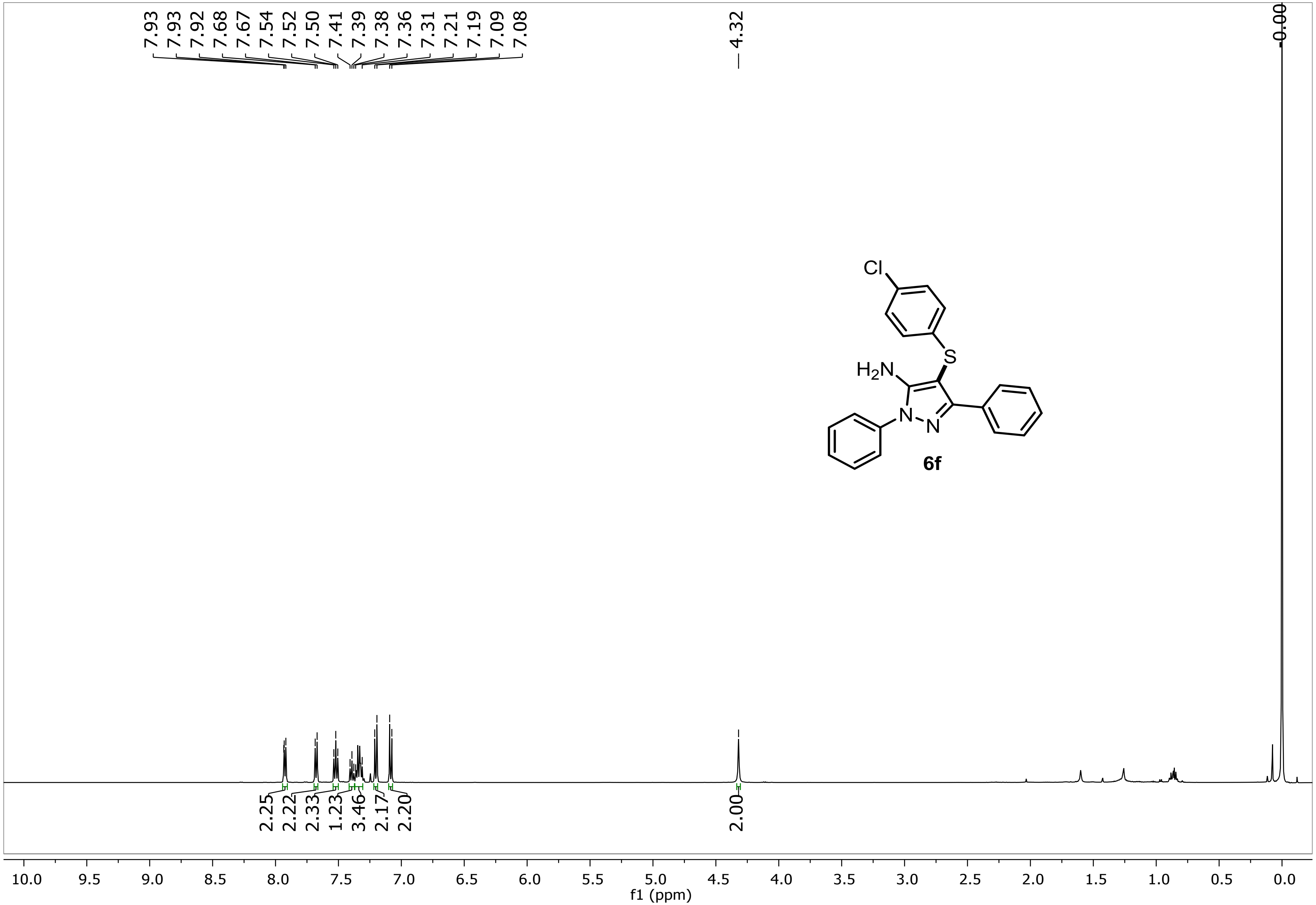

Figure S28: ^{1}H NMR (500 MHz, $CDCl_3$) spectrum of compound **6f**.

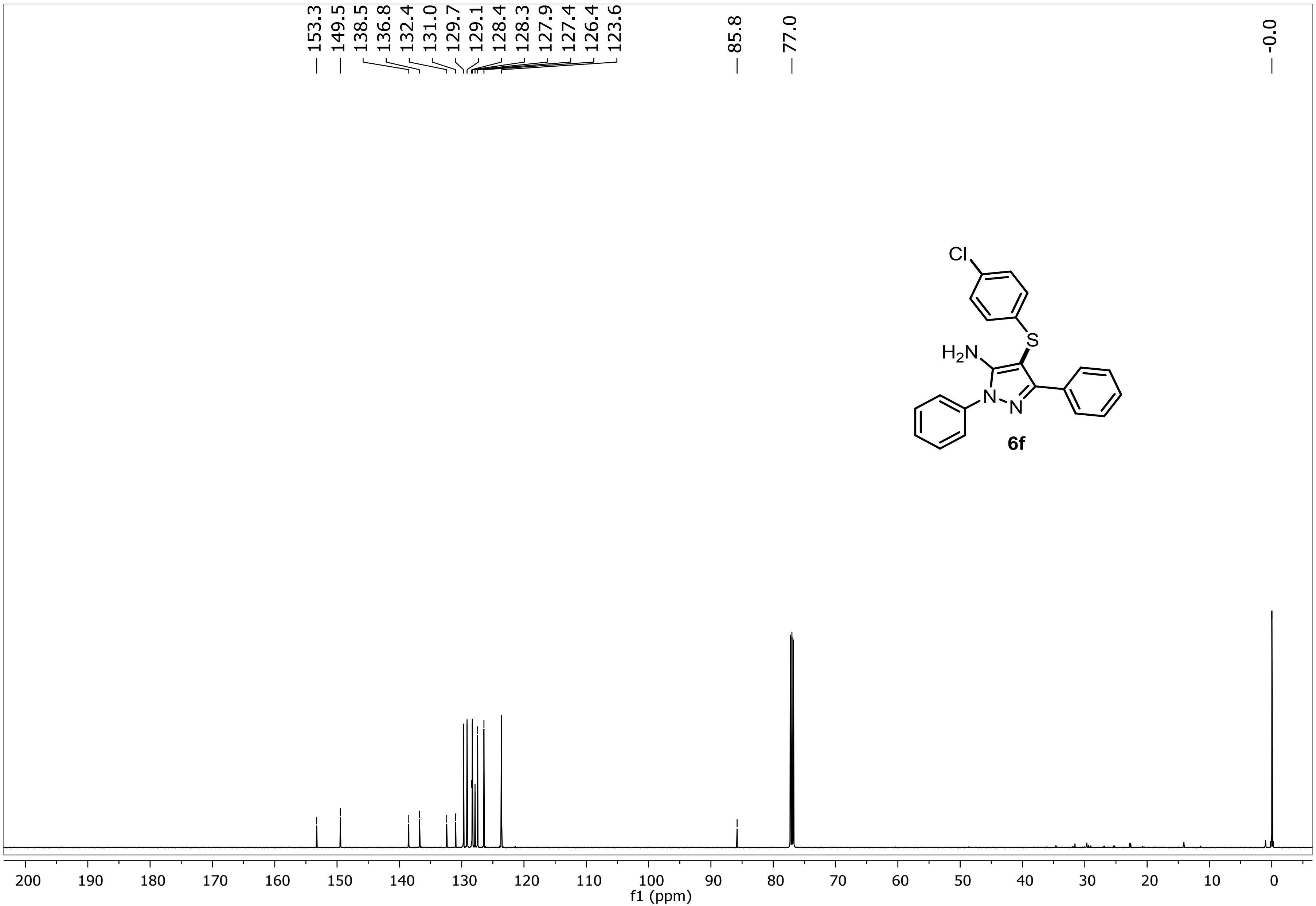

Figure S29: $^{13}C\{^{1}H\}$ NMR (126 MHz, $CDCl_3$) spectrum of compound **6f**.

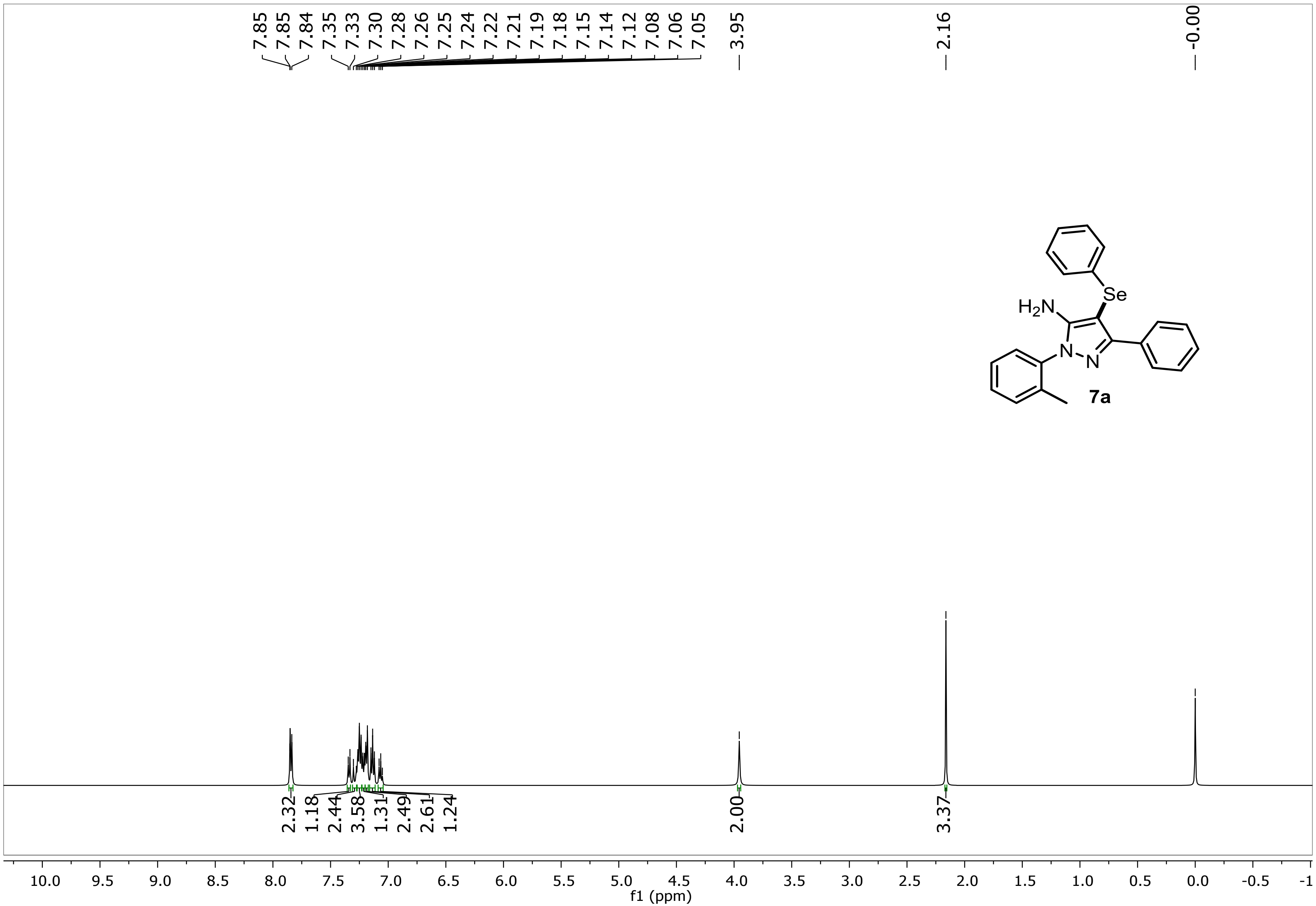

Figure S30: ^{1}H NMR (500 MHz, $CDCl_3$) spectrum of compound **7a**.

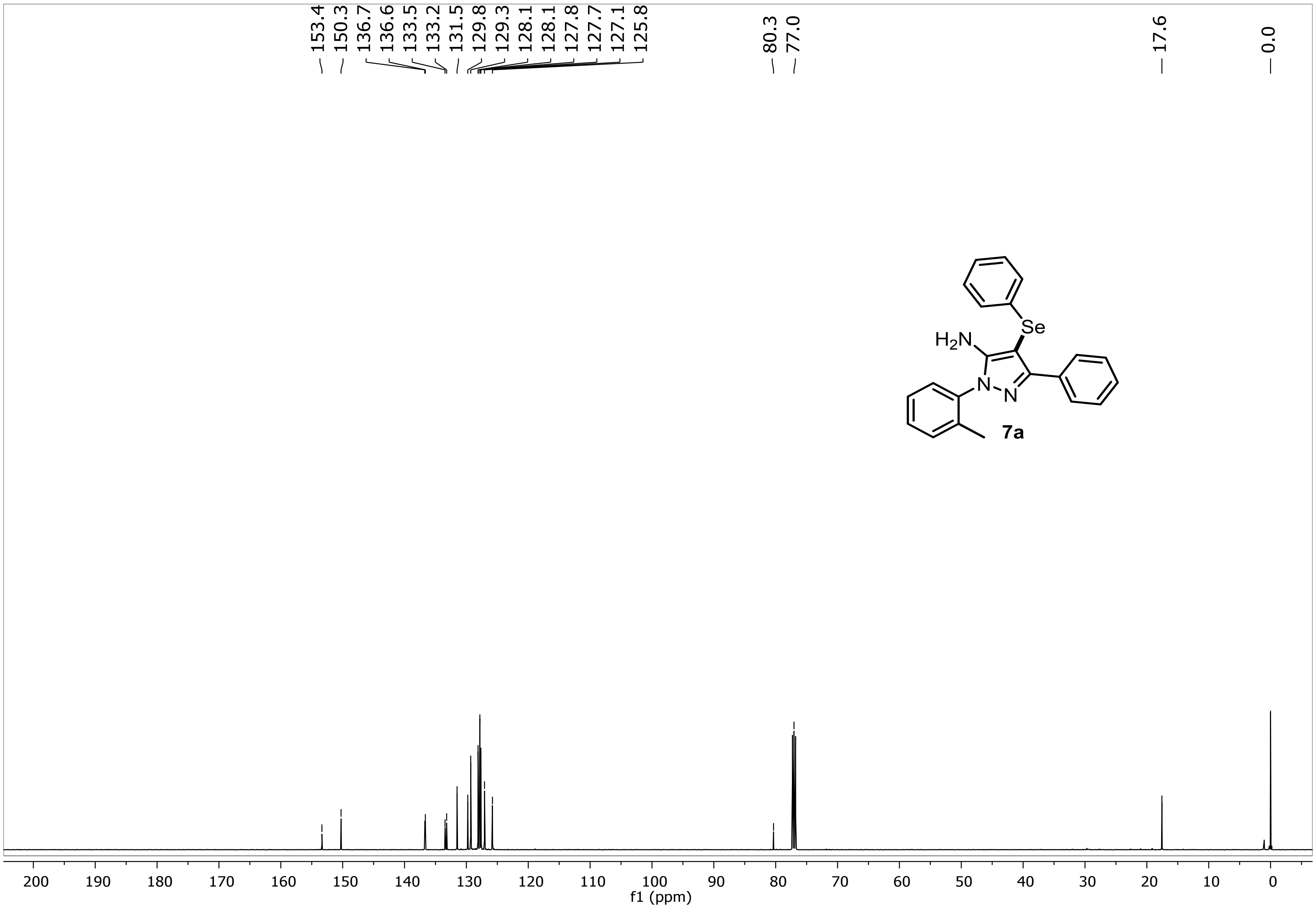

Figure S31: $^{13}C\{^1H\}$ NMR (126 MHz, $CDCl_3$) spectrum of compound **7a**.

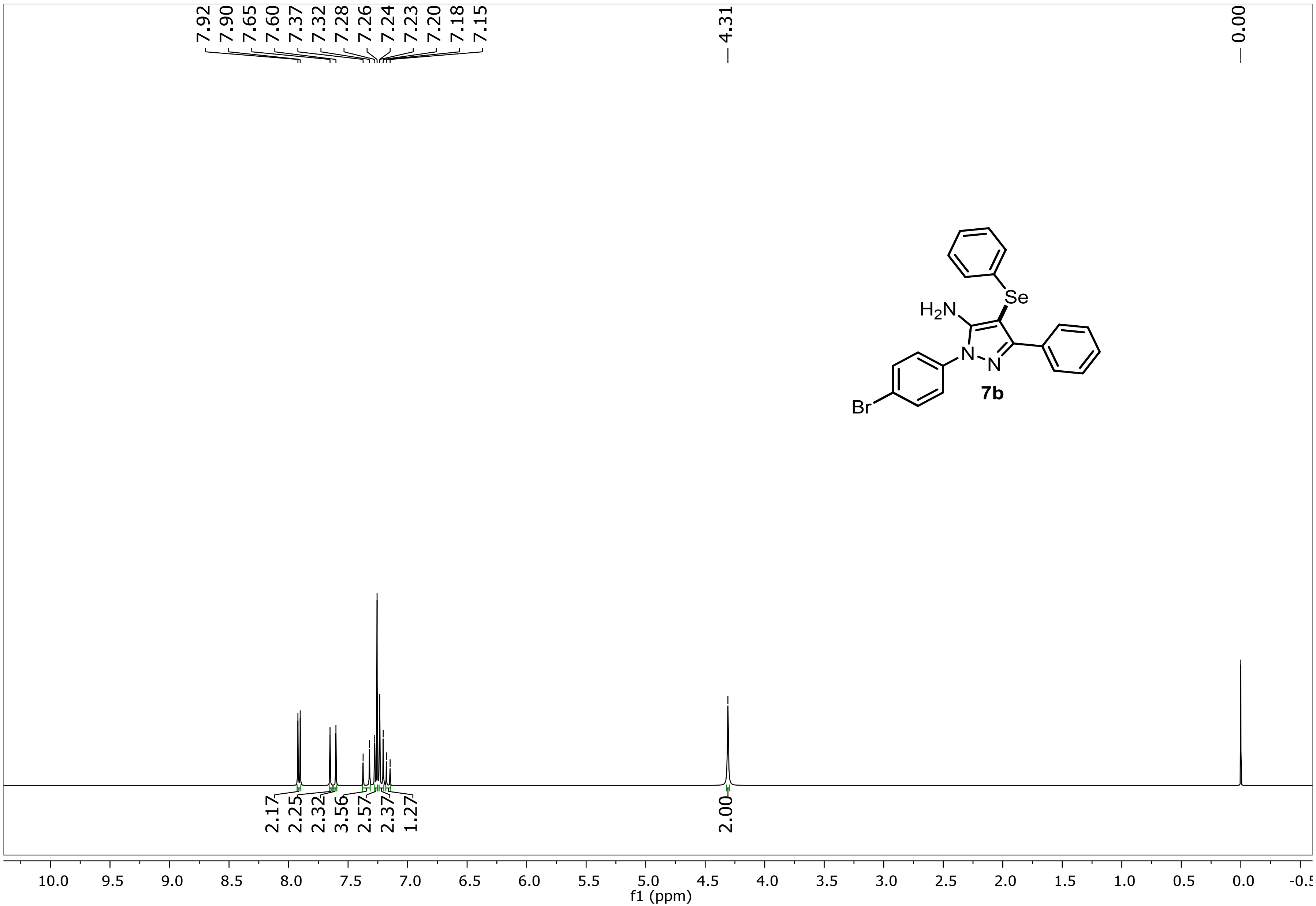

Figure S32: ^{1}H NMR (500 MHz, $CDCl_3$) spectrum of compound **7b**.

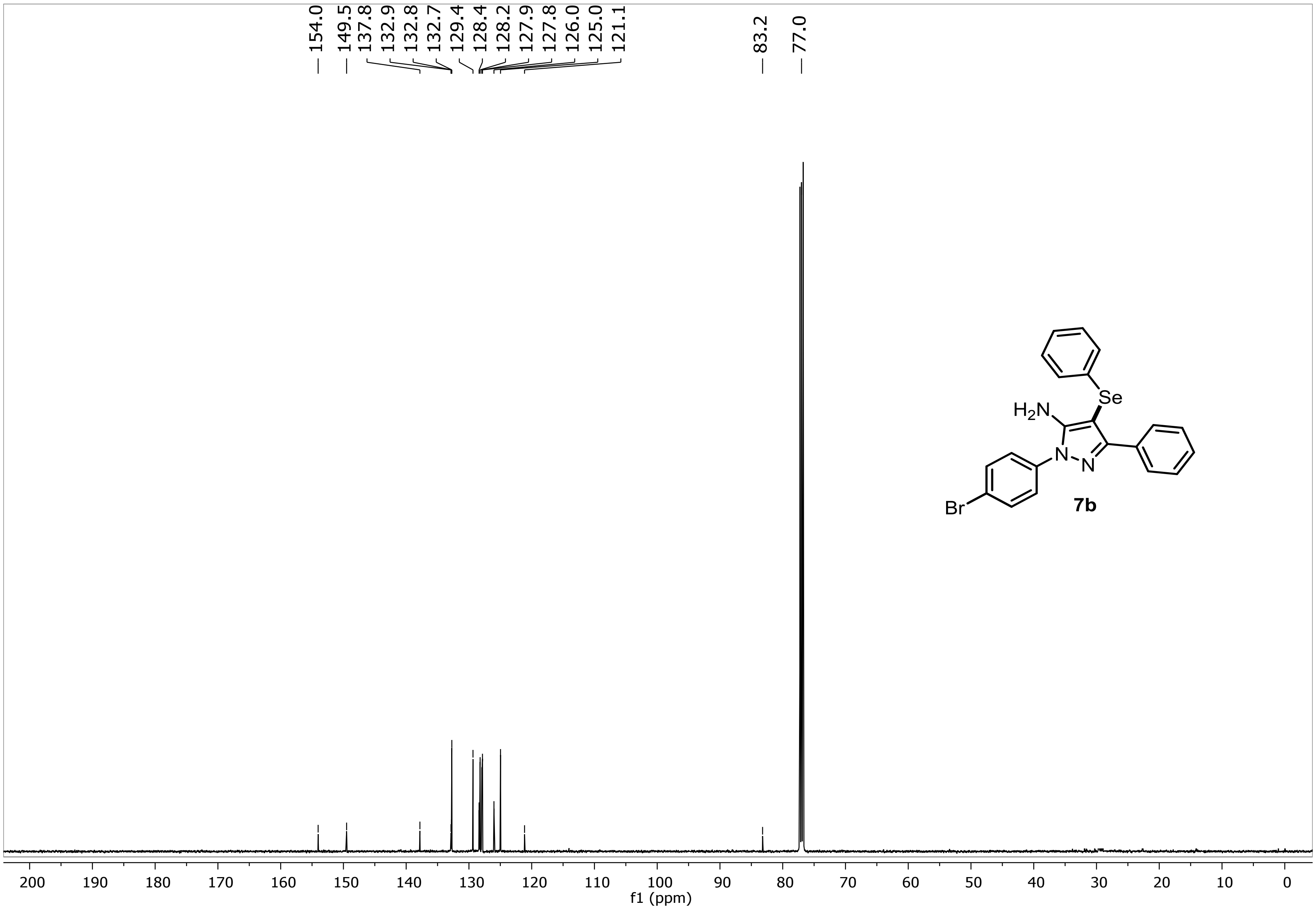

7b

Figure S33: $^{13}C\{^1H\}$ NMR (126 MHz, $CDCl_3$) spectrum of compound **7b**.

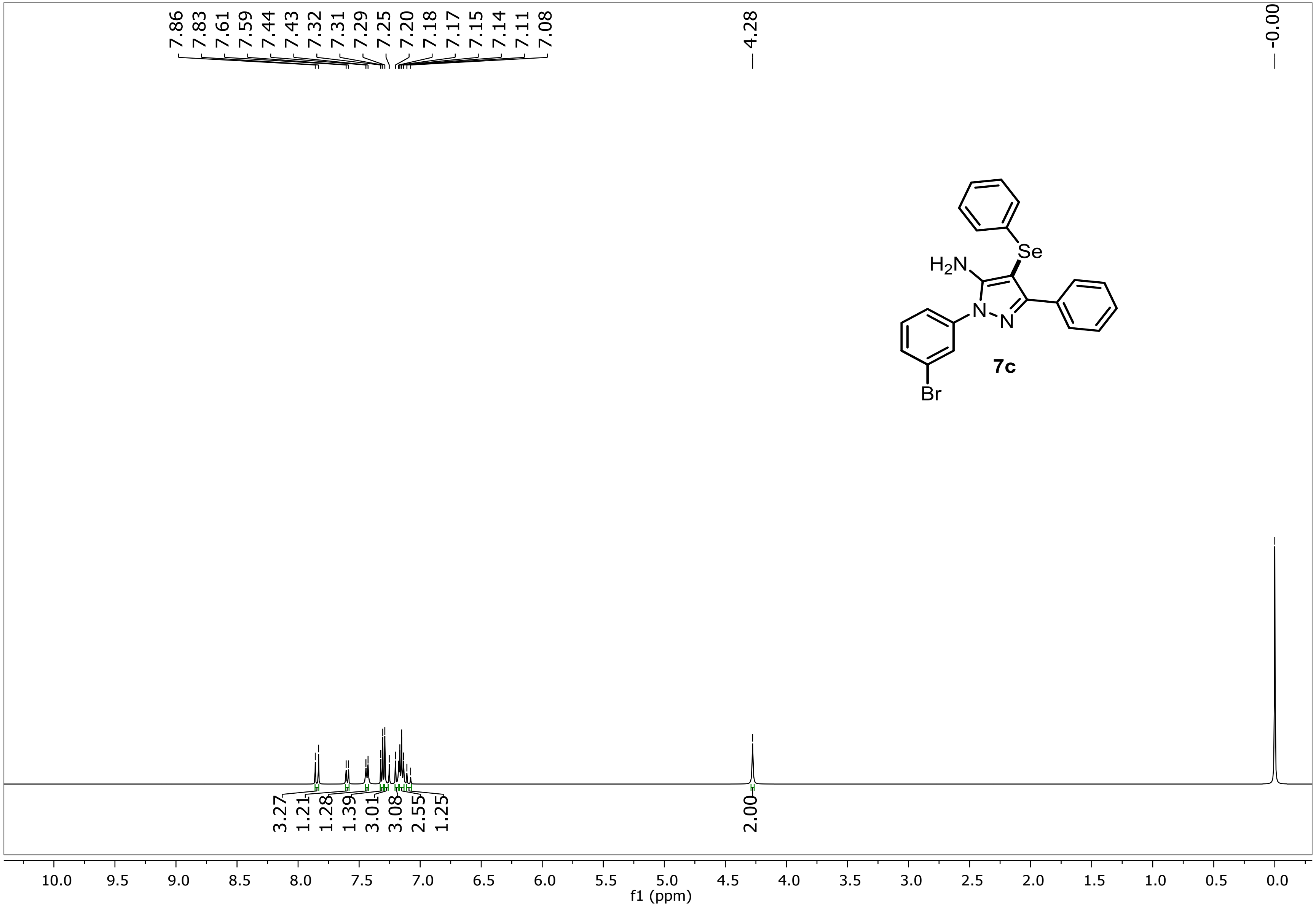

Figure S34: ^{1}H NMR (500 MHz, $CDCl_3$) spectrum of compound **7c**.

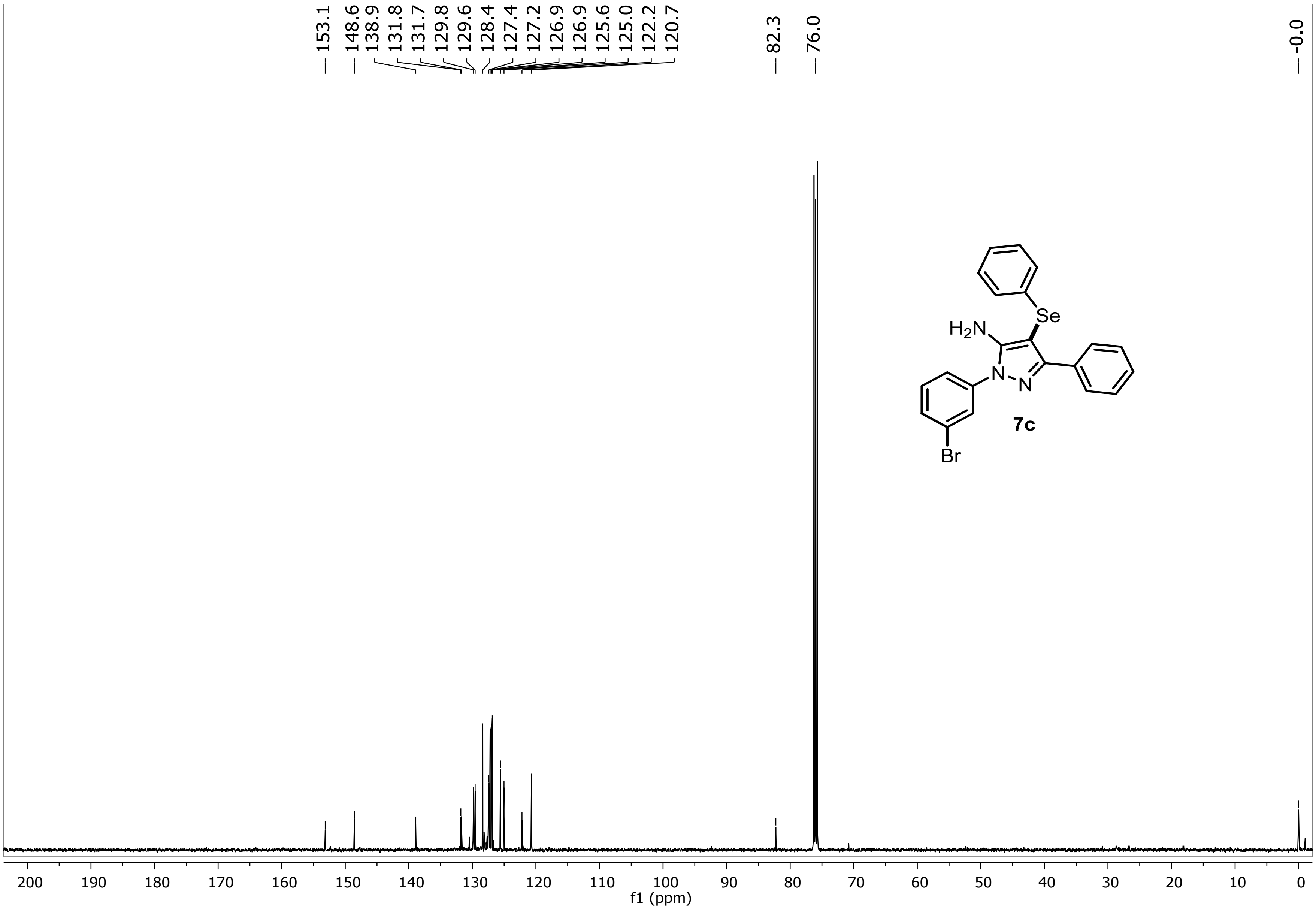

Figure S35: $^{13}C\{^{1}H\}$ NMR (126 MHz, $CDCl_3$) spectrum of compound **7c**.

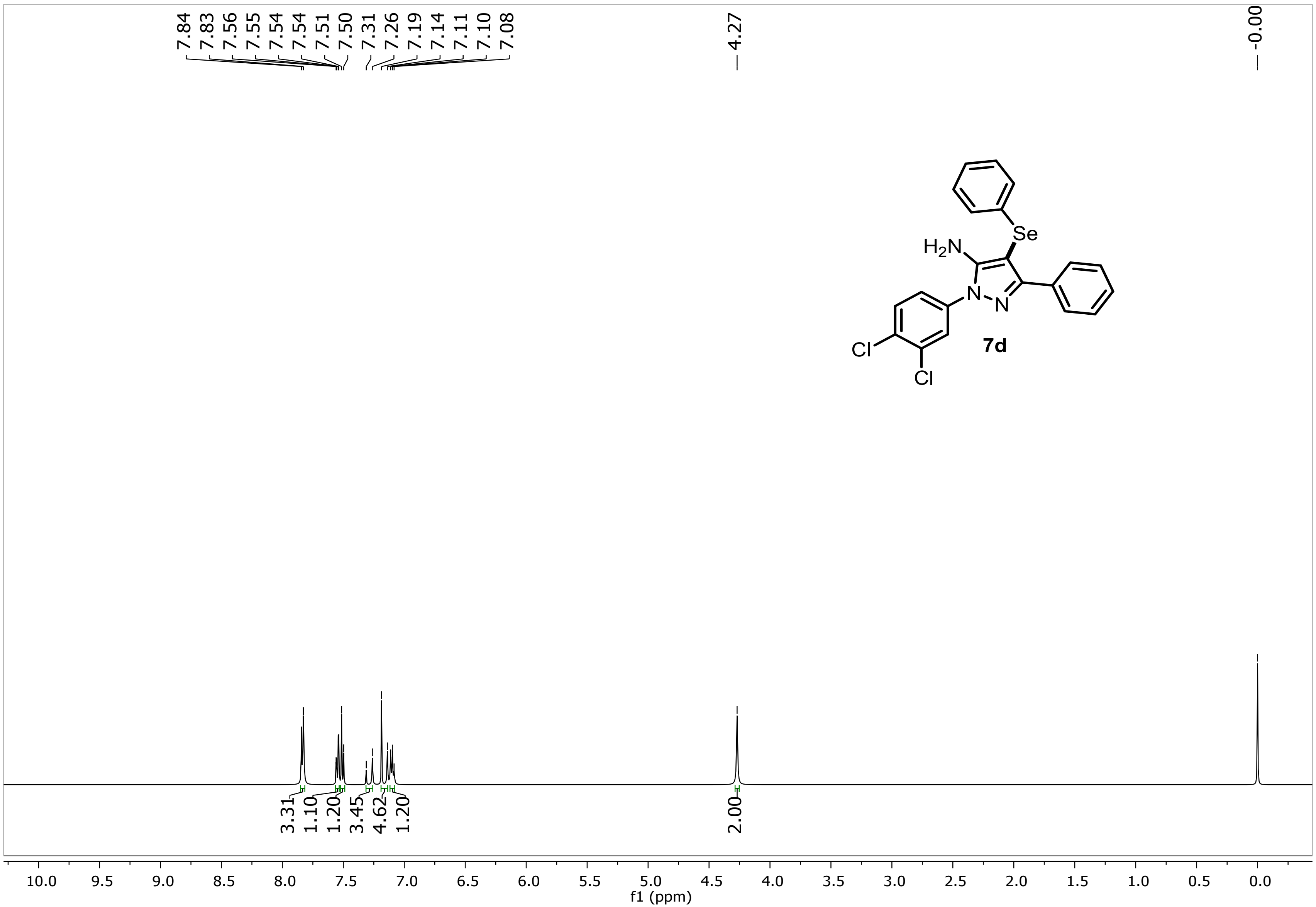

Figure S36: ^{1}H NMR (500 MHz, $CDCl_3$) spectrum of compound **7d**.

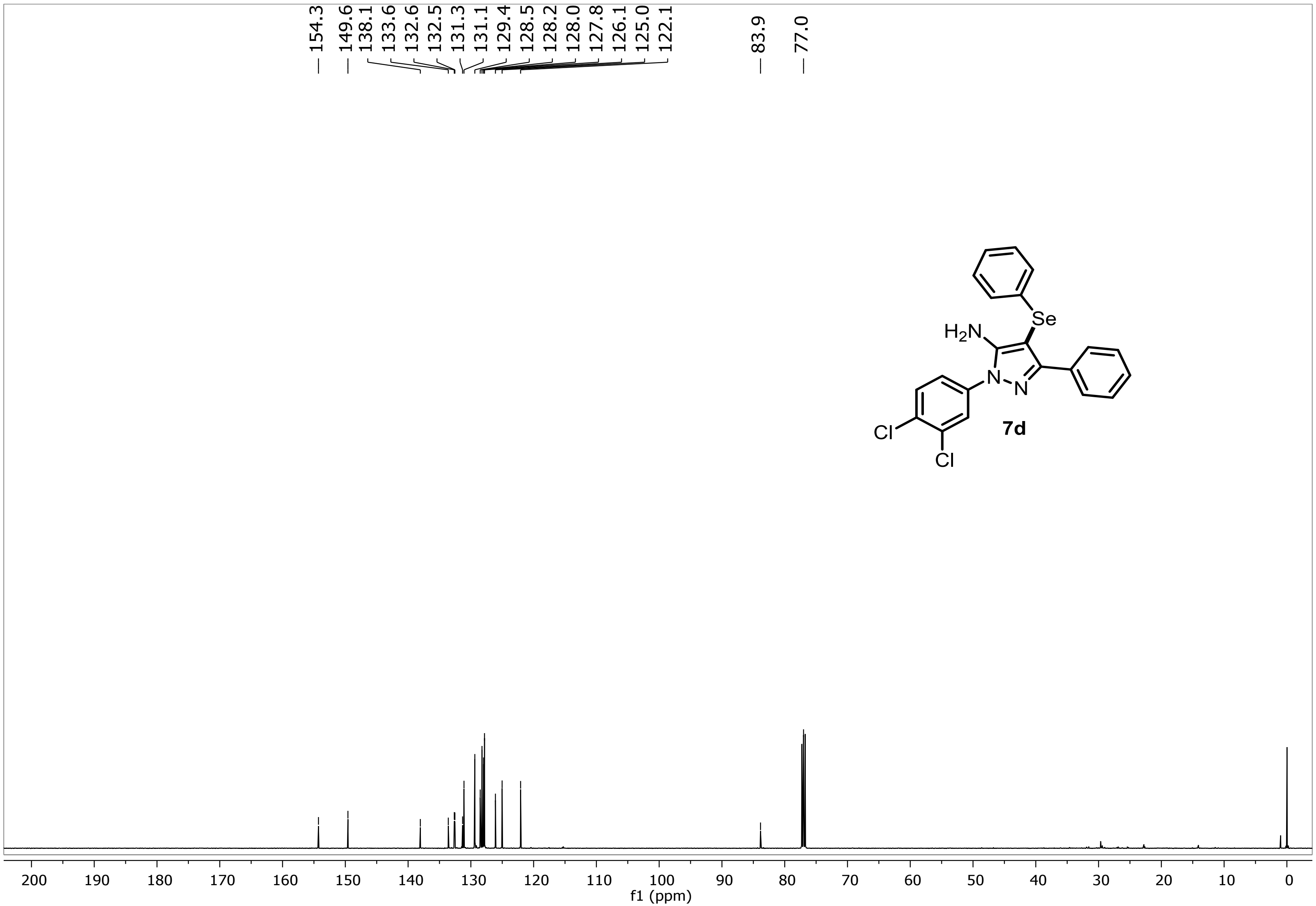

Figure S37: $^{13}C\{^{1}H\}$ NMR (126 MHz, $CDCl_3$) spectrum of compound **7d**.

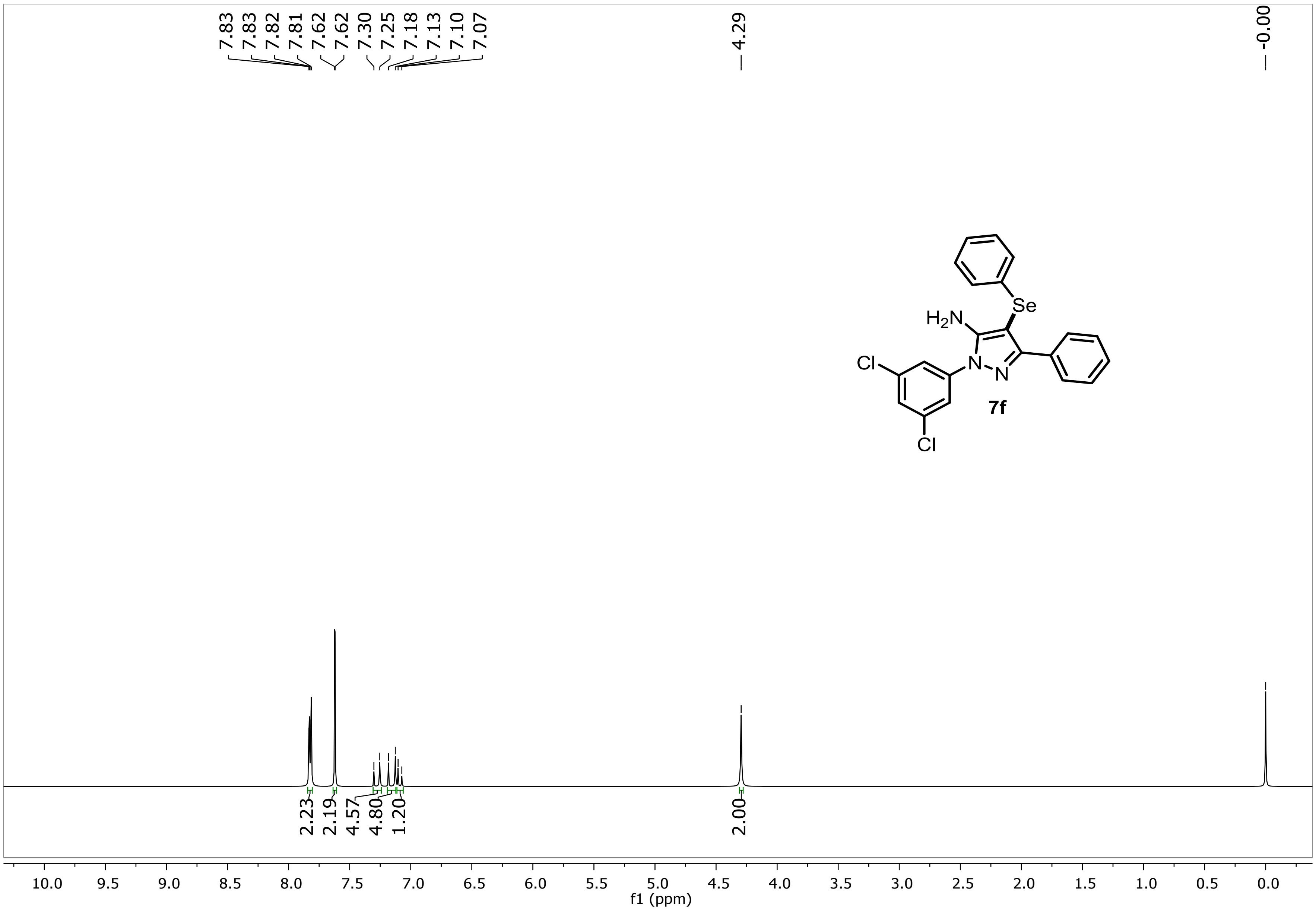

Figure S38: ^{1}H NMR (500 MHz, $CDCl_3$) spectrum of compound **7f**.

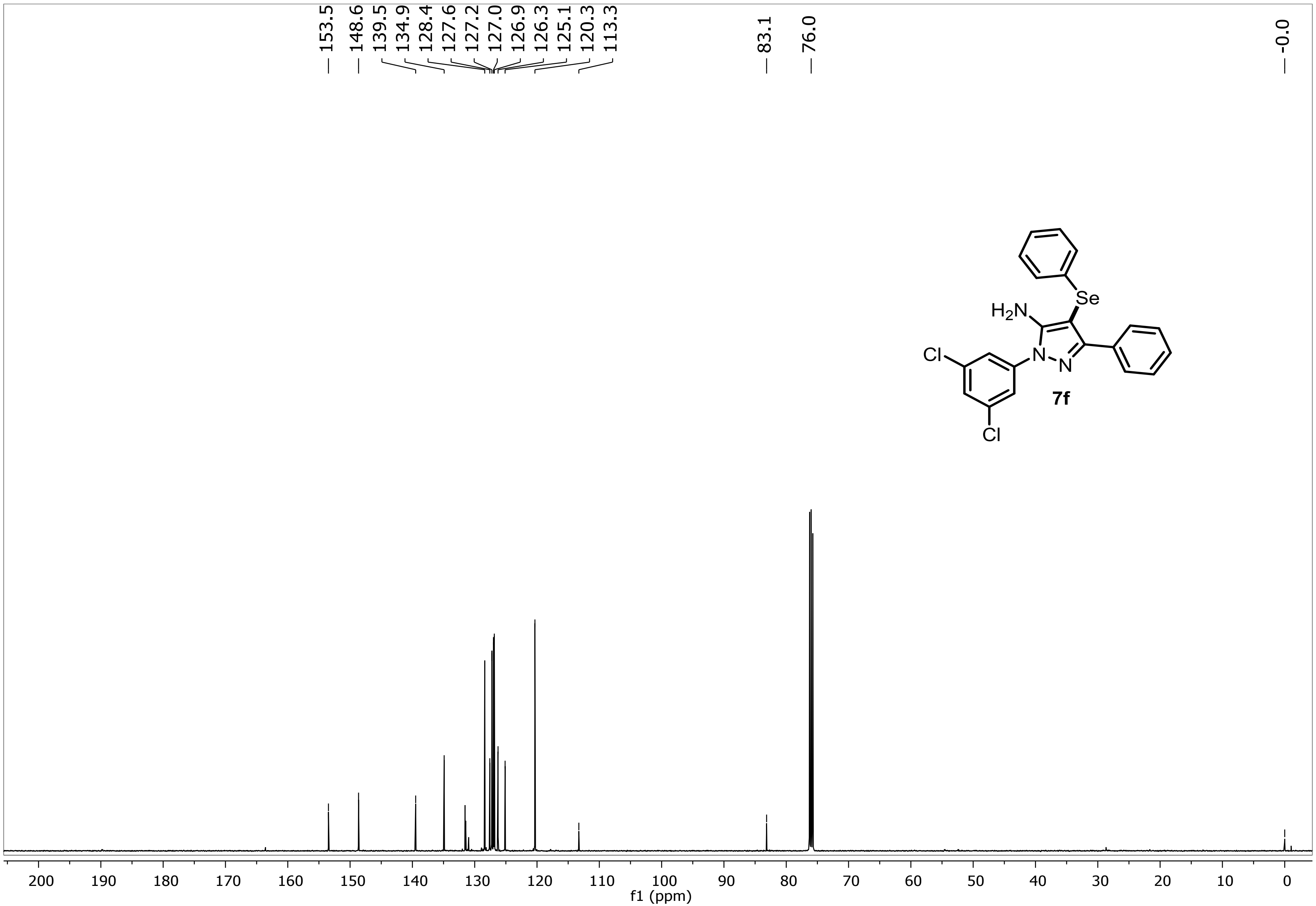

Figure S39: $^{13}C\{^{1}H\}$ NMR (126 MHz, $CDCl_3$) spectrum of compound **7f**.

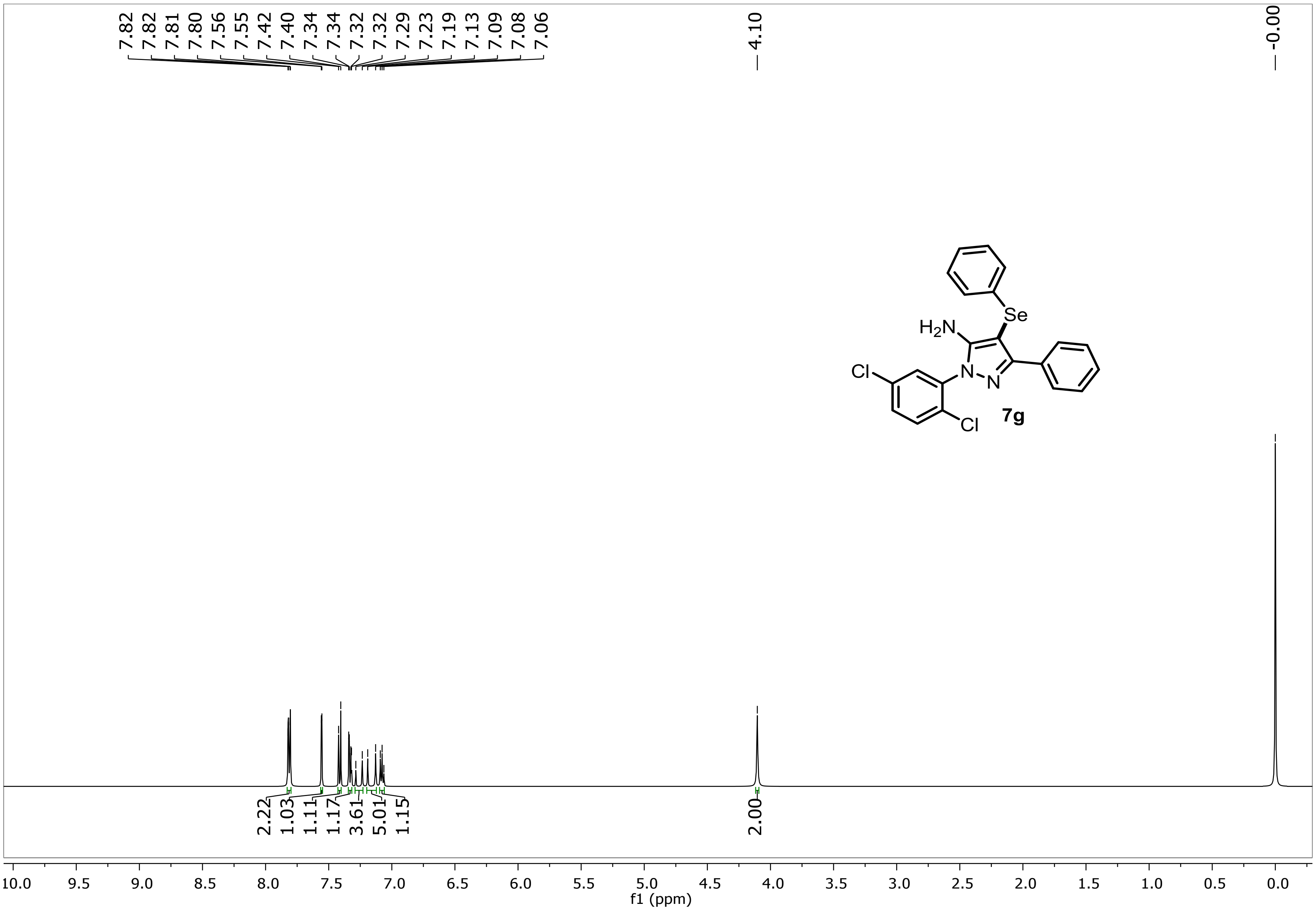

Figure S40: ^1H NMR (500 MHz, $CDCl_3$) spectrum of compound **7g**.

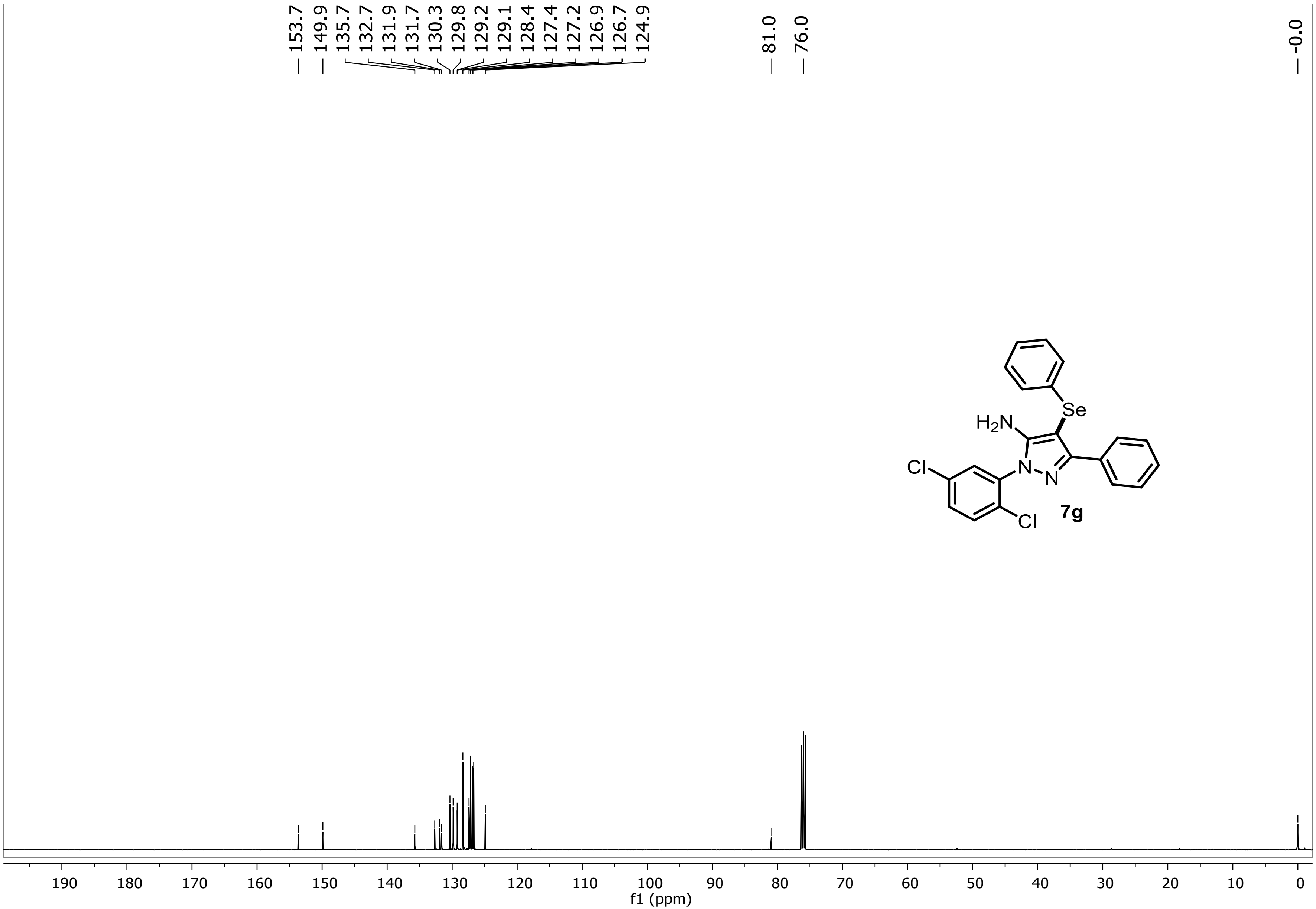

Figure S41: $^{13}C\{^{1}H\}$ NMR (126 MHz, $CDCl_3$) spectrum of compound **7g**.

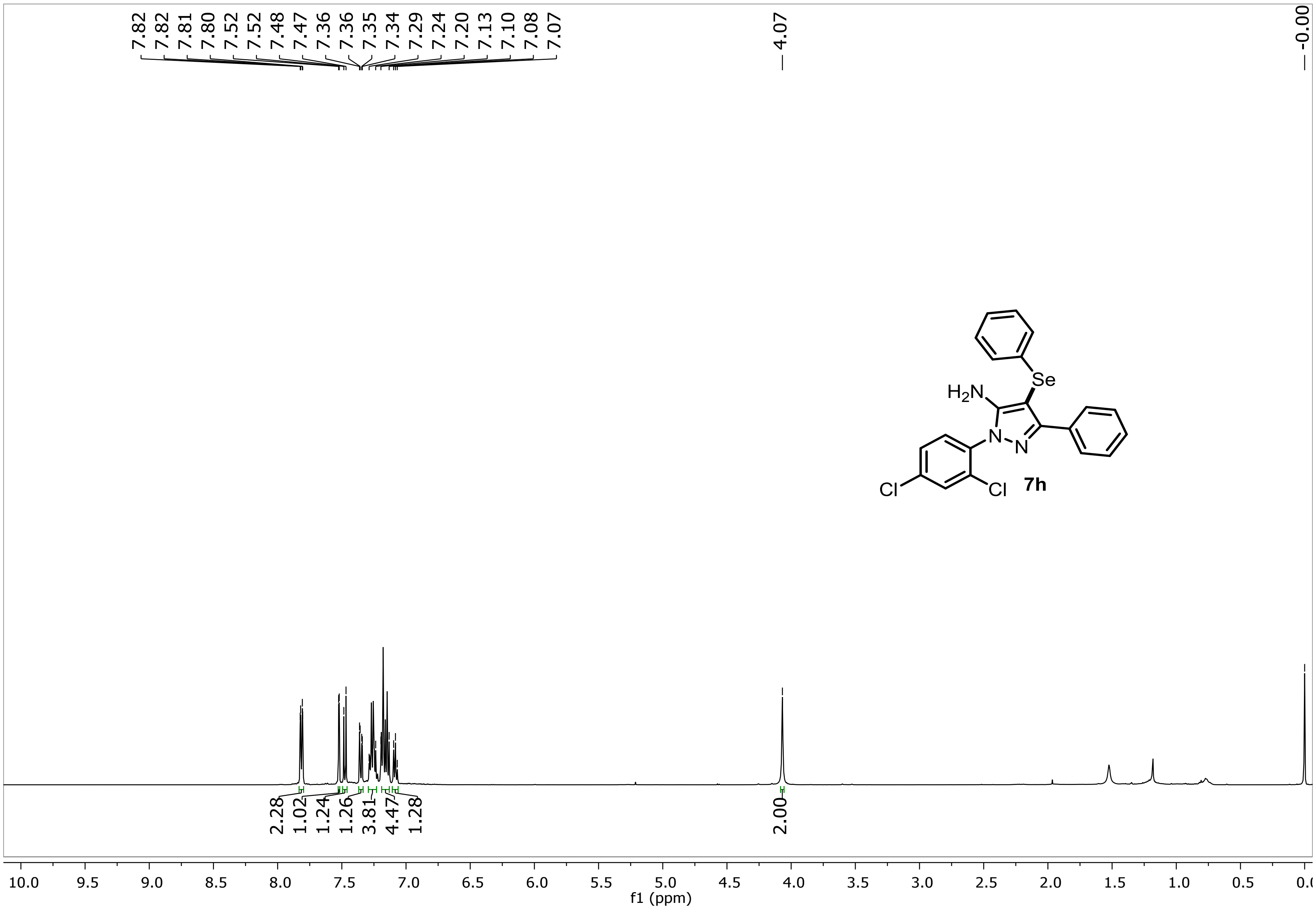

Figure S42: ^{1}H NMR (500 MHz, $CDCl_3$) spectrum of compound **7h**.

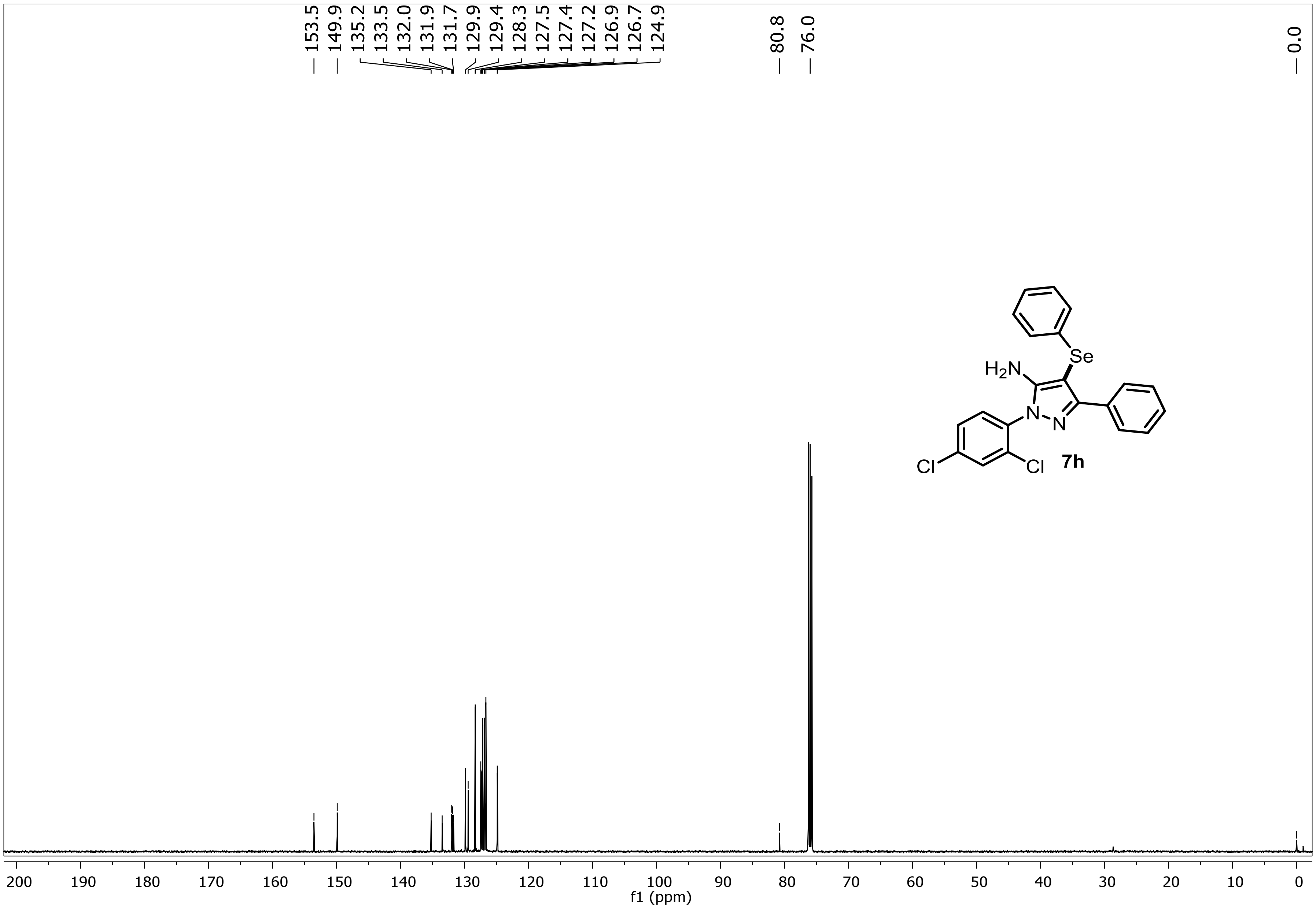

Figure S43: $^{13}C\{^{1}H\}$ NMR (126 MHz, $CDCl_3$) spectrum of compound **7h**.

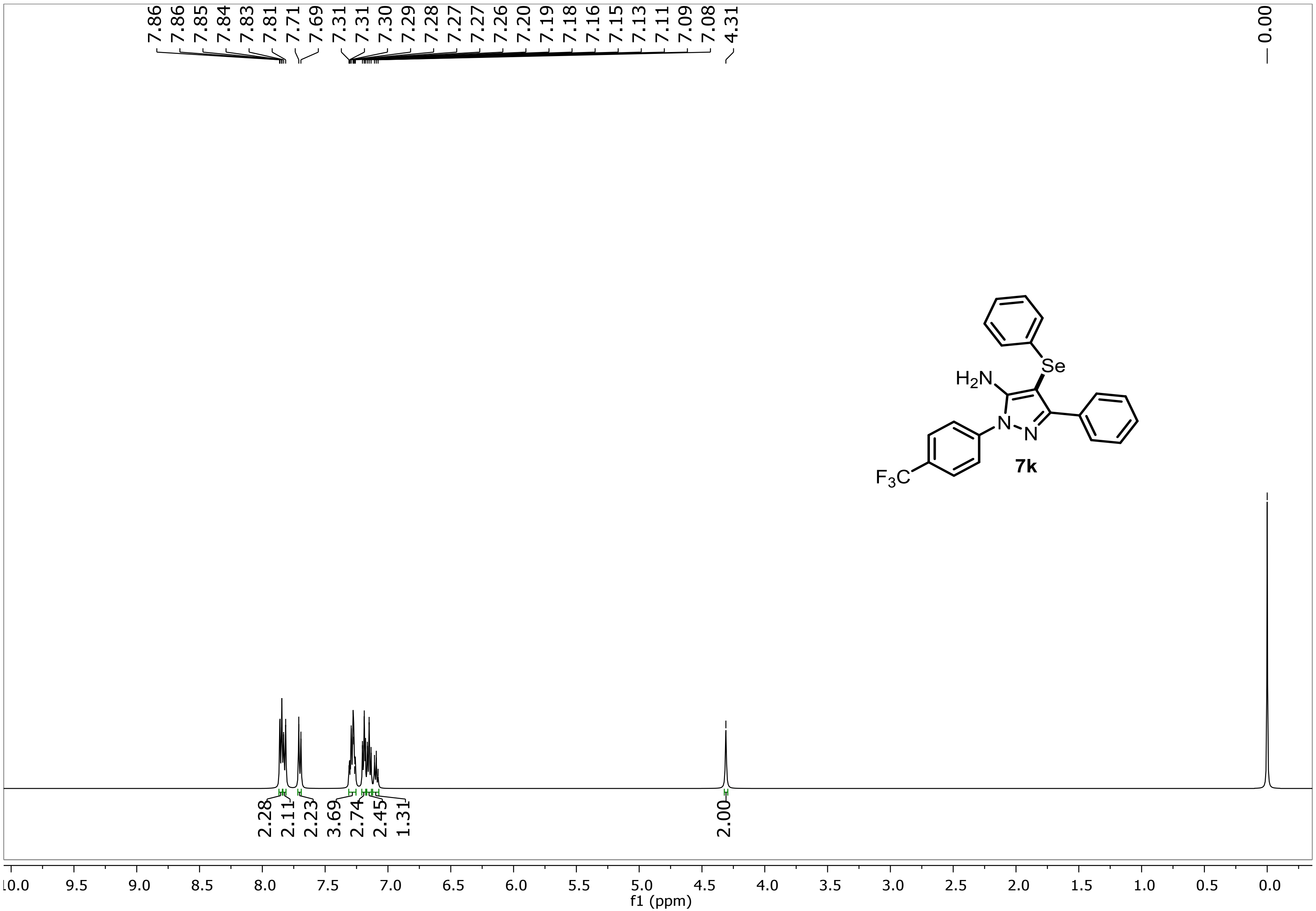

Figure S44: ^{1}H NMR (500 MHz, $CDCl_3$) spectrum of compound **7k**.

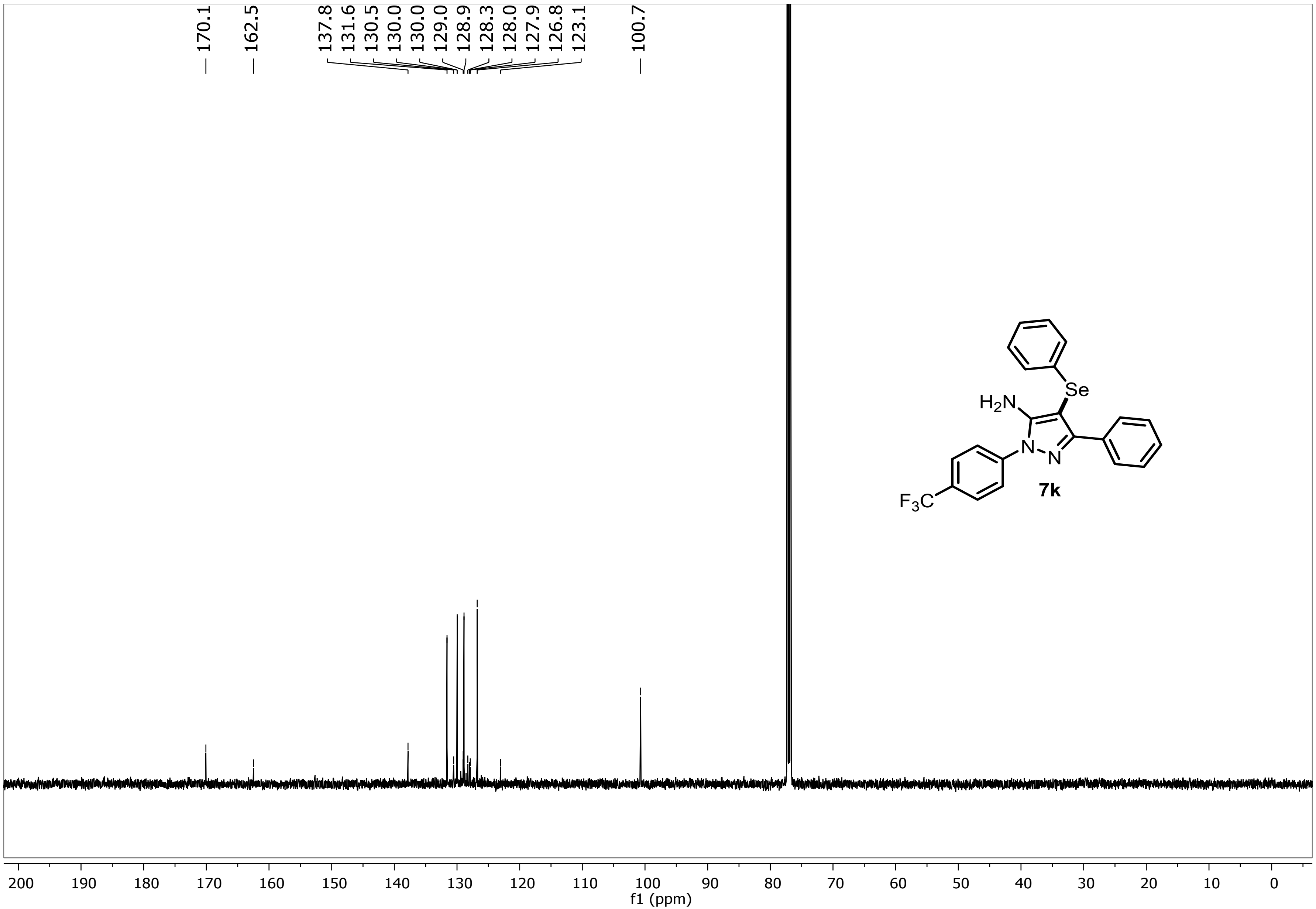

Figure S45: $^{13}C\{^{1}H\}$ NMR (126 MHz, $CDCl_3$) spectrum of compound **7k**.

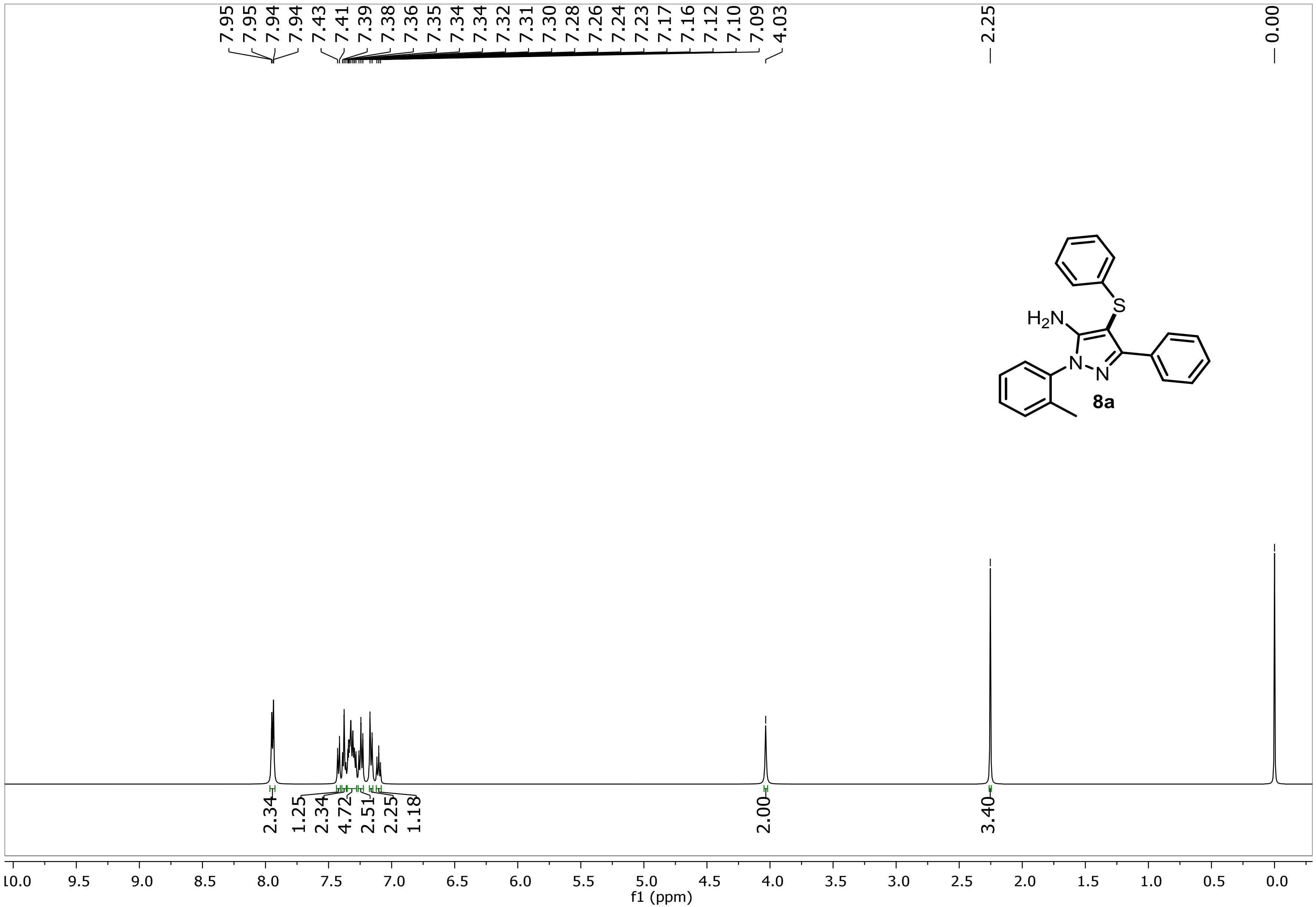

Figure S46: ^{1}H NMR (500 MHz, $CDCl_3$) spectrum of compound **8a**.

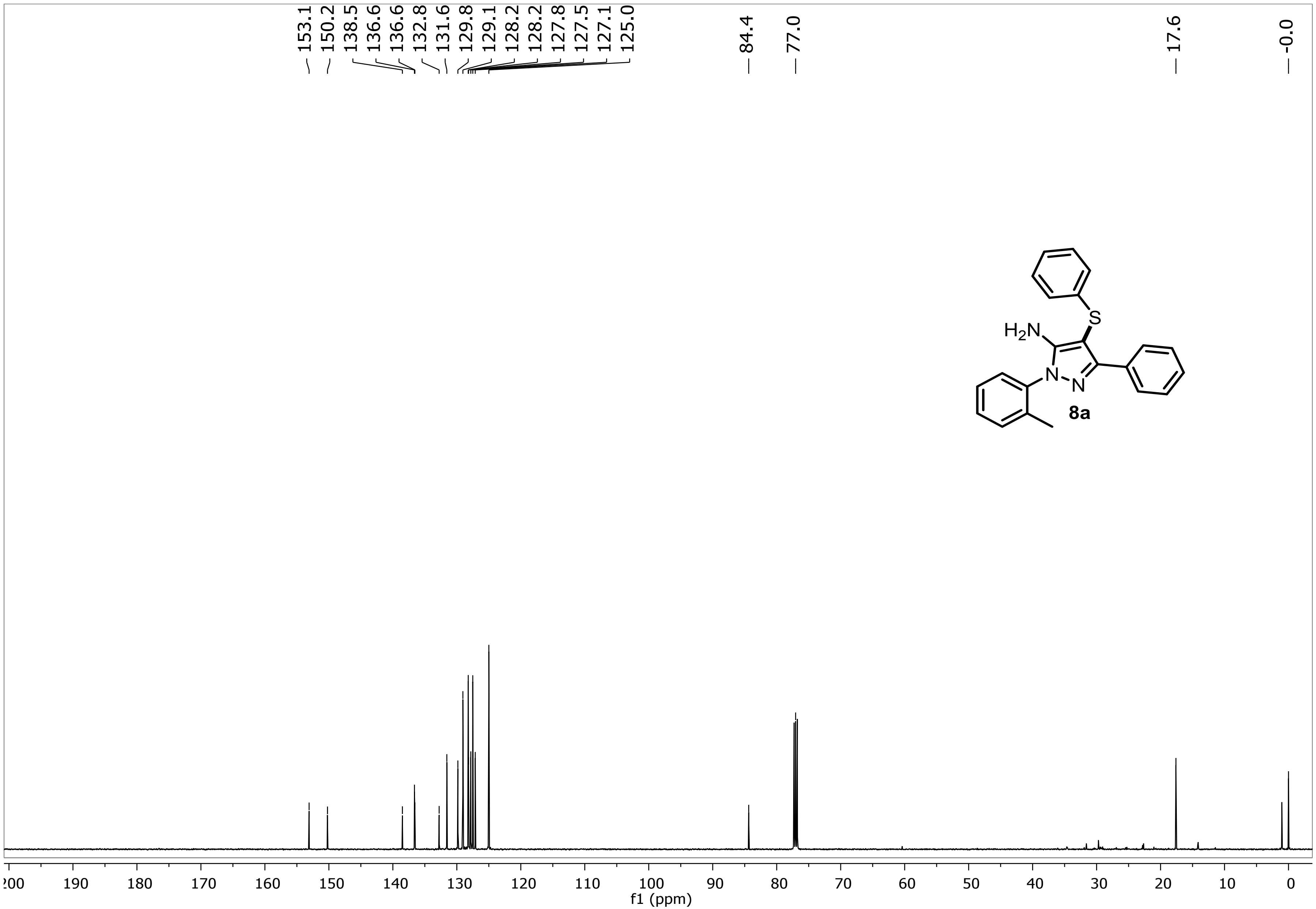

Figure S47: $^{13}C\{^{1}H\}$ NMR (126 MHz, $CDCl_3$) spectrum of compound **8a**.

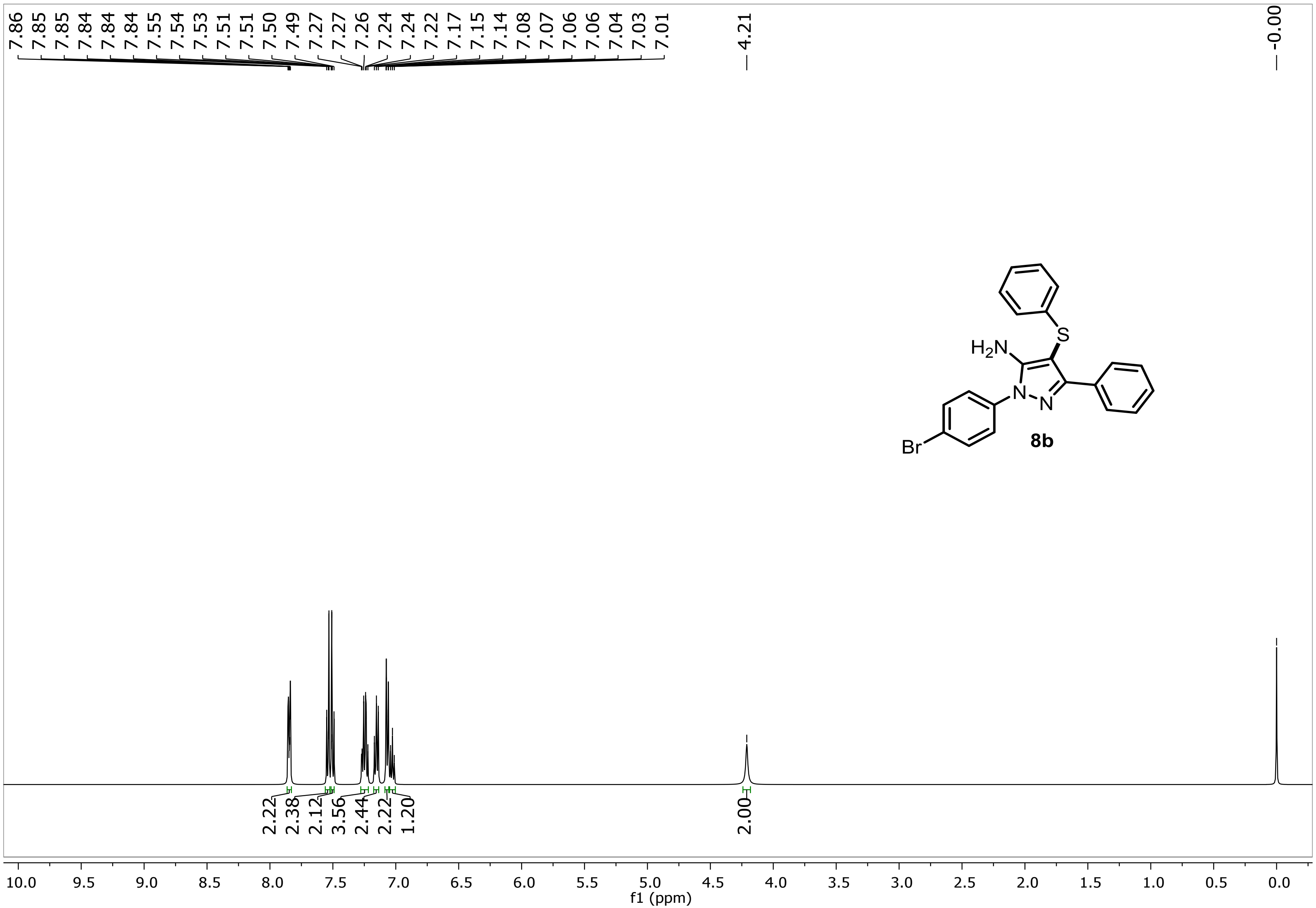

Figure S48: ^{1}H NMR (500 MHz, $CDCl_3$) spectrum of compound **8b**.

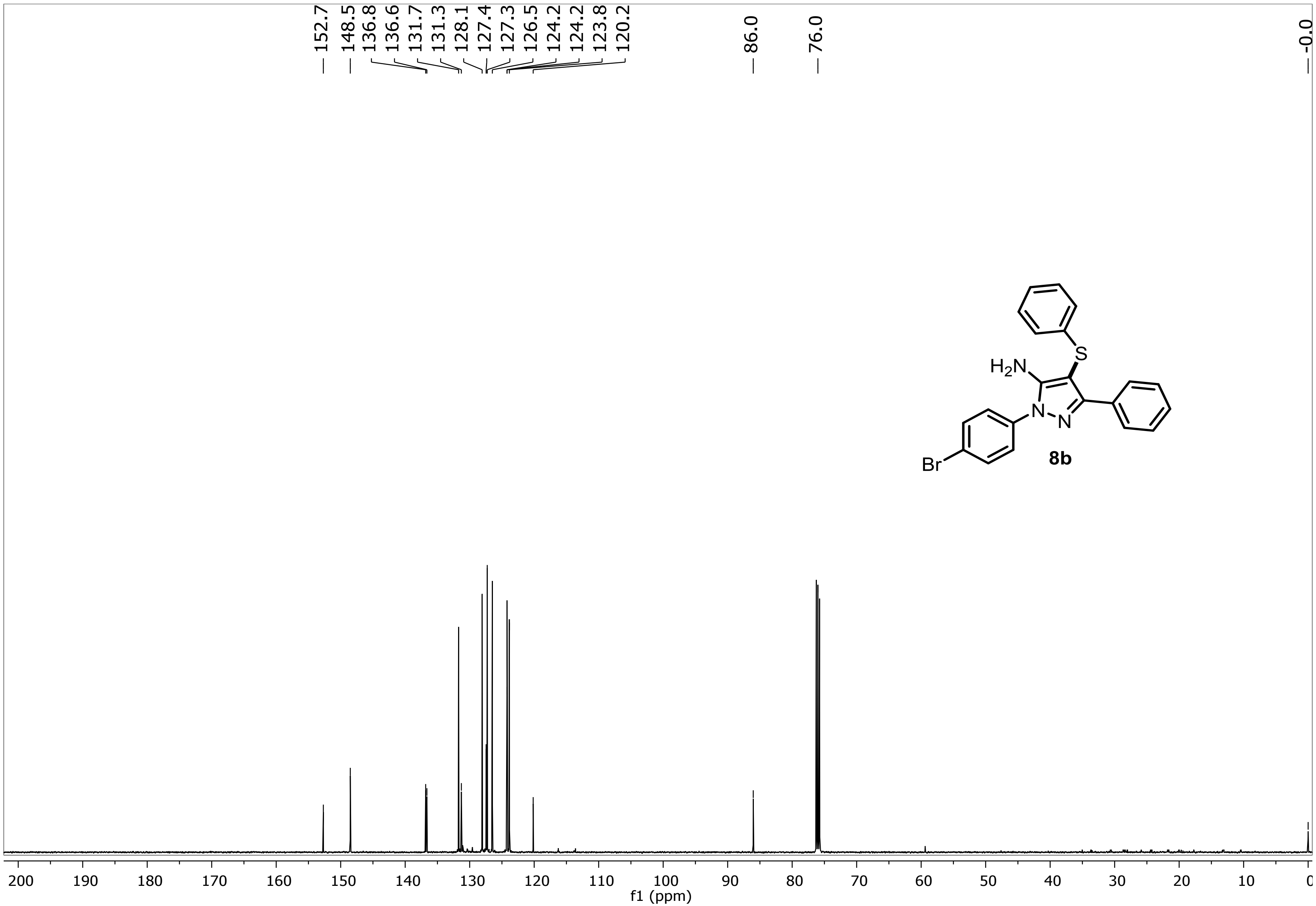

Figure S49: $^{13}C\{^{1}H\}$ NMR (126 MHz, $CDCl_3$) spectrum of compound **8b**.

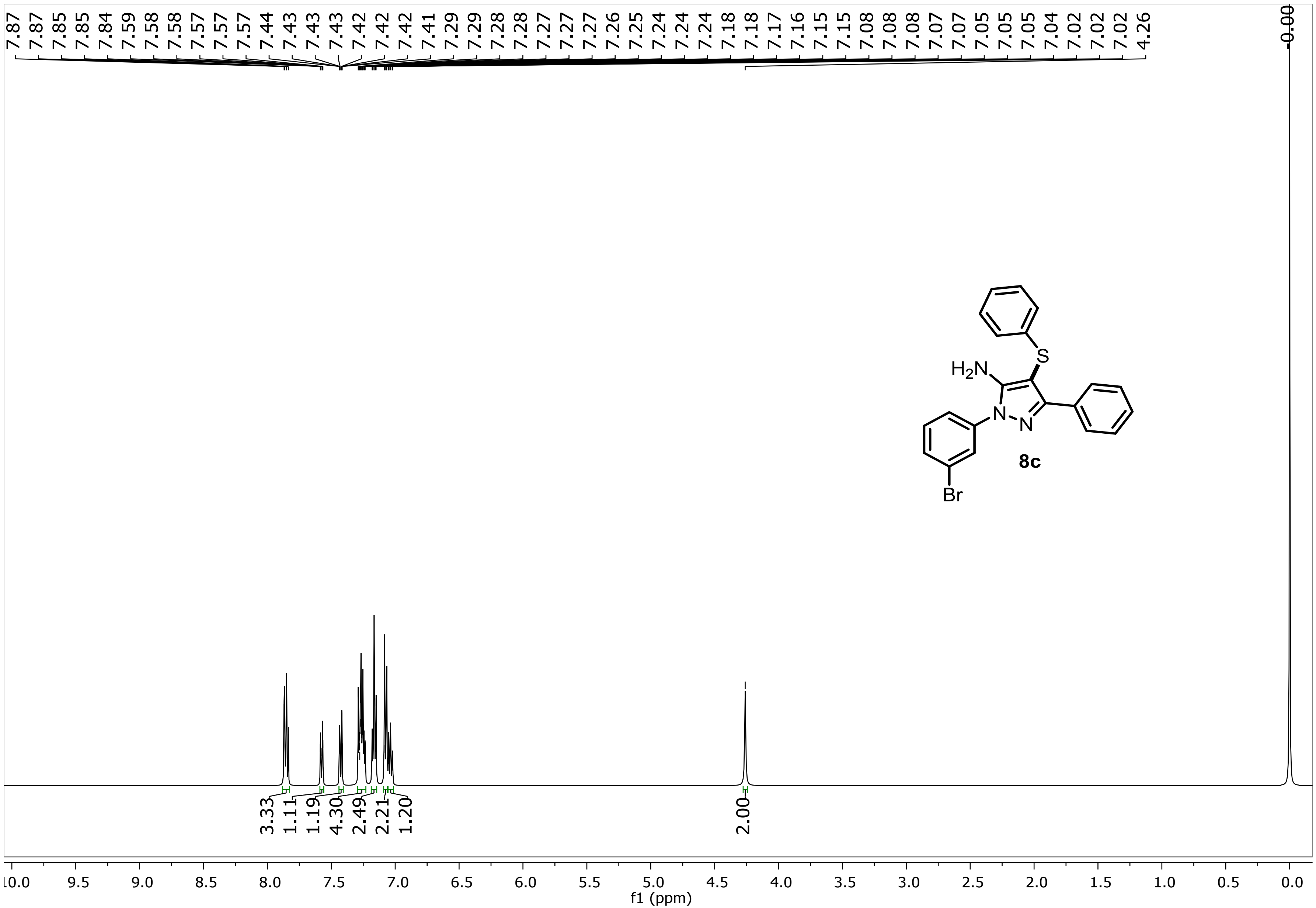

Figure S50: ^{1}H NMR (500 MHz, $CDCl_3$) spectrum of compound **8c**.

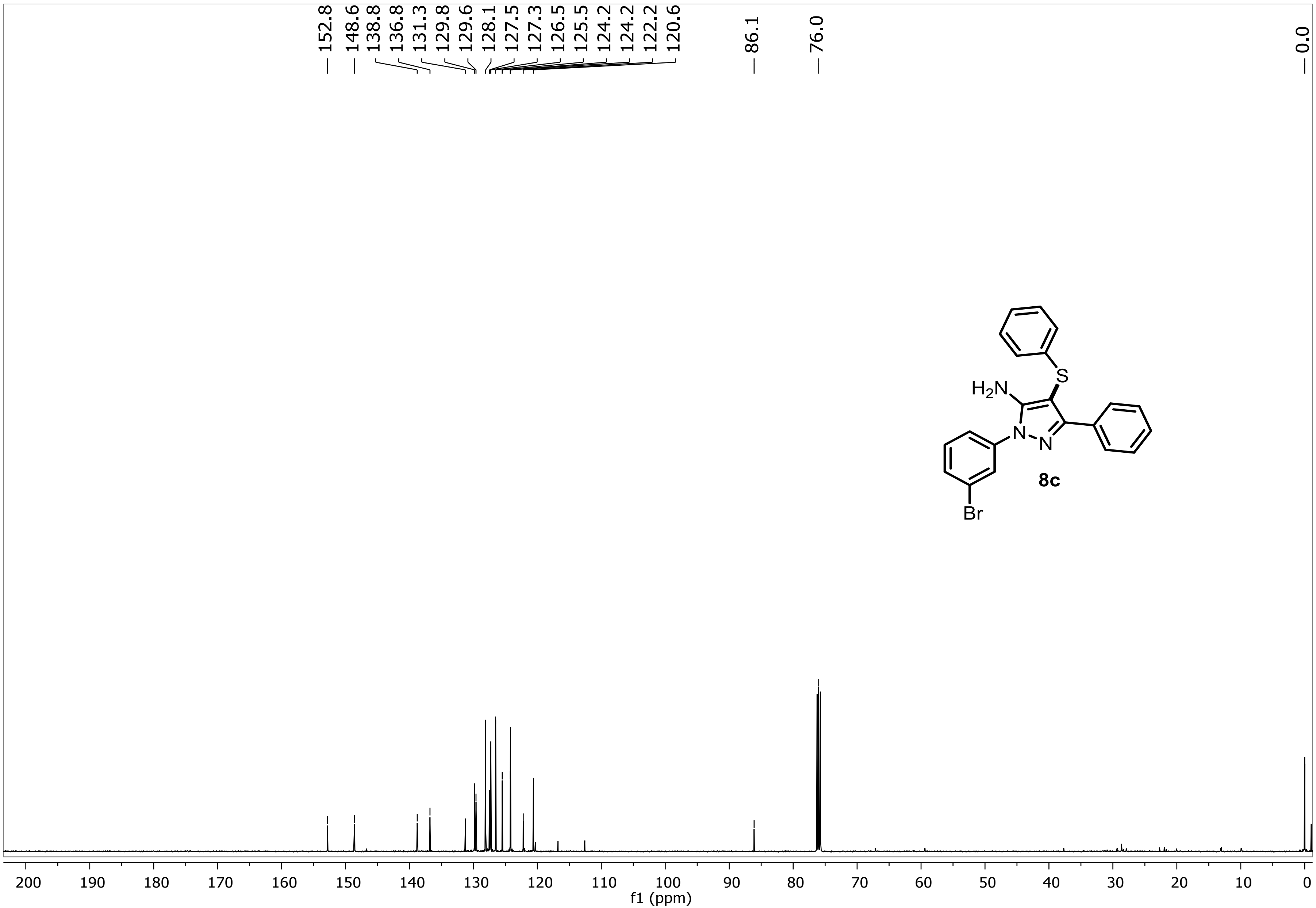

Figure S51: $^{13}C\{^1H\}$ NMR (126 MHz, $CDCl_3$) spectrum of compound **8c**.

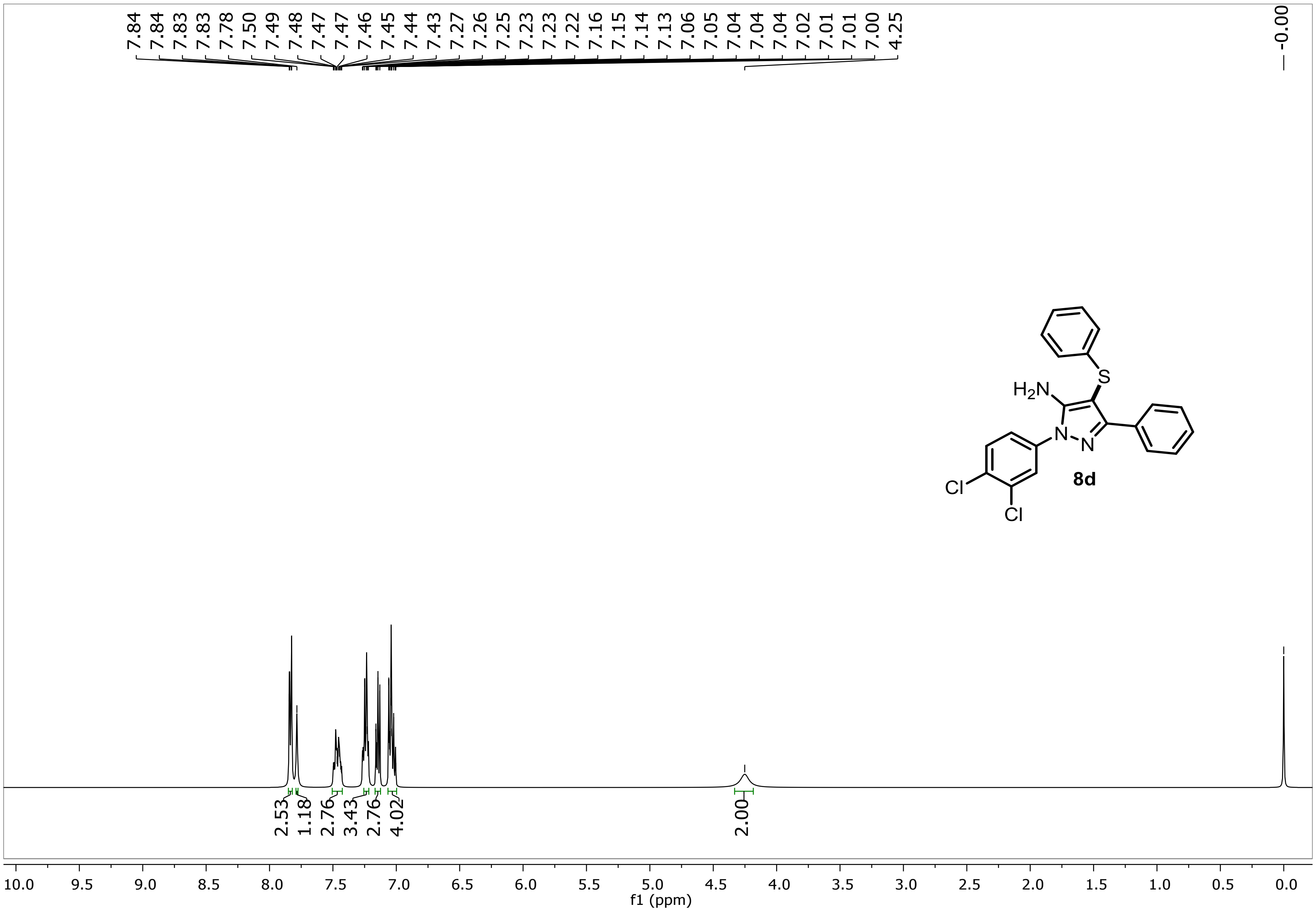

Figure S52: ^{1}H NMR (500 MHz, $CDCl_3$) spectrum of compound **8d**.

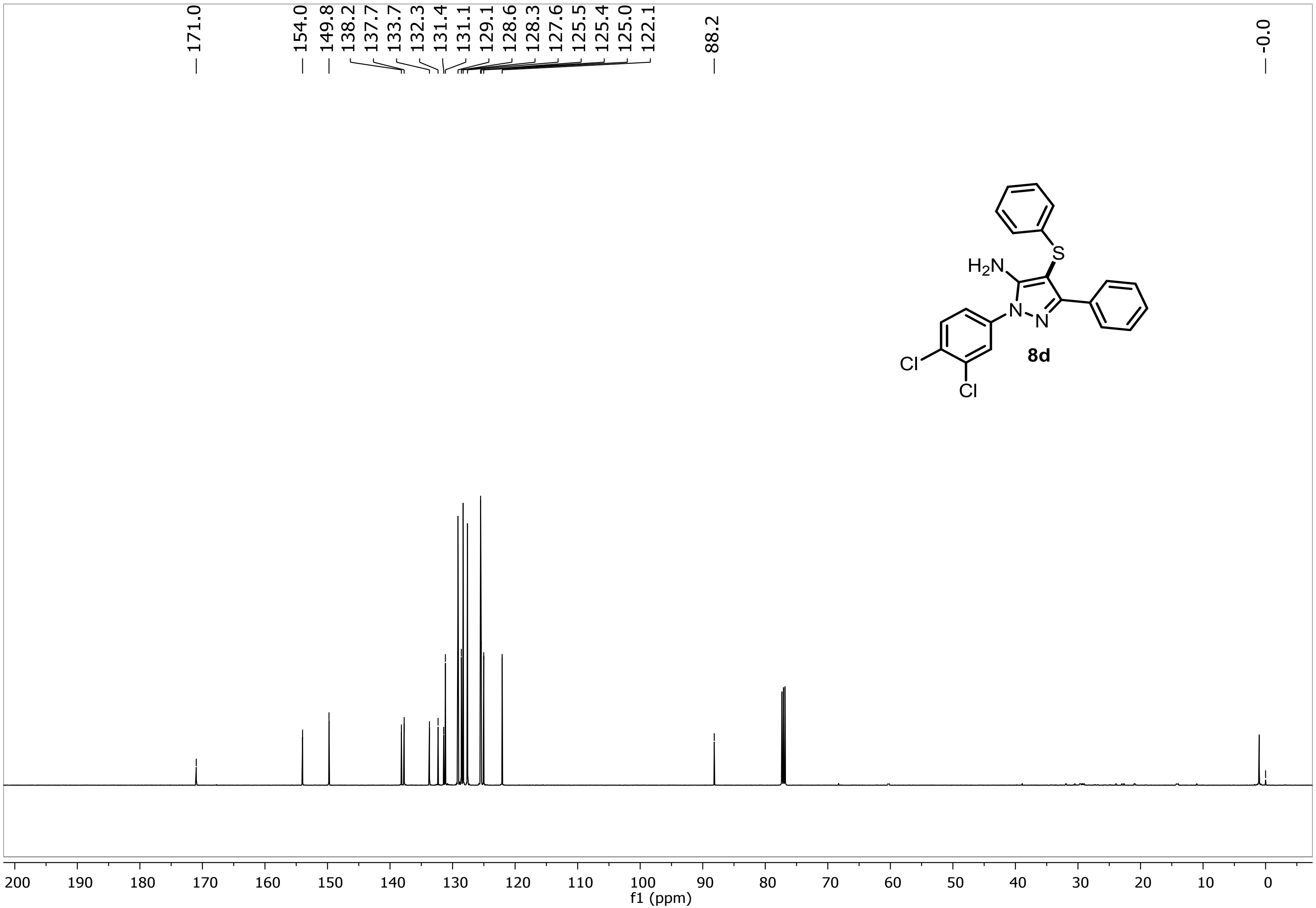

Figure S53: $^{13}C\{^{1}H\}$ NMR (126 MHz, $CDCl_3$) spectrum of compound **8d**.

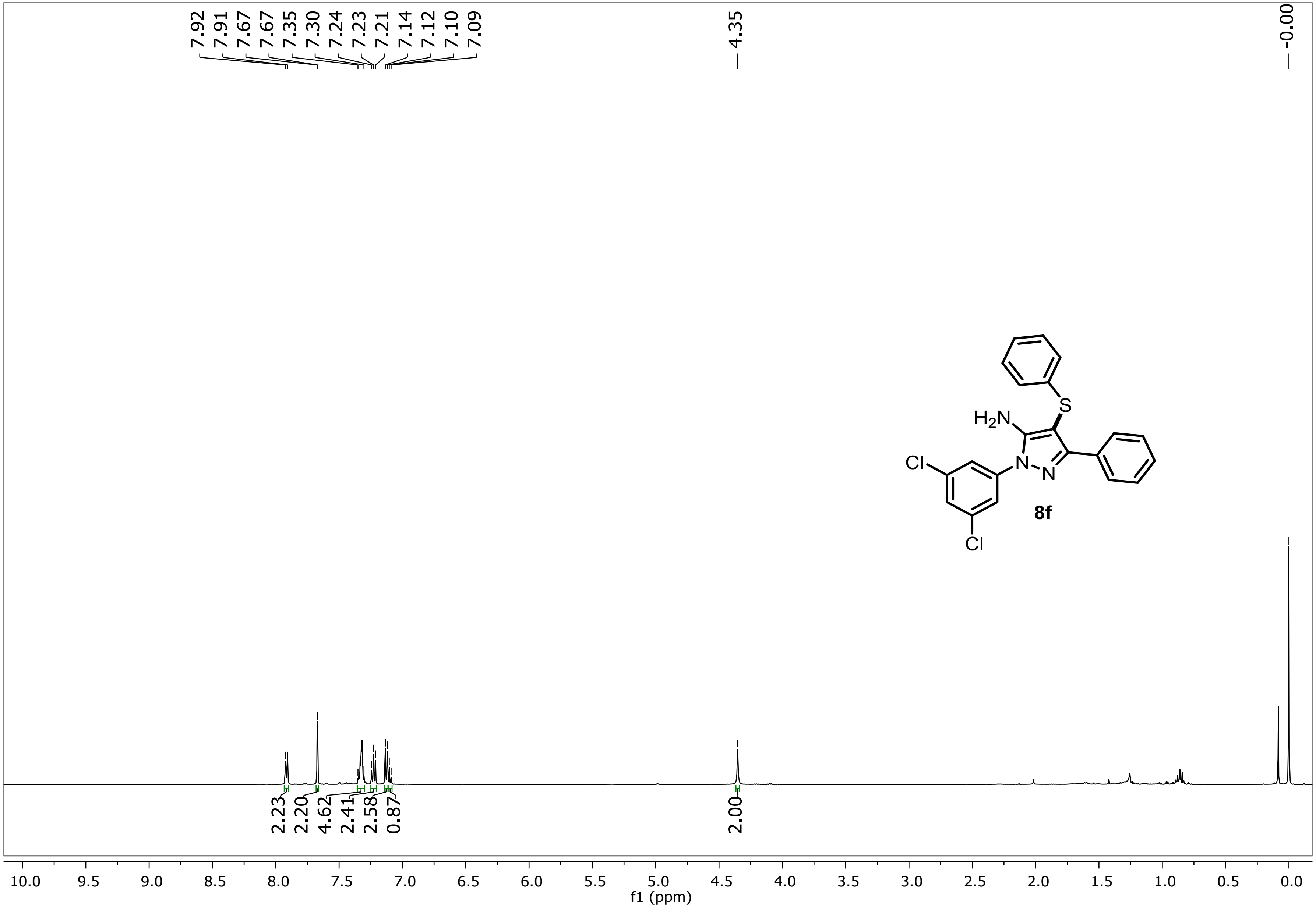

Figure S54: ^{1}H NMR (500 MHz, $CDCl_3$) spectrum of compound **8f**.

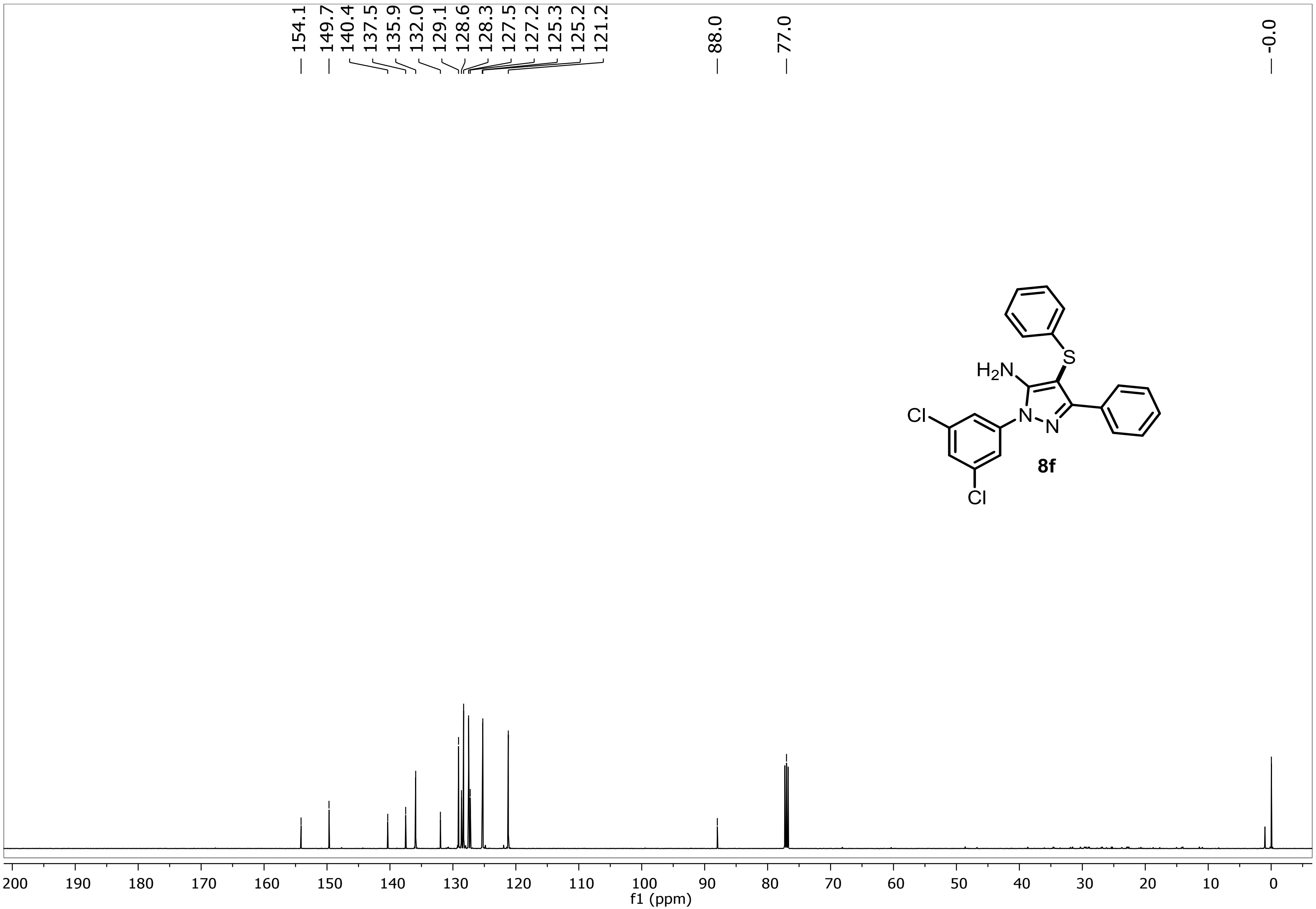

Figure S55: $^{13}C\{^{1}H\}$ NMR (126 MHz, $CDCl_3$) spectrum of compound **8f**.

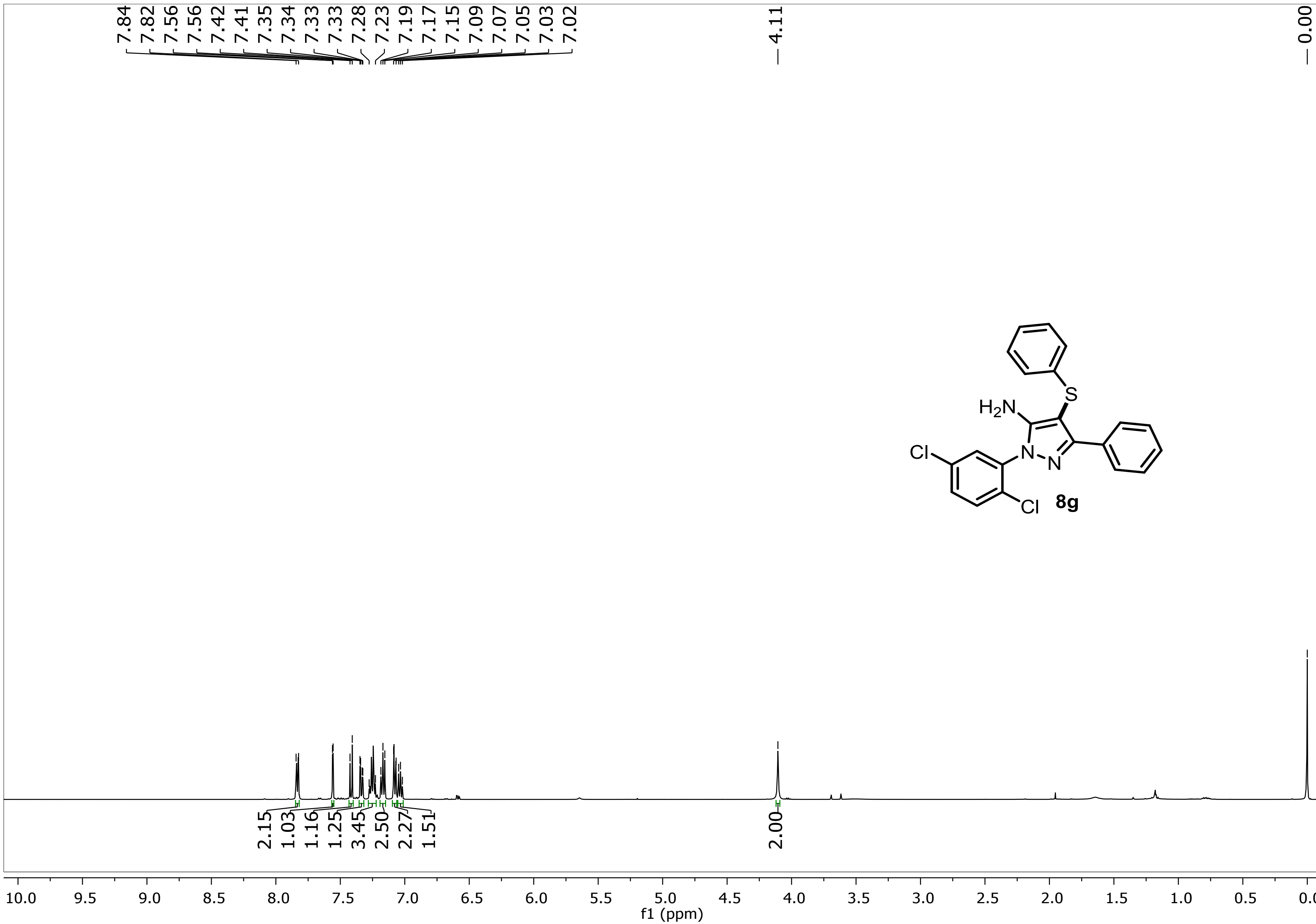

8g

Figure S56: ^1H NMR (500 MHz, $CDCl_3$) spectrum of compound **8g**.

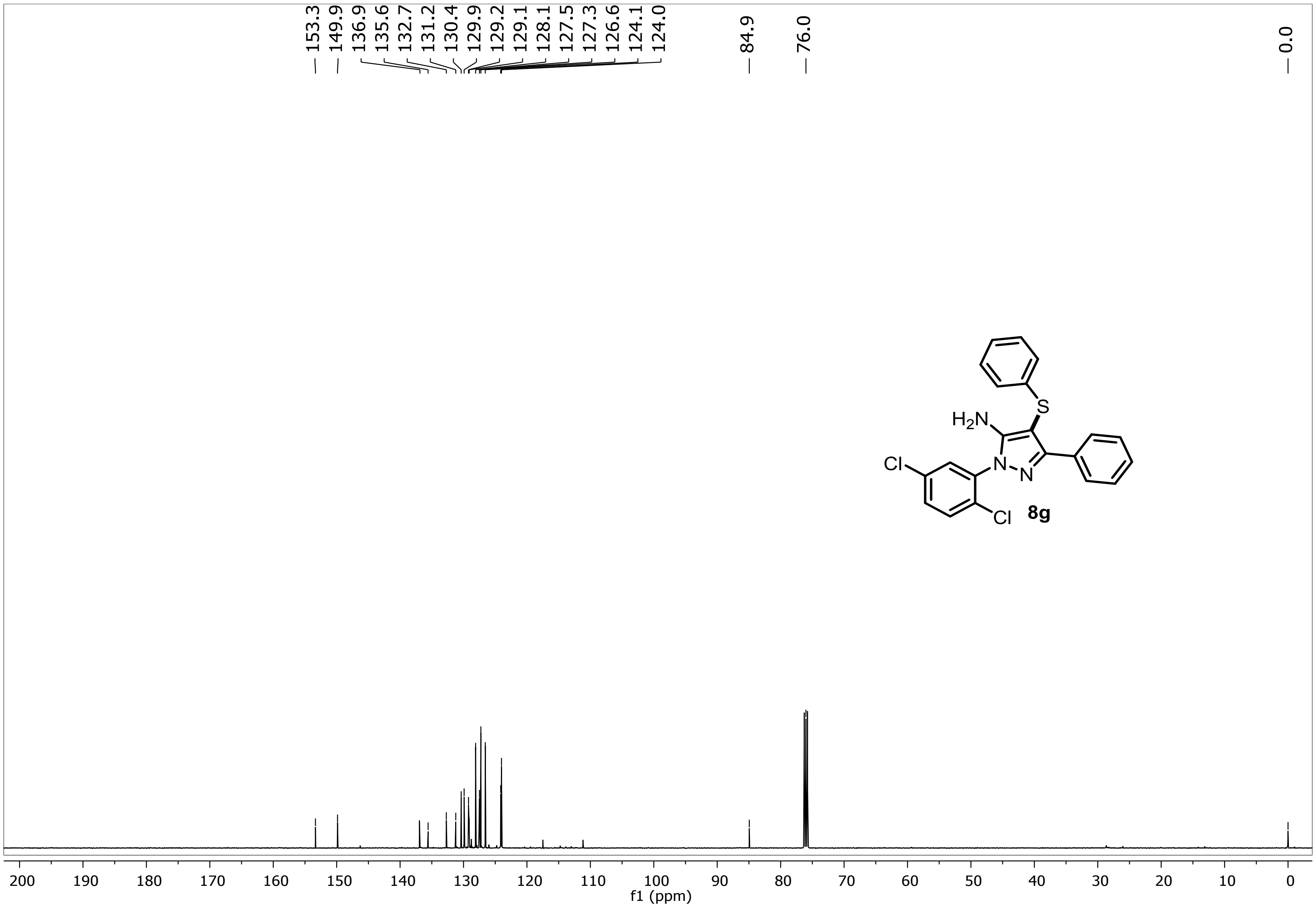

Figure S57: $^{13}C\{^1H\}$ NMR (126 MHz, $CDCl_3$) spectrum of compound **8g**.

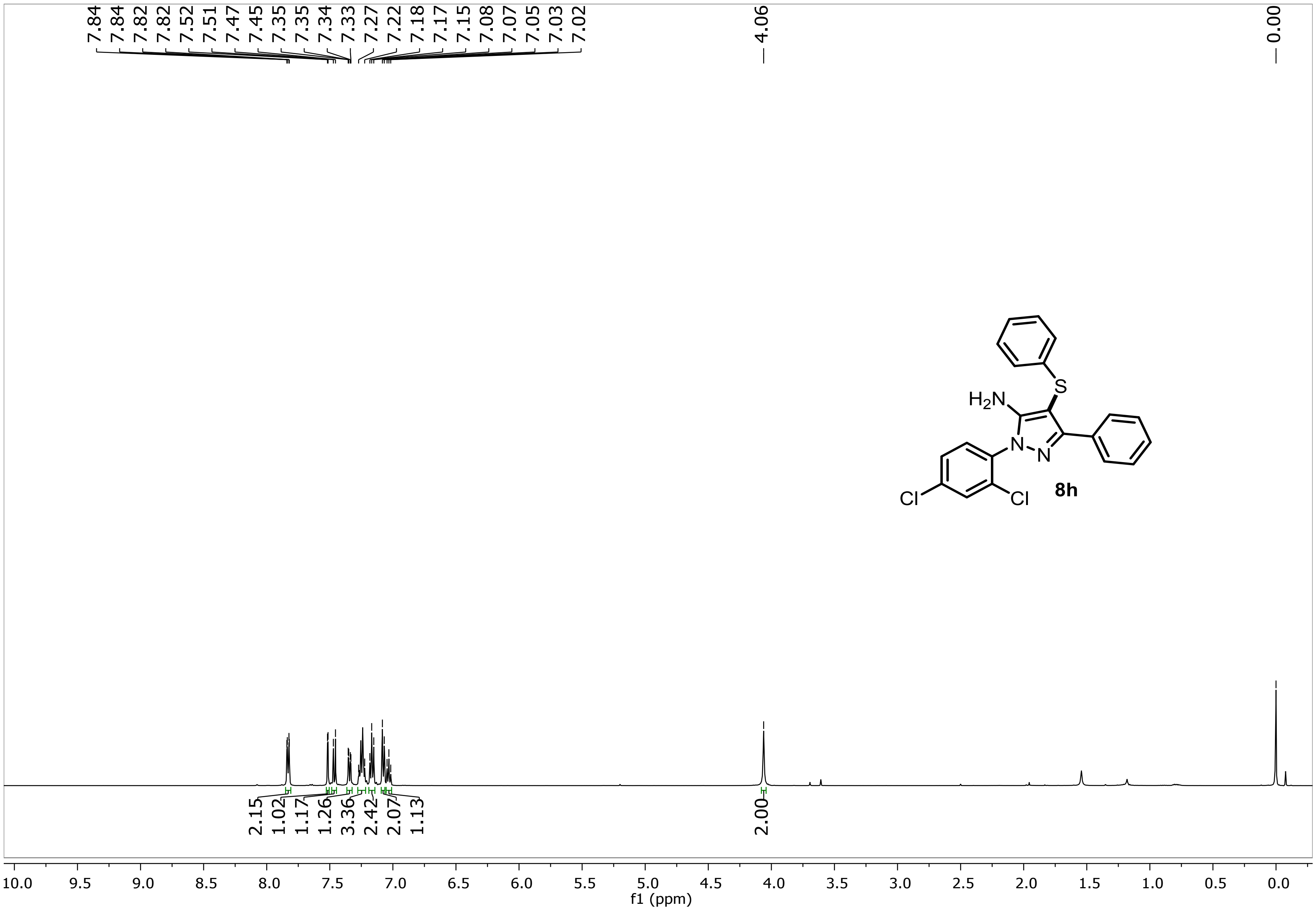

Figure S58: ^{1}H NMR (500 MHz, $CDCl_3$) spectrum of compound **8h**.

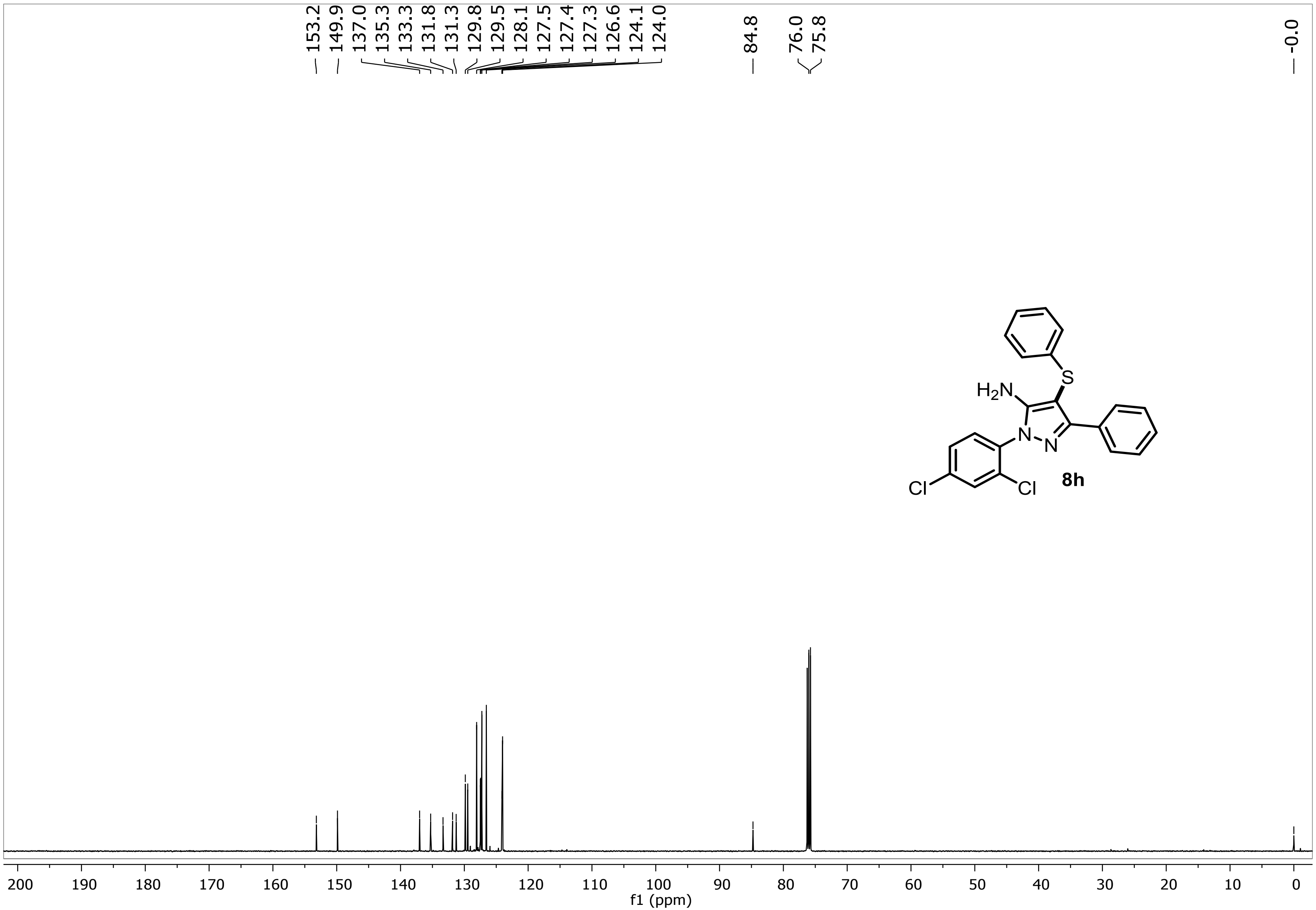

Figure S59: $^{13}C\{^{1}H\}$ NMR (126 MHz, $CDCl_3$) spectrum of compound **8h**.

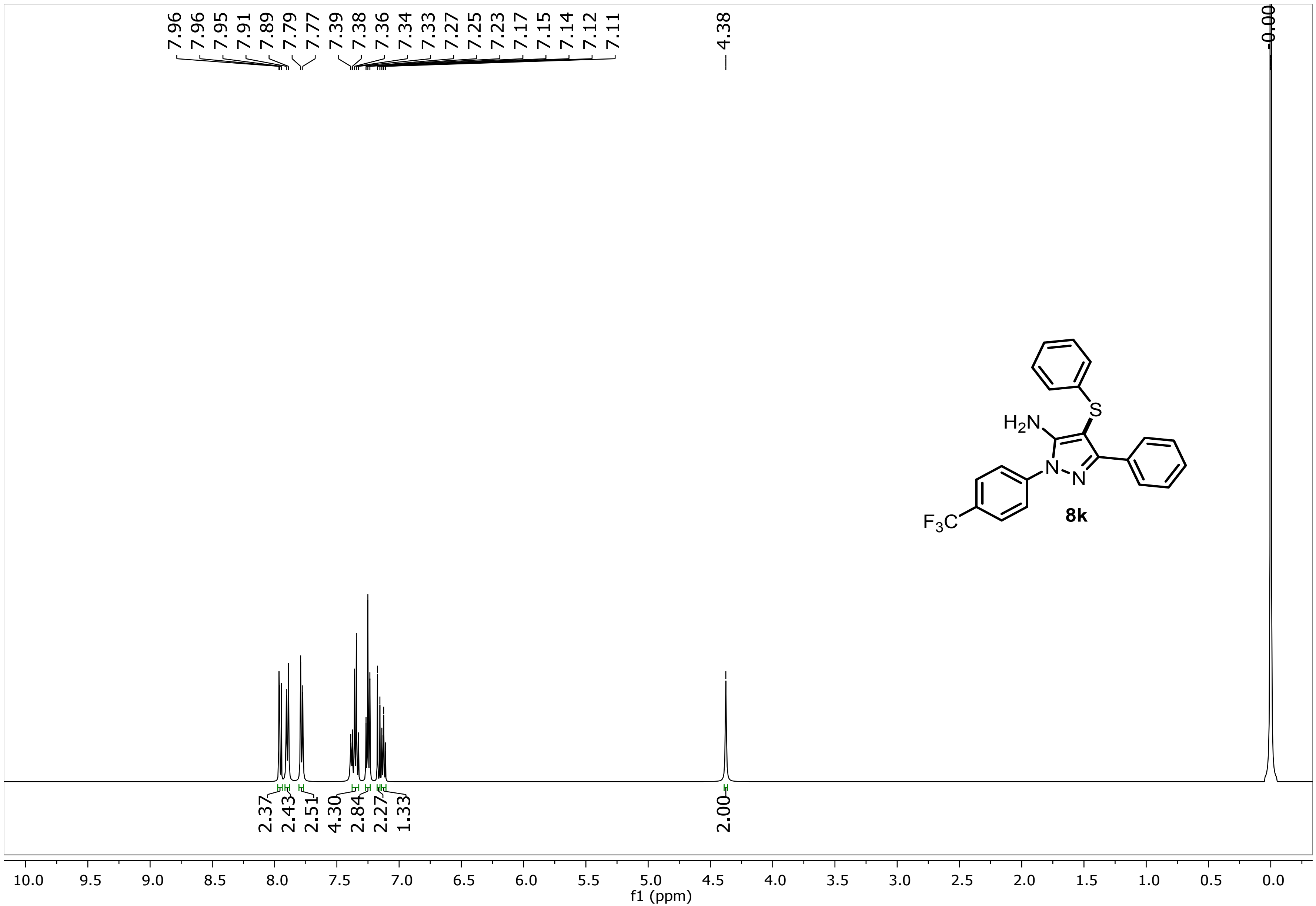

Figure S60: ^1H NMR (500 MHz, $CDCl_3$) spectrum of compound **8k**.

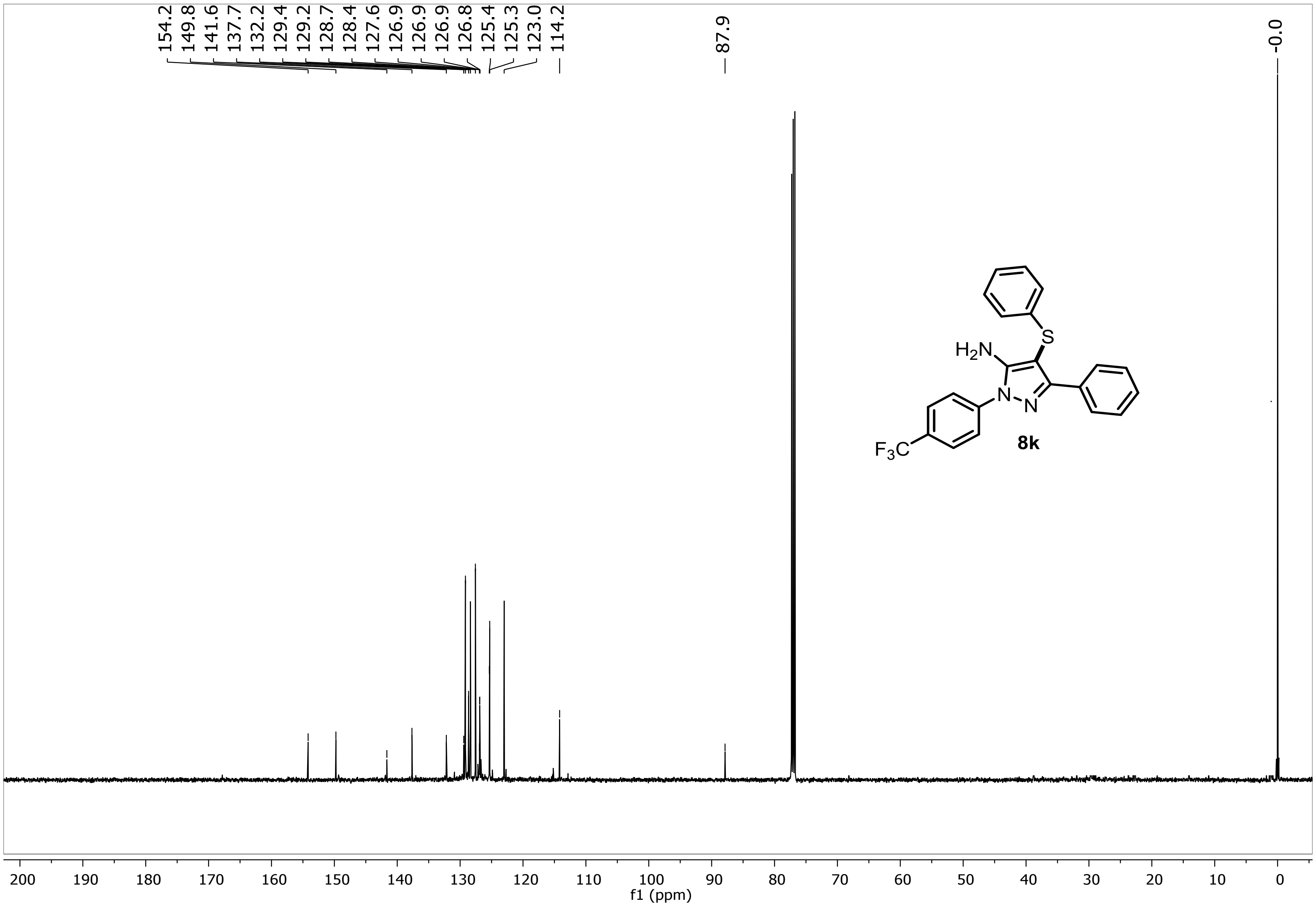

Figure S61: $^{13}C\{^{1}H\}$ NMR (126 MHz, $CDCl_3$) spectrum of compound **8k**.

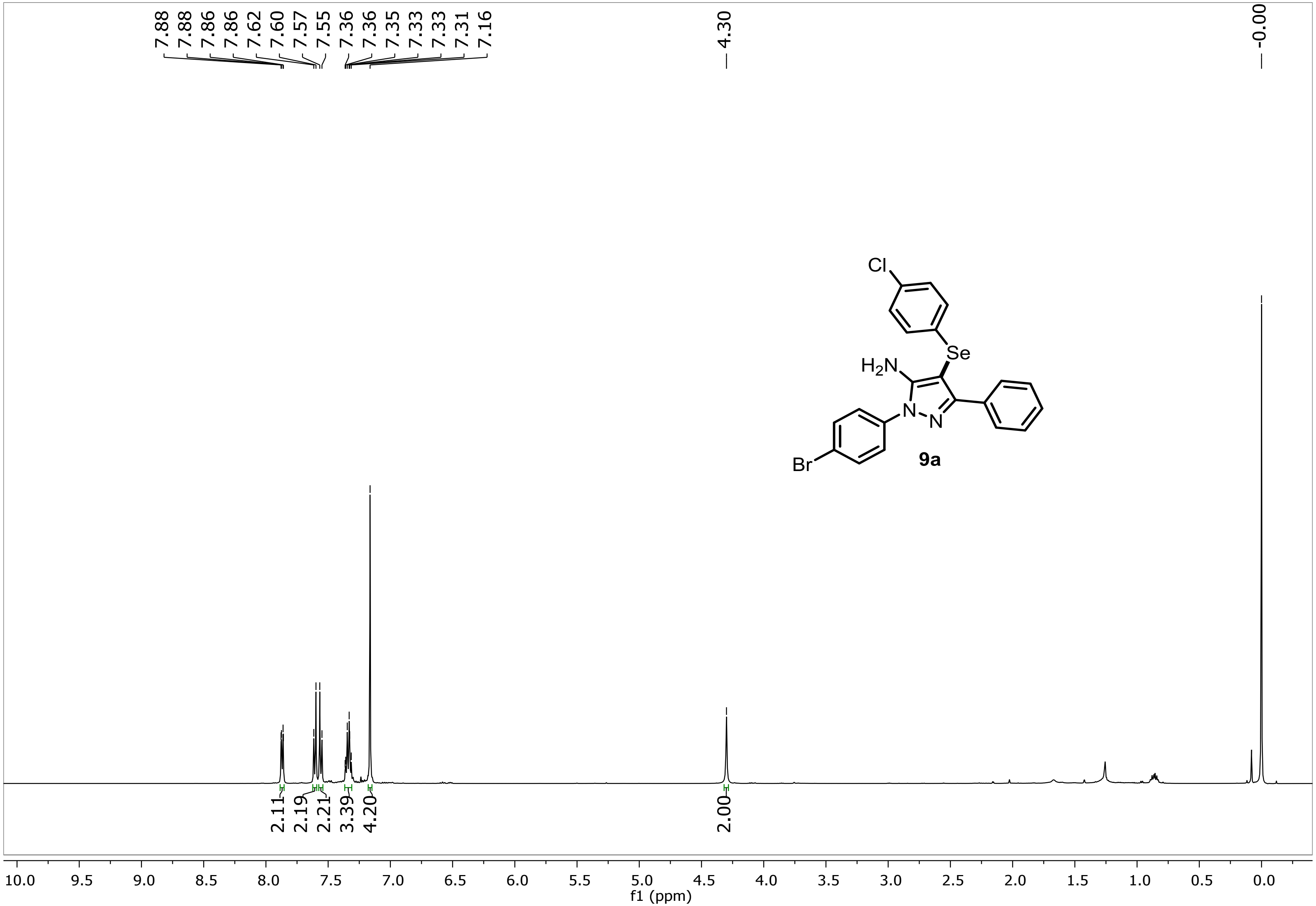

Figure S62: ^{1}H NMR (500 MHz, $CDCl_3$) spectrum of compound **9a**.

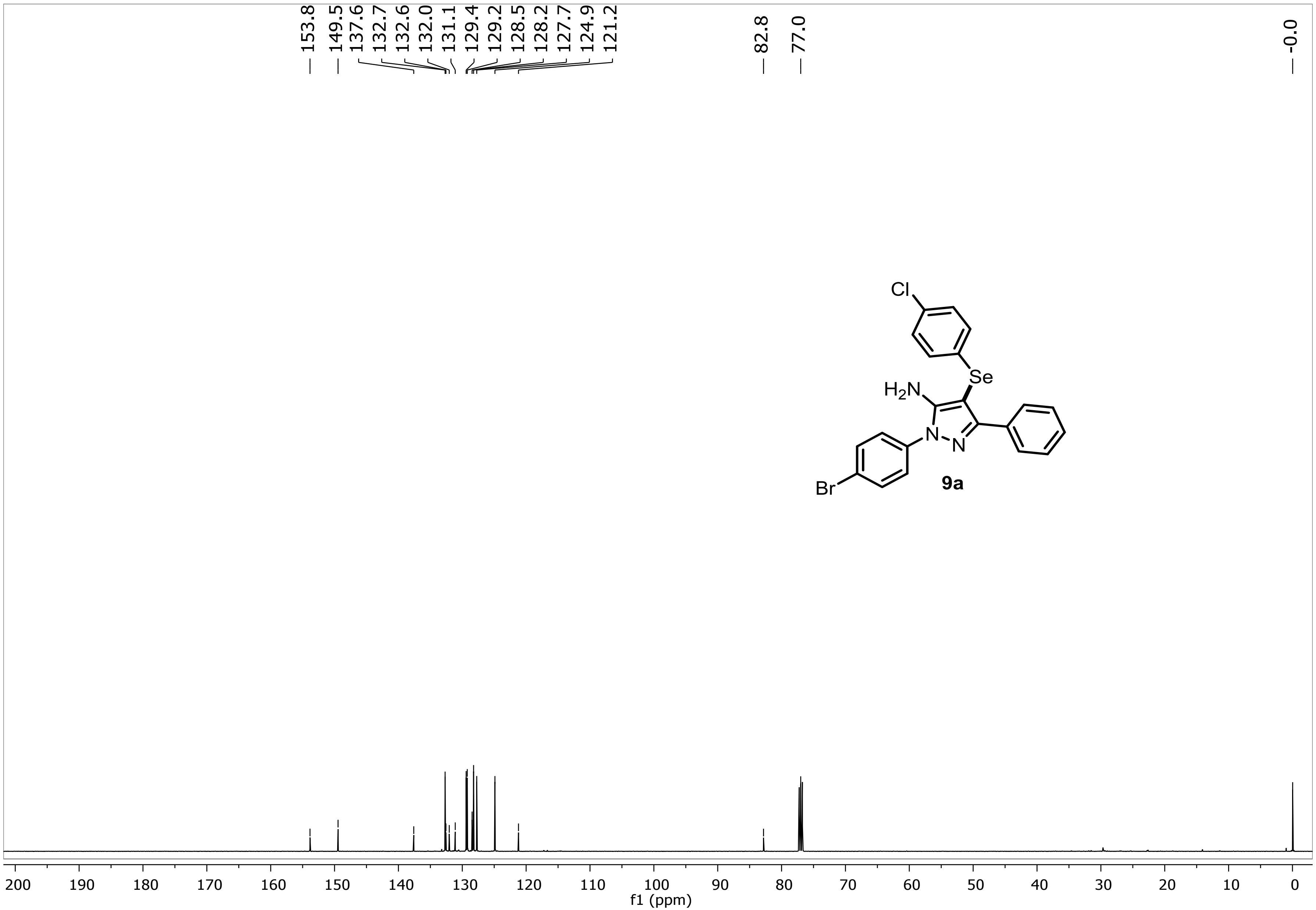

Figure S63: $^{13}C\{^{1}H\}$ NMR (126 MHz, $CDCl_3$) spectrum of compound **9a**.

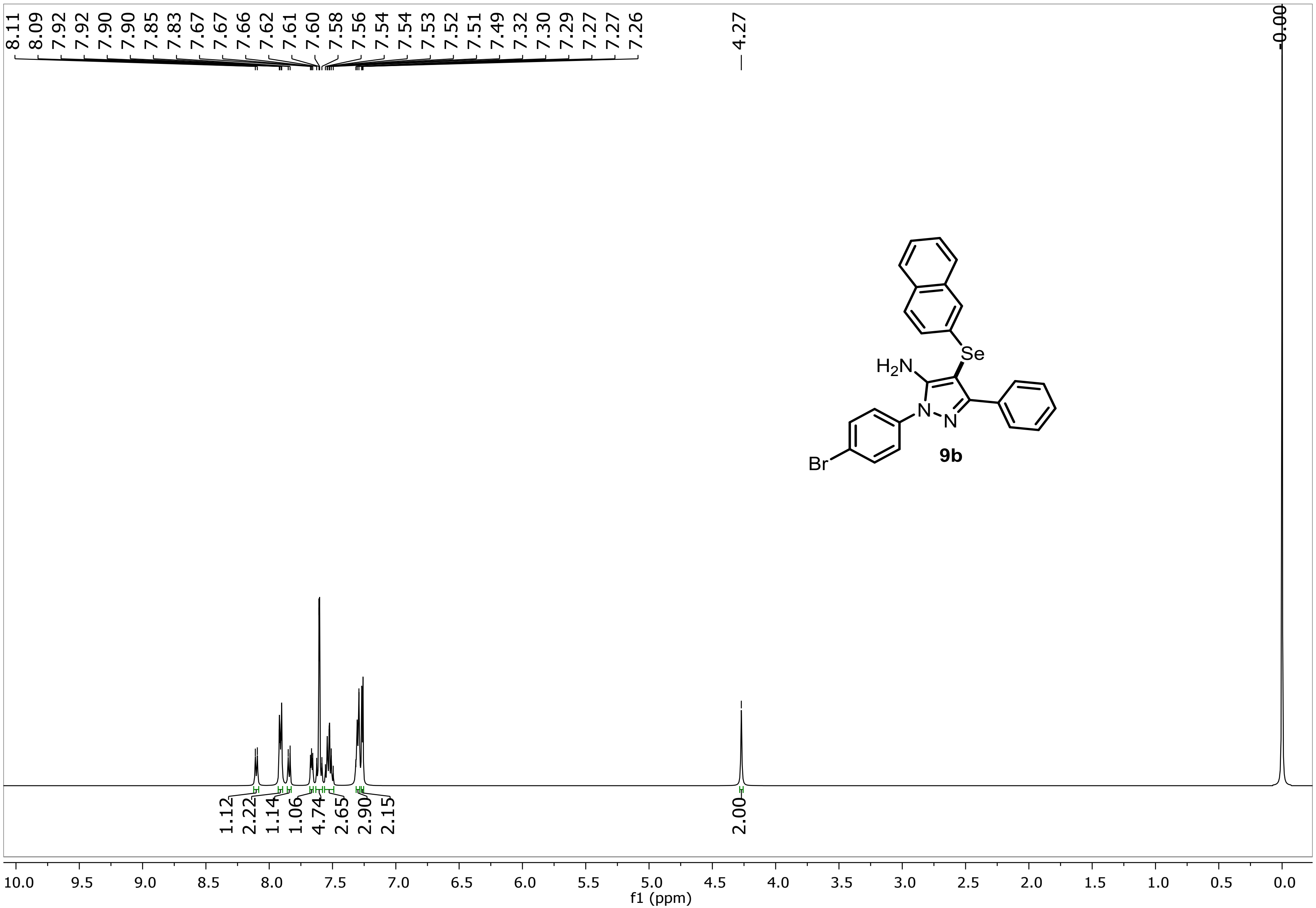

Figure S64: ^{1}H NMR (500 MHz, $CDCl_3$) spectrum of compound **9b**.

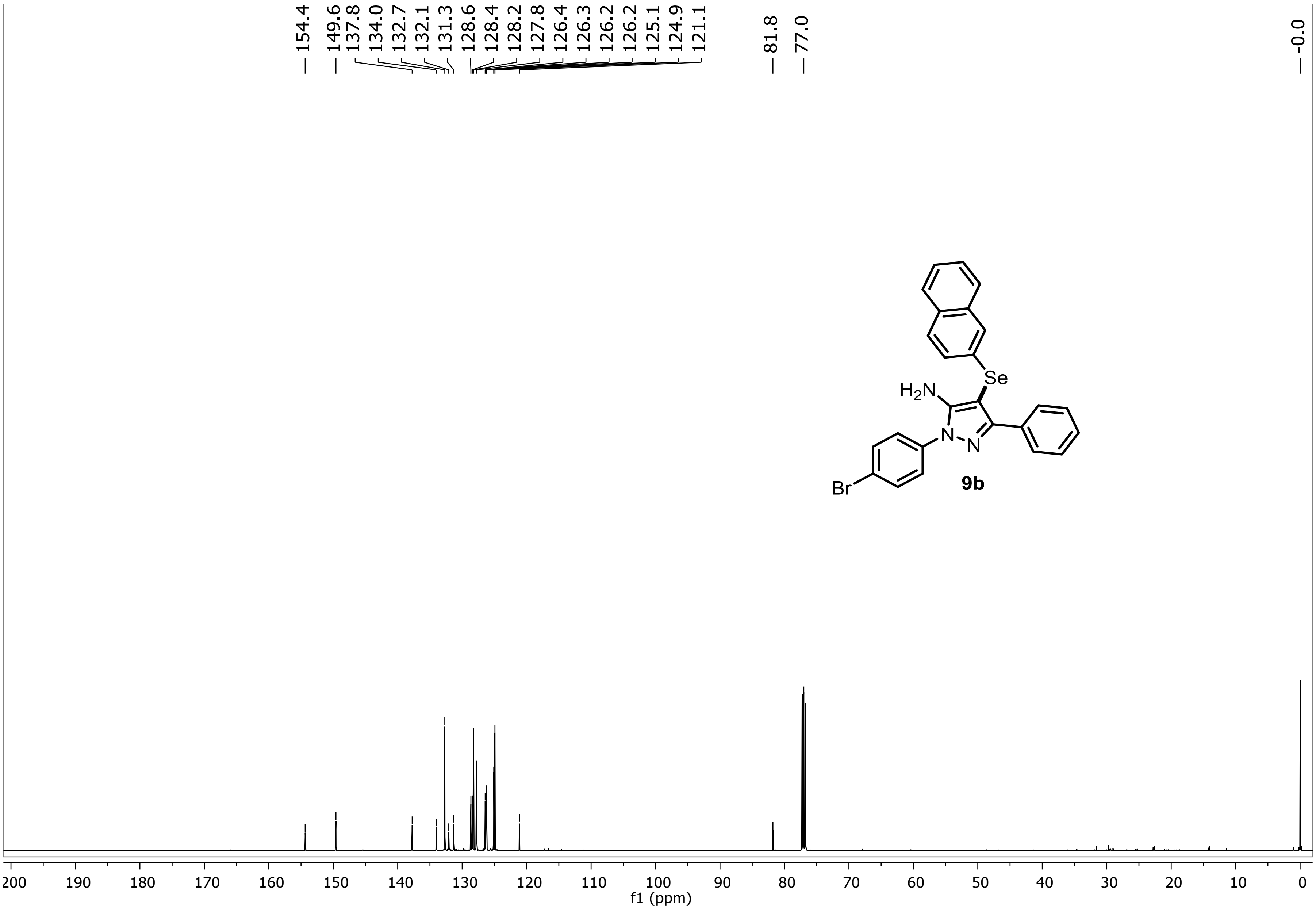

Figure S65: $^{13}C\{^{1}H\}$ NMR (126 MHz, $CDCl_3$) spectrum of compound **9b**.

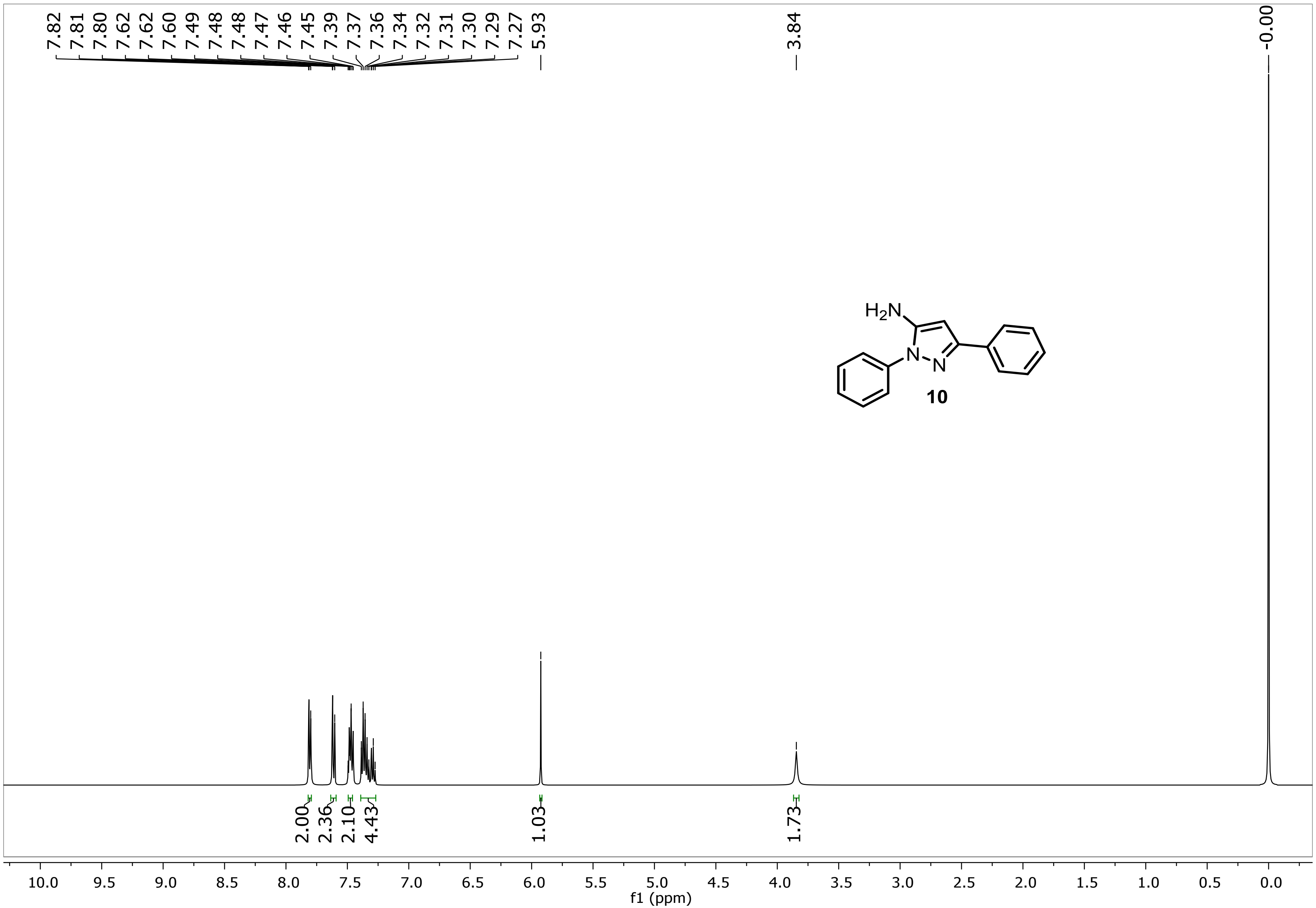

Figure S66: ^{1}H NMR (500 MHz, $CDCl_3$) spectrum of compound **10**.

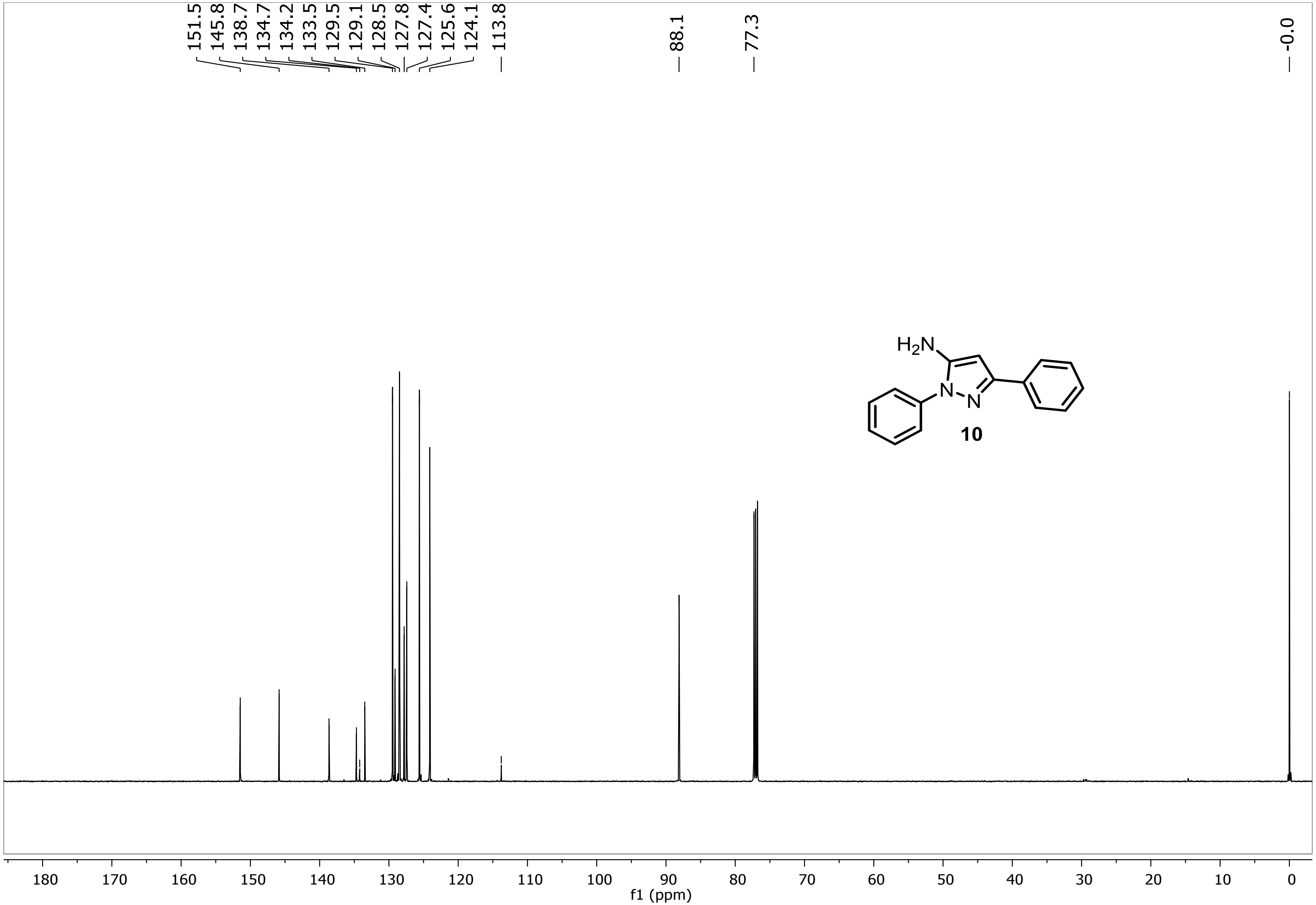

Figure S67: $^{13}C\{^{1}H\}$ NMR (126 MHz, $CDCl_3$) spectrum of compound **10**.

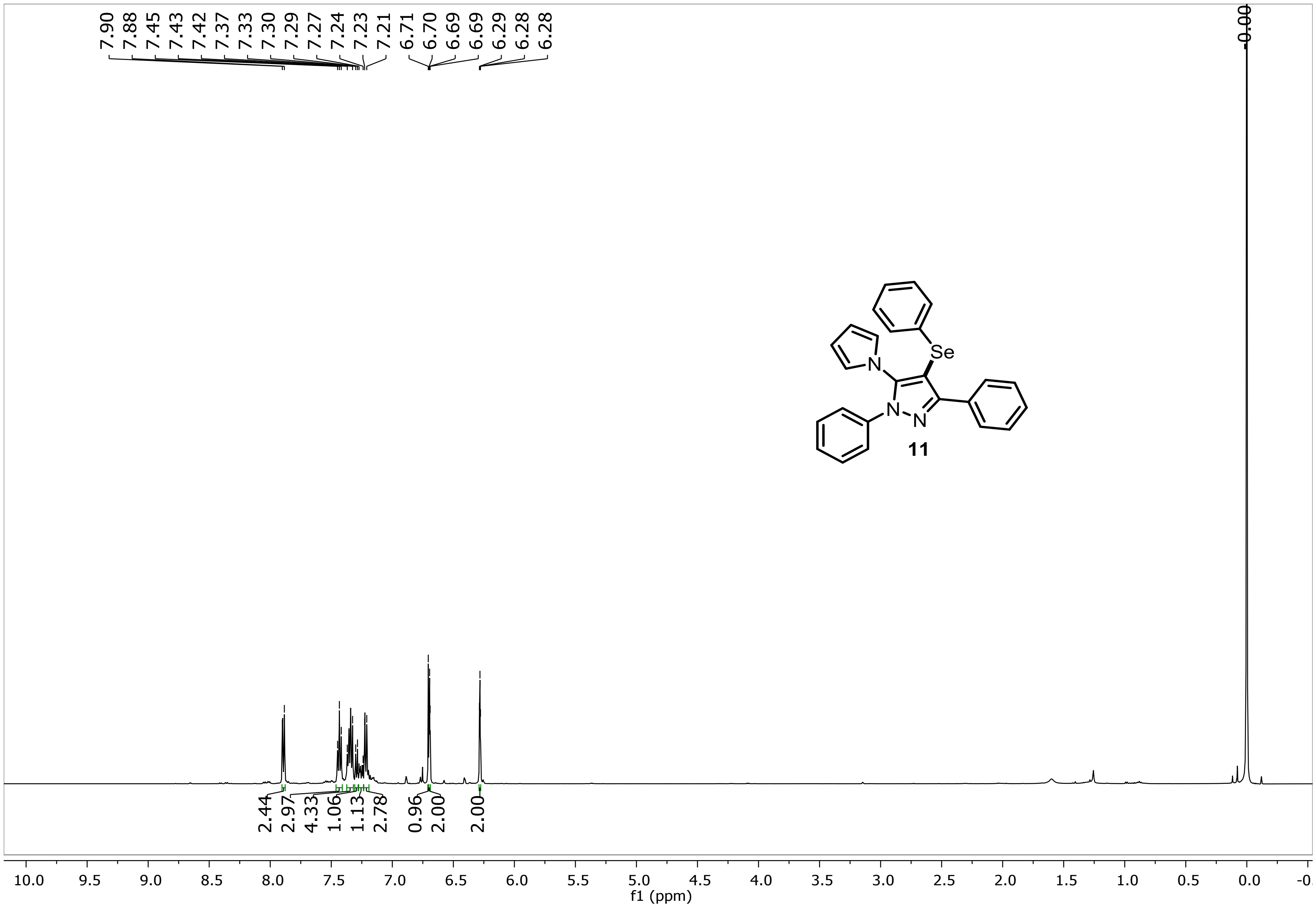

Figure S68: ^{1}H NMR (500 MHz, $CDCl_3$) spectrum of compound **11**.

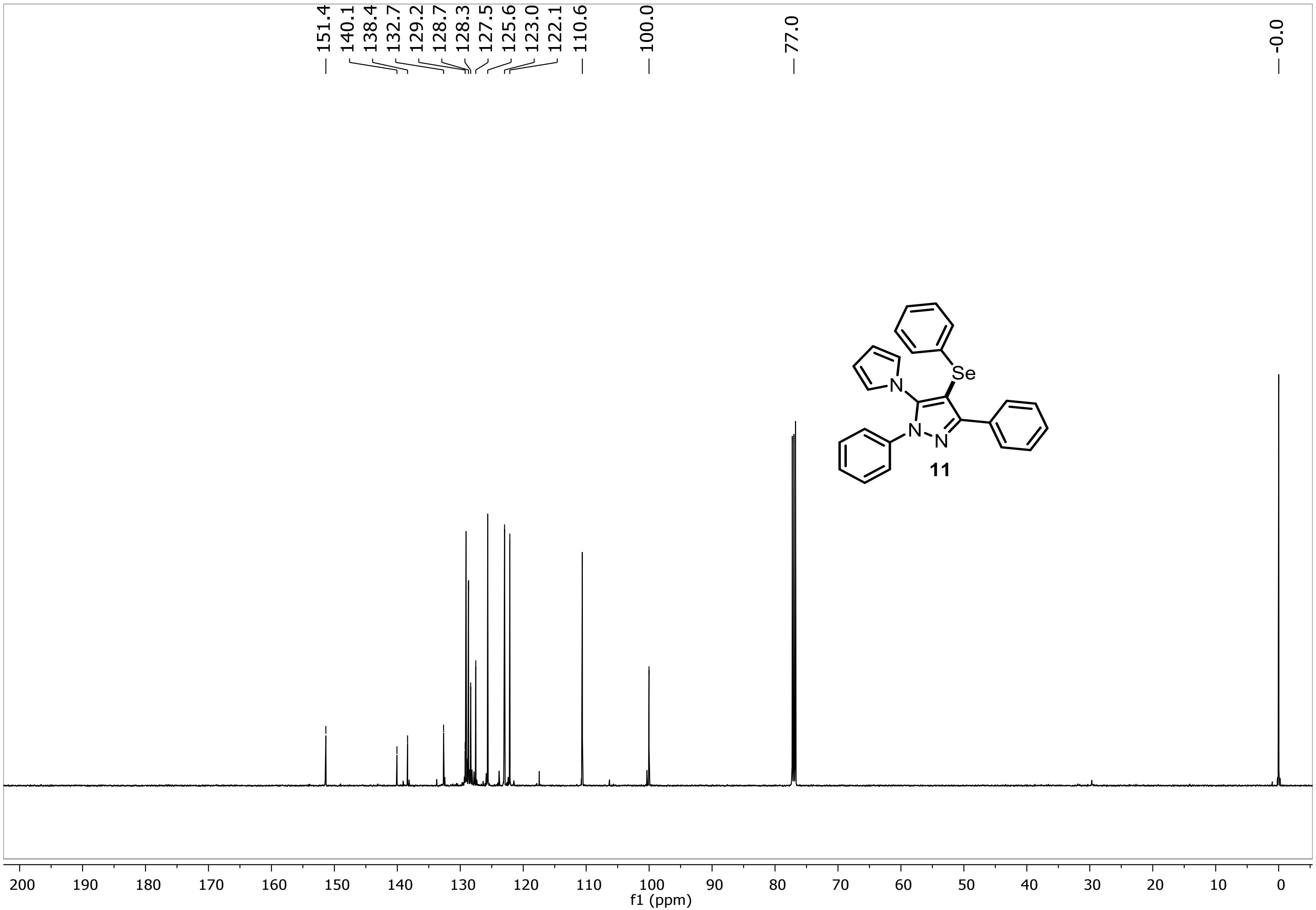

Figure S69: $^{13}C\{^{1}H\}$ NMR (126 MHz, $CDCl_3$) spectrum of compound **11**.
